# Molecular and Morphological Study of Leaping Frogs (Anura, Ranixalidae) with Description of Two New Species

**DOI:** 10.1371/journal.pone.0166326

**Published:** 2016-11-16

**Authors:** Sonali Garg, S. D. Biju

**Affiliations:** Systematics Lab, Department of Environmental Studies, University of Delhi, Delhi, 110 007, India; Leibniz-Institute of Freshwater Ecology and Inland Fisheries, GERMANY

## Abstract

The monotypic anuran family Ranixalidae is endemic to India, with a predominant distribution in the Western Ghats, a region that is home to several unique amphibian lineages. It is also one of the three ancient anuran families that diversified on the Indian landmass long before several larger radiations of extant frogs in this region. In recent years, ranixalids have been subjected to DNA barcoding and systematic studies. Nearly half of the presently recognized species in this family have been described over the last three years, along with recognition of a new genus to accommodate three previously known members. Our surveys in the Western Ghats further suggest the presence of undescribed diversity in this group, thereby increasing former diversity estimates. Based on rapid genetic identification using a mitochondrial gene, followed by phylogenetic analyses with an additional nuclear gene and detailed morphological studies including examination of museum specimens, new collections, and available literature, here we describe two new species belonging to the genus *Indirana* from the Western Ghats states of Karnataka and Kerala. We also provide new genetic and morphological data along with confirmed distribution records for all the species known prior to this study. This updated systematic revision of family Ranixalidae will facilitate future studies and provide vital information for conservation assessment of these relic frogs.

## Introduction

Laurent [1] proposed the name ‘*Indirana*’ (presumably, *Indi* for Indian, and *rana* for frogs) for the Indian endemic members of subgenus *Discodeles* Boulenger 1920, and recognized it as a distinct genus. Subsequently, this radiation was recognized worthy of higher taxonomic considerations [2–4], formally being elevated to the rank of a distinct family Ranixalidae [5]. Until recently, *Indirana* Laurent 1986 was considered as the sole generic representative in this family. Three of the previously known members have been transferred to a new genus *Sallywalkerana* Dahanukar, Modak, Krutha, Nameer, Padhye and Molur 2016 proposed within the same family. Together, the two genera presently include 14 recognized species, of which seven were described by European Zoologists [6–8] between 128–140 years ago, followed by one description each by Dubois [9], Padhye *et al*. [10] and Modak *et al*. [11], and four by Dahanukar *et al*. [12]. Two species described by Rao [13] are currently not recognized as valid [12]. Ever since their original descriptions, several members of this family have been assigned to various widespread genera such as *Polypedates* [6], *Ixalus* [6], *Rana* [7, 8], *Rana* (*Discodeles*) [13–15] and *Philautus* [13]. More than a century after the description of the oldest known species (*Polypedates beddomii* Günther 1876), taxonomic clarity initiated only with the proposal of a new genus *Indirana* (and *Ranixalus* Dubois 1986, later synonymized [16]). Several taxa were subsequently transferred to genus *Indirana* [17] resulting in stable generic placements. Commonly, ranixalids have been termed as Leaping frogs [18], a name which is appropriate for their behavior of leaping long distances after initiating salutatory locomotion, upon being approached.

Phylogenetic studies have provided molecular support for the distinct evolutionary position of Ranixalidae within the dominant Old World frog family Ranidae (*sensu* [19]) (e.g., [5, 19–27]). Roelants *et al*. [23] further highlighted members of this family as ancient frogs that originated prior to several recognized subfamilies in Ranidae (*sensu* [19]) (e.g., dicroglossids, ranids and rhacophorids), and showed its sister relationship with Micrixalidae. The first attempt to investigate the genetic diversity within this endemic family was made by Nair *et al*. [28–29] and subsequently by Modak *et al*. [30]. These studies suggested that many of the presently recognized species are likely to be complexes of several cryptic species, following which six new *Indirana* species have been described [10–12]. Even though a considerable amount of molecular data from populations across the Western Ghats became available in the recent years [10, 11, 28–30], species identity in most cases remained doubtful due to various persistent sources of confusion, including the status and unavailability of some type specimens [31]. A taxonomic review was provided by Nair *et al*. [32] followed by an integrated systematic revision by Dahanukar *et al*. [12]. However, none of these studies have been able to provide new collections for the poorly known *Indirana longicrus* and *I*. *tenuilingua*, for which the type material is considered long lost [33]. Both *Indirana longicrus* and *I*. *tenuilingua* were suggested to be considered as incertae sedis, the former under the order Anura and latter under the genus *Indirana*, until further information becomes available on these taxa [12].

The Western Ghats of India is a globally recognized biodiversity hotspot, which has been celebrated as being particularly rich in amphibian diversity and high family-level endemism [23, 31, 34, 35]. Two monotypic anuran families (Micrixalidae and Nasikabatrachidae) and twelve amphibian genera are exclusively found in the Western Ghats mountain ranges. Though Ranixalidae is largely regarded as a radiation restricted to the Western Ghats, the distribution of this endemic family is known to extend to the Eastern Ghats. The Western Ghats hotspot region has also witnessed a rapid increase in the number of new amphibian species over the past decade [36], with 223 species presently included in the amphibian inventory of this region [37, 38]. Further efforts to have a near-complete inventory of existing species diversity in various amphibian groups, especially ancient and endemic lineages such as ranixalids, is important for effective conservation of amphibians of the Western Ghats [36].

Our extensive fieldwork in the Western Ghats and particular sampling of ranixalid frogs from the entire region, prompted us to investigate the existing species diversity and genetic relationships within members of this family. Using samples from all the species described till date, we employed the DNA barcoding approach for rapid genetic identification, followed by phylogenetic analyses, to explore the possible existence of cryptic taxa. A combined understanding from the molecular and morphological data suggests that there are still several undescribed cryptic species in this morphologically conserved group, of which two are formally described here.

## Materials and Methods

### Ethics statement

This study was conducted with permissions and following guidelines from the responsible authorities in the State Forest Departments (Maharashtra, Karnataka, Kerala, Tamil Nadu), Ministry of Environment, Forest and Climate Change, Government of India. Our protocols of collection and research complied with the provisions of the Wildlife (Protection) Act 1972, Government of India. Specific methods of collection, euthanasia, tissue sampling and fixation used in our study followed the guidelines for use of live amphibians and reptiles in field research by the American Society of Ichthyologists and Herpetologists (ASIH), and were approved by the ethical committee of Department of Environmental Studies, University of Delhi.

### Field surveys and specimen collection

Extensive field expeditions and sampling was carried out between the years 2000–2015 at 93 localities in the Western Ghats (S1 Table). Ranixalid frogs are typically forest dwelling and usually found in close association with streamside vegetation, damp leaf litter or rock cutting and crevices. Hence, our search efforts were focused on specific habitats inside primary and secondary forest areas. Several populations were collected from disturbed or fragmented habitats in secondary forests. Collections were primarily undertaken at night by locating calling males, or by opportunistic searches during both night and day. Sampled animals were euthanized in MS-222, fixed in 4% formalin and preserved in 70% ethanol. Prior to fixation, small portions of the thigh muscle or liver tissues were preserved in absolute ethanol for genetic studies. Tissue samples were stored at -20° C at Systematics Lab, University of Delhi (SDBDU). Type specimens are deposited at Zoological Survey of India–Western Ghats Field Research Centre (ZSI–WGRC) Kozhikode, and referred specimens are available at SDBDU. Geographical coordinates and elevation at each sampling locality were recorded using Garmin 76CSx. S1 Table provides the details of sampling localities along with the species recorded. Maps were prepared using QGIS (http://www.qgis.org).

### Taxon sampling and DNA protocols

This study includes 269 ranixalid samples representing all known species in the family. One *Nyctibatrachus* sp. served as the outgroup taxon for phylogenetic analyses. For 134 ingroup individuals, sequence data was obtained from the GenBank (S2 Table). Genomic DNA was extracted from 135 new individuals, using Qiagen DNeasy tissue kit (Qiagen, Inc., Valencia, CA, USA) following manufacturer’s protocol. Fragments of the mitochondrial 16S rRNA (16S) gene (≈540 bp) from all samples and the nuclear Recombination activating gene 1 (Rag1) (≈555 bp) from 16 representative taxa were PCR-amplified using previously published primer sets 16Sar and 16Sbr [39], and Rag1-C and Rag1-E [35], respectively. Sequencing was performed on both strands using BigDye Terminator v3.1 Cycle Sequencing kit and ABI 3730 automated DNA sequencer (Applied Biosystems). Raw sequences were checked and assembled in ChromasPro v1.4 (Technelysium Pty Ltd.), and imported in MEGA 5.2 [40] for creating an alignment using the ClustalW option. Ambiguous sections in the non-coding mitochondrial DNA sequences were manually identified by eye and excluded from phylogenetic analyses. The coding nuclear gene sequences were optimized by comparison with amino acids. Sequences generated as part of this study are deposited in the GenBank under accession numbers KX966028–KX966179. Sequence details are provided in S2 Table.

### Molecular analyses

Alignment of the 16S sequences contained 534 characters, of which 500 were used for constructing a Neighbour-Joining (NJ) tree using the Kimura 2-parameter (K2P) distance model in PAUP* 4.0b10 [41]. The NJ tree was only used as a tool to visualize genetic differences between species, as defined in this work, and to identify putative new species. Uncorrected pairwise genetic distances (p-distances) were computed in PAUP* 4.0b10 [41] using all characters. Based on the grouping of individuals in the NJ tree, intraspecific pairwise comparisons between individuals or populations of the same species, and interspecific pairwise comparisons between species, were calculated to assess levels of genetic divergence reliable for species delineation within the family [42]. Frequency of the genetic distances was plotted as a histogram to visualize the intraspecific and interspecific genetic variation.

In order to infer phylogenetic support for the known and candidate species, Maximum Likelihood (ML) tree was constructed using a combined mitochondrial and nuclear DNA dataset comprising of 1063 nucleotides for selected 16 ingroup taxa and the outgroup. The best-fit model of DNA evolution was determined by implementing the Akaike Information Criterion in ModelTest 3.4 [43]. Heuristic ML searches were performed in PAUP* 4.0b10 [41] using the GTR+G+I model with all parameters estimated. Clade support under the Maximum Likelihood criteria was evaluated with 1000 rapid bootstrap replicates, using RAxML 7.3.0 [44, 45] as implemented in raxmlGUI 1.1 [46]. Bayesian analyses were performed using the same model in MrBayes 3.1.2 [47]. Two parallel runs of four MCMC chains were executed for five million generations and the first 25% of trees sampled at a frequency of 100 generations were discarded as burn-in. Bayesian Posterior Probabilities (BPP) for the clades were estimated from a majority-rule consensus tree.

### Adult morphology

Species identification was done using an integrative molecular and morphological approach. Molecular operational taxonomic units (MOTUs) were morphologically compared with all available types and new collections from the type localities. Sex and maturity were determined by examining the gonads through a small ventral incision. Only adult animals were used for morphometrics and morphological comparisons. For the convenience of discussion, ranixalid species were grouped as small (20–40 mm), medium (41–55 mm) and large (56–70 mm). Measurements and associated terminology follow Biju *et al*. [48, 49]. The measurements were taken to the nearest 0.1 mm, using a digital slide-caliper or a binocular microscope with a micrometer ocular. Digit number is represented by roman numerals I–V. The term shank refers to the lower part of the leg containing the tibia, and the term thigh for the upper part containing the femur. The webbing formulae follow Savage and Heyer [50] as modified by Myers and Duellman [51]. The extent of webbing relative to subarticular tubercles is described by numbering the tubercles 1–3, starting from the toe discs. All measurements provided in the taxonomy section are in millimeters. In the Material studied section, the following abbreviations after specimen numbers refer to: HT (holotype), PT (paratype), LT (lectotype) and RS (referred specimen). For museums and frequently used terms, abbreviations are as follows: BNHS (Bombay Natural History Society, Bombay, India), DU (University of Delhi, Delhi, India), MNHN (Muséum National d’Histoire Naturelle, Paris), NHM (Natural History Museum, formerly British Museum (Natural History)), BMNH, (British Museum (Natural History), London, United Kingdom), International Union for Conservation of Nature (IUCN), SDB (S D Biju), SG (Sonali Garg).

### Nomenclatural acts

The electronic edition of this article conforms to the requirements of the amended International Code of Zoological Nomenclature (ICZN), and hence the new names contained herein are available under that Code from the electronic edition of this article. This published work and the nomenclatural acts it contains have been registered in ZooBank, the online registration system for the ICZN. The ZooBank LSIDs (Life Science Identifiers) can be resolved and the associated information viewed through any standard web browser by appending the LSID to the prefix “http://zoobank.org/”. The LSID for this publication is: urn:lsid:zoobank.org:pub:C264EC2C-896A-40EB-AD23-1E0AC587BC1C. The electronic edition of this work was published in a journal with an ISSN, which has been archived and is available from the following digital repositories: PubMed Central, LOCKSS.

## Results

### DNA barcoding for rapid genetic identification

**Fig 1:**
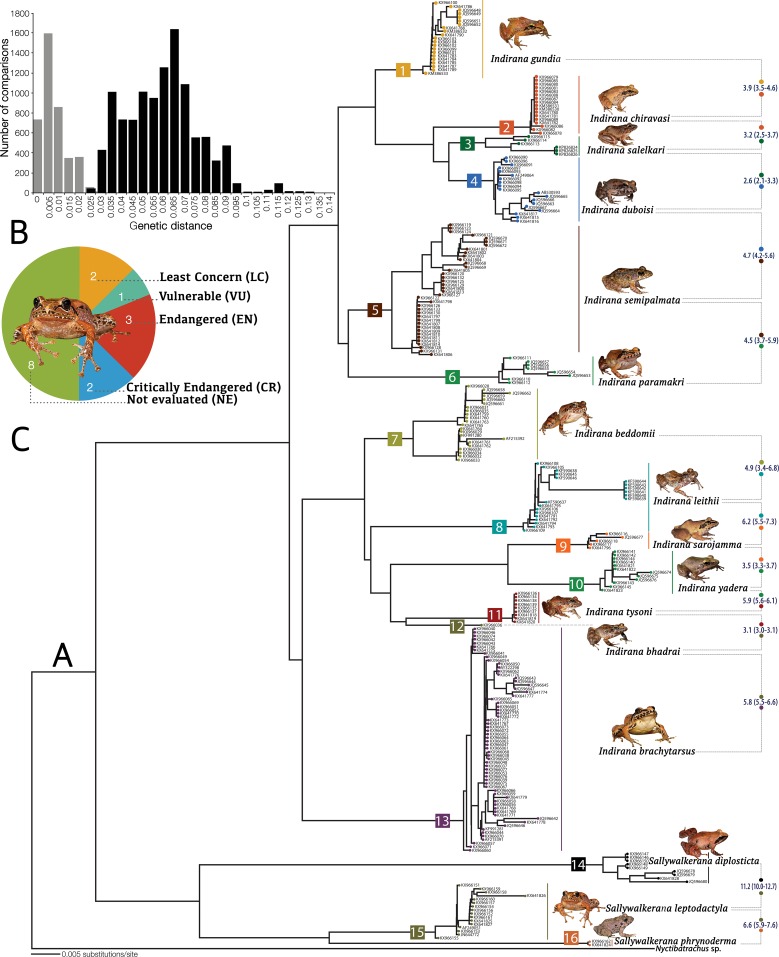
DNA barcoding and status of IUCN assessment of ranixalid species of the Western Ghats. (A) Neighbor-Joining (NJ) tree based on Kimura-2-parameter model for 16S mitochondrial gene sequences from 269 ranixalid samples, representing all previously known and newly recognized species from the Western Ghats. Terminal node labels indicate voucher numbers that are cross-referenced in S2 Table; clade numbers are indicated towards the internal nodes of the tree; mean and range of interspecific genetic distances (in percent) are shown between subsequent clades. (B) Frequency of intra (grey) and interspecific (black) uncorrected pairwise distances for 16S. (C) Number of ranixalid species in the different IUCN categories [66].

The Neighbour Joining (NJ) tree (Fig 1A) showed 16 major clusters corresponding to 11 known *Indirana* species: *I*. *gundia* (node 1), *I*. *chiravasi* (node 2), *I*. *salelkari* (node 3), *I*. *duboisi* (node 4), *I*. *semipalmata* (node 5), *I*. *beddomii* (node 7), *I*. *leithii* (node 8), *I*. *sarojamma* (node 9), *I*. *yadera* (node 10), *I*. *tysoni* (node 11), *I*. *brachytarsus* (node 13); two candidate species (nodes 6 and 12); and three known *Sallywalkerana* species: *S*. *diplosticta* (node 14), *S*. *leptodactyla* (node 15) and *S*. *phrynoderma* (node 16). Based on detailed morphological studies (see ‘Morphology and recognition of species‘), we formally describe two new *Indirana* species as–*Indirana bhadrai* **sp. nov.** and *Indirana paramakri* **sp. nov.**

Uncorrected intraspecific genetic distance (S3 Table) was the highest at 3.5% for *Indirana beddomii*. Minimum interspecific distance was observed between two previously known species *I*. *duboisi* and *I*. *salelkari*, with the least divergence of 2.1% and mean divergence of 2.6% (S4 Table). In general, interspecific distances were low (minimum 2.1–2.7%) in the *Indirana semipalmata* group (see ‘Taxonomic grouping of species‘). Despite relatively lower genetic distances observed between some previously known species, we applied a conservative approach while delineating candidate species based on genetic distances. In the present study, we considered minimum 3.0% divergence value as reliable for delineating candidate species, since these values ensured no overlap between the maximum intraspecific pairwise distances within species and minimum interspecific distances between species (Fig 1B). Both the new species (nodes 6 and 12) we describe were delineated at a minimum and mean genetic distance of 3.0% from their closest relatives. These results were found to be largely consistent with genetic distances observed at species-level in other closely related groups in this region (e.g., [36, 49, 52, 53]). The maximum sequence divergence in our dataset was between *Sallywalkerana diplosticta* and *S*. *phrynoderma* (range 11.5–12.7%, mean 12.2%), and between *S*. *diplosticta* and *S*. *leptodactyla* (range 10.0–12.7%, mean 11.2%). For detailed genetic comparisons, see S3 and S4 Tables and the ‘Genetic divergence’ section of each species in the taxonomy section.

### Phylogenetic analyses

**Fig 2:**
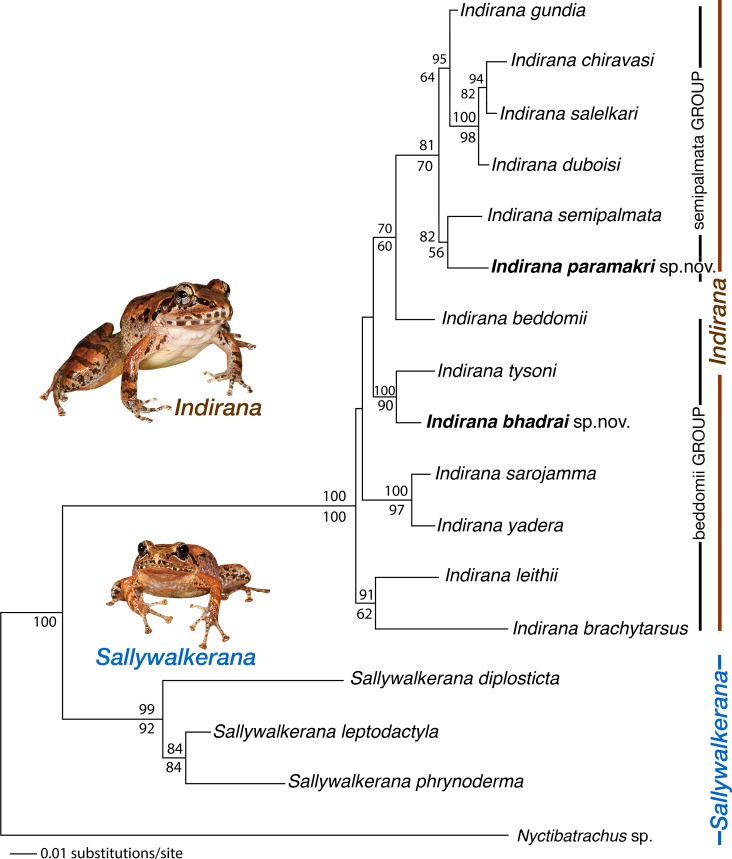
Maximum likelihood tree for a combined mitochondrial (16S rRNA) and nuclear (Rag1) DNA data set of 1063 bp showing phylogenetic relationships among 16 recognized ranixalid species and one outgroup species. Bayesian Posterior Probabilities and RaxML Bootstrap values >50% are indicated above and below the branches, respectively. Morphological groups are referenced in the text.

The Maximum Likelihood and Bayesian analyses recovered all the 16 recognized ranixalid species as distinct clades. Species-level relationships in the Maximum Likelihood and Bayesian frameworks were largely found to be in agreement (Fig 2). For the putative new species, sister relationship between *Indirana bhadrai* sp. nov. and *I*. *tysoni* (100 BPP, 90 BS) received high support, and the relationship of *Indirana paramakri* sp. nov. with *I*. *semipalmata* was recovered with moderate support (82 BPP, 56 BS). Members of the morphologically recognized *Indirana beddomii* group (*I*. *beddomii*, *Indirana bhadrai* sp. nov., *I*. *brachytarsus*, *I*. *leithii*, *I*. *sarojamma*, *I*. *tysoni* and *I*. *yadera*) appear to be paraphyletic but these relationships remained unresolved. On the hand, *Indirana semipalmata* group (including *I*. *chiravasi*, *I*. *duboisi*, *I*. *gundia*, *Indirana paramakri* sp. nov., *I*. *salelkari* and *I*. *semipalmata*) and genus *Sallywalkerana* (including *S*. *diplosticta*, *S*. *leptodactyla* and *S*. *phrynoderma*) formed monophyletic clades. However, the phylogenetic analyses performed in this study were only to infer molecular support for the new species and not for taxonomic grouping of species, which was primarily based on morphological characters (see ‘Taxonomic grouping of species’).

### Morphology and recognition of species

Detailed comparison of morphological data corroborated the molecular evidence in our study, thereby enabling proper identification of all sampled populations in the family Ranixalidae along with description of two new species in the genus *Indirana*.

The new species were compared with all available types, and with recently collected new specimens from the type localities. Since femoral gland is a secondary sexual character in males, which may be more prominent during the breeding season, its presence or absence is not considered as a diagnostic character in the present study. Among the specimens examined in our study, we observed femoral glands on the ventral surface of thighs in *Indirana chiravasi*, *I*. *duboisi*, *Indirana paramakri* sp. nov., *I*. *salelkari*, *I*. *semipalmata*, *I*. *yadera* and *Sallywalkerana phrynoderma* (S1 Fig), but they were absent in *Indirana beddomii*, *I*. *brachytarsus*, *I*. *leithii*, *I*. *sarojamma*, *I*. *tysoni*, *Sallywalkerana diplosticta* and *S*. *leptodactyla*. However, absence of femoral glands in all seasons could be confirmed only in *Indirana brachytarsus*, *I*. *tysoni* and *Sallywalkerana diplosticta*. Further studies will be required to confirm the presence or absence of femoral glands in ranixalid species, during the breeding as well as non-breeding season.

### Taxonomic treatment

Family. Ranixalidae Dubois 1987

Type genus. *Indirana* Laurent 1986

Genus included. *Indirana* Laurent 1986, *Sallywalkerana* Dahanukar, Modak, Krutha, Nameer, Padhye and Molur 2016

Common name. Leaping Frogs [18]

Etymology. The family name Ranixalidae is derived from two words, *Rana* meaning ‘frog’ in Latin, and the word *ixalus*, which is often used as a suffix in rhacophorid generic names.

**Fig 3:**
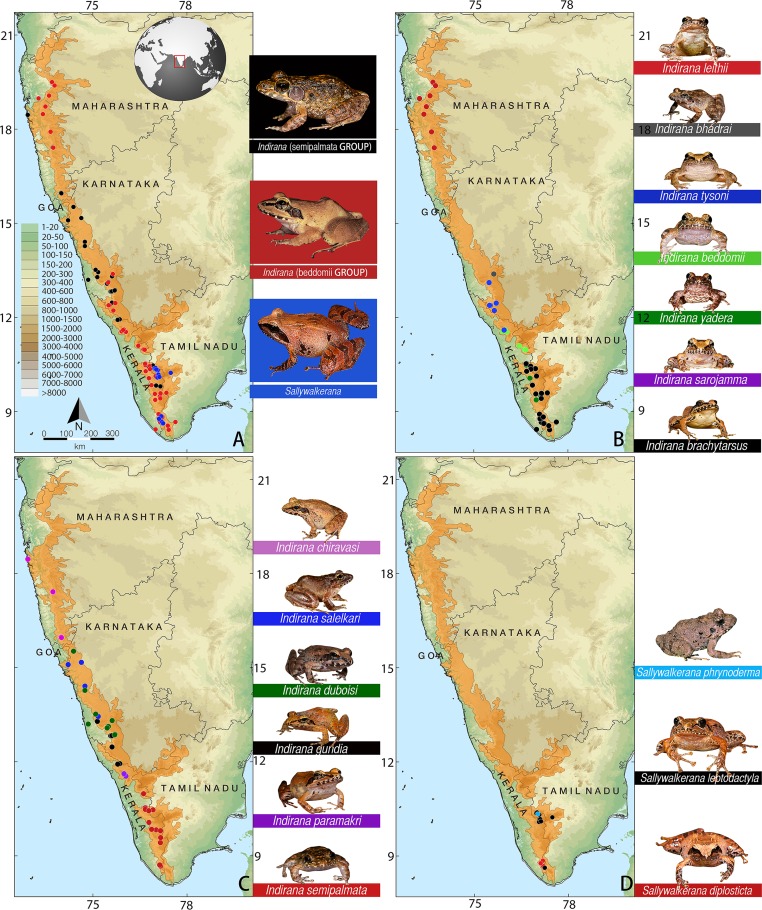
Distribution localities of Leaping frog species reported from the Western Ghats of India in the present study. (A) Sampling localities of all species. Species in genus *Indirana* are categorized into two morphological groups recognized in the study. (B) Sampling localities of seven species in the *Indirana beddomii* group. (C) Sampling localities of six species in the *Indirana semipalmata* group. (D) Sampling localities of three species in genus *Sallywalkerana*. Colors of sampling localities correspond with group/species labels on the right panel of each map. The Western Ghats biodiversity hotspot region is shaded orange.

Distribution. Genus *Indirana* is endemic to Peninsular India, predominantly the Western Ghats states of Tamil Nadu, Kerala, Karnataka, Goa, Maharashtra, and southern Gujarat, and further extends up to Eastern Ghats in the state of Andhra Pradesh (Fig 3). Reports of *Indirana* frogs from Madhya Pradesh require verification. Genus *Sallywalkerana* is restricted to regions south of Palghat gap in the Western Ghats states of Kerala and Tamil Nadu (Fig 3).

Genus. *Indirana* Laurent 1986

Type species. *Polypedates beddomii* Günther 1876 [= *Indirana beddomii* (Günther 1876)]

Species included. *Indirana beddomii* (Günther 1876) *Indirana bhadrai* **sp. nov.**, *I*. *brachytarsus* (Günther 1876), *I*. *chiravasi* Padhye Modak and Dahanukar 2014, *I*. *duboisi* Dahanukar, Modak, Krutha, Nameer, Padhye and Molur, 2016, *I*. *gundia* (Dubois 1986), *I*. *leithii* (Boulenger 1888), *Indirana paramakri* **sp. nov.**, *I*. *salelkari* Modak, Dahanukar, Gosavi, and Padhye 2015, *I*. *sarojamma* Dahanukar, Modak, Krutha, Nameer, Padhye and Molur 2016, *I*. *semipalmata* (Boulenger 1882), *I*. *tysoni* Dahanukar, Modak, Krutha, Nameer, Padhye and Molur, 2016, *I*. *yadera* Dahanukar, Modak, Krutha, Nameer, Padhye and Molur 2016.

Note. Dahanukar *et al*. [12] have considered *Rana* (*Discodeles*) *tenuilingua* Rao 1937 [= *Indirana tenuilingua* (Rao 1937)] as incertae sedis under the genus *Indirana*, and *Philautus longicrus* Rao 1937 [= *Indirana longicrus* (Rao 1937)] as incertae sedis under the order Anura. Further studies will be required to confirm the taxonomic status of these two taxa (see ‘Discussion‘).

Common name. Indian Leaping Frogs

Etymology. The generic name *Indirana* is probably derived from two words, *Indi* for ‘Indian’, and *Rana* meaning ‘frog’ in Latin.

Distribution. The geographical range extends from Kiriparai (Kanyakumari district), Tamil Nadu state in the south, through the states of Kerala, Karnataka, Goa, Maharashtra, upto Surat Dangs in southern Gujarat [54], and further extends to Nallamala hills (Eastern Ghats) in Andhra Pradesh state [55]. Reports from Madhya Pradesh [56, 57] require further verification due to lack of voucher specimens. For distribution records of *Indirana* reported in the present study see Fig 3 and S1 Table.

Salient morphological characters. Male SVL 23.0–46.0 mm, female SVL 30.0–62.0 mm; pupil oval; presence of discontinuous dorsal skin folds or prominent skin warts; vomerine teeth present; tongue emarginated posteriorly with lingual papillae; tympanum distinct; supratympanic fold present; presence of a dark band that extends from the tip of snout or nostril, ending near the axilla on either sides of the head; tips of fingers and toes with discs having distinct dorsoterminal grooves; interdigital webbing absent between fingers; moderate to extensive webbing between toes.

#### Taxonomic grouping of species

Based on morphological similarities between species, we identify two major species groups in the genus *Indirana*–*Indirana beddomii* group and *Indirana semipalmata* group. For detailed morphological characters and species included, see the group-wise species accounts in the below section.

**Fig 4:**
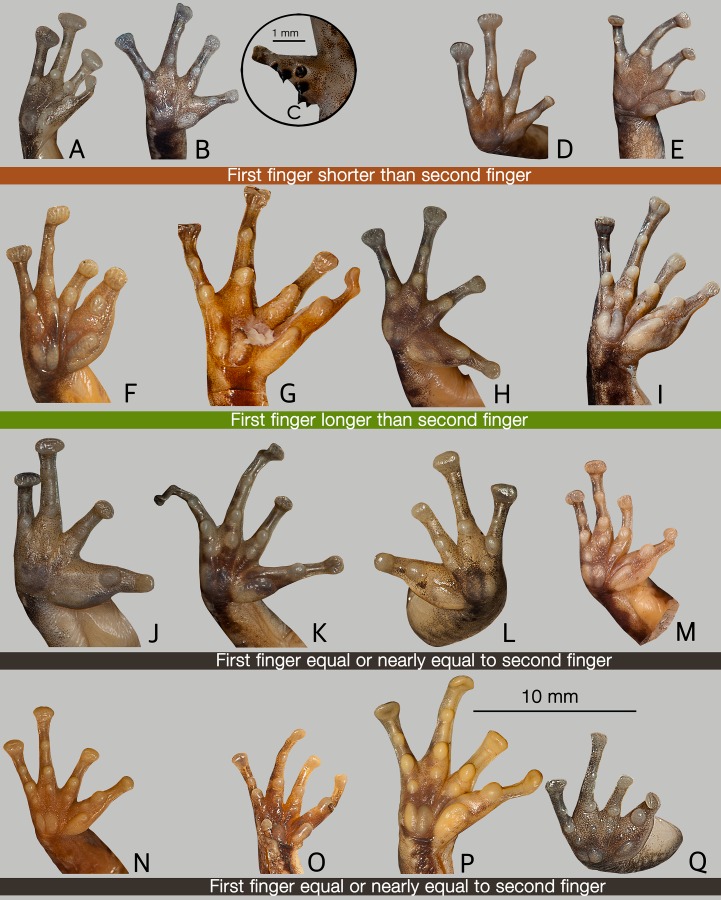
Ventral view of hands showing the relative length of first and second finger in ranixalid species. (A–E) Species with first finger shorter than second finger. (A) *Indirana leithii*, SDBDU 2014.2515. (B, C) *Sallywalkerana diplosticta*, SDBDU 2015.2957. (B) Ventral surface. (C) Spinular projections on dorsal surface of first finger. (D) *S*. *leptodactyla*, SDBDU 2011.1058A. (E) *S*. *phrynoderma*, SDBDU 2002.1181. (F–I) Species with first finger longer than second finger. (F) *Indirana beddomii*, SDBDU 2010.225. (G) *I*. *sarojamma*, SDBDU 2002.334. (H) *I*. *tysoni*, SDBDU 2012.74. (I) *I*. *yadera*, SDBDU 2012.2744. (J–Q) Species with first finger equal or nearly to second finger. (J) *I*. *brachytarsus*, SDBDU 2015.2931. (K) *Indirana bhadrai* sp. nov., ZSI/WGRC/V/A887. (L) *I*. *Chiravasi*, SDBDU 2014.2483. (M) *I*. *Duboisi*, SDBDU 2003.1086. (N) *I*. *Gundia*, MNHN 1985.0633. (O) *Indirana paramakri* sp. nov., SDBDU 2005.3741. (P) *I*. *Salelkari*, SDBDU 2011.1330. (Q) *I*. *Semipalmata*, SDBDU 2015.3035.

**Fig 5:**
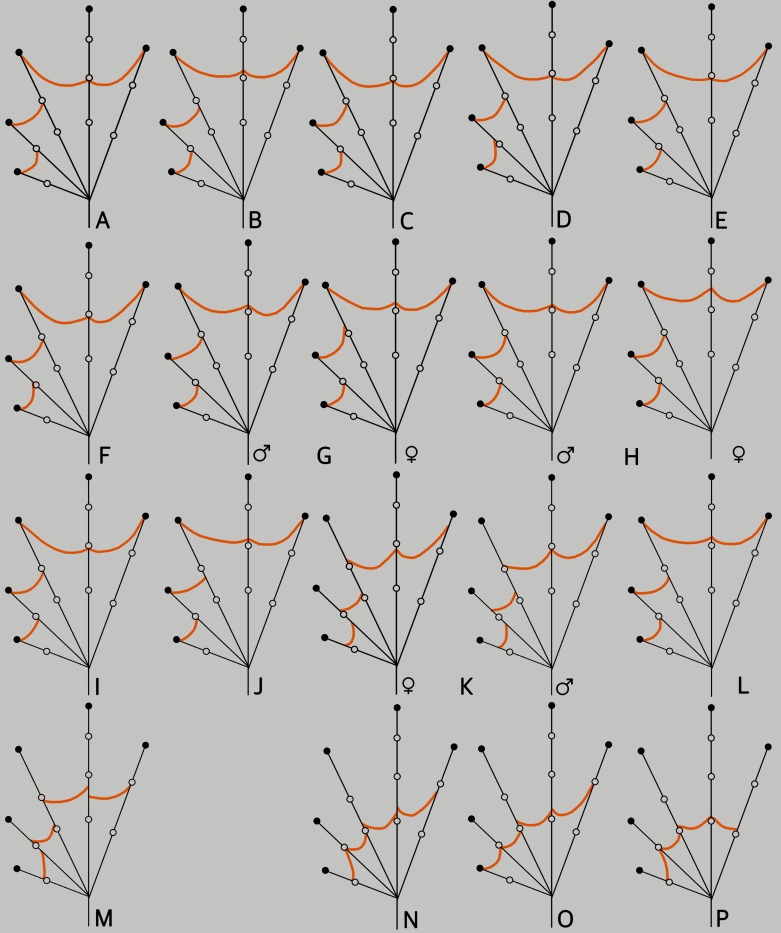
Schematic illustration of foot webbing in ranixalid species. (A–G) *Indirana beddomii* group. (A) *I*. *beddomii*, lectotype of *Polypedates beddomii*, NHM 74.4.29.208 (ex BMNH 1947.2.27.72), female. (B) *Indirana bhadrai* sp. nov., holotype, ZSI/WGRC/V/A887, female. (C) *I*. *brachytarsus*, lectotype of *Polypedates brachytarsus*, NHM 74.4.29.1307 (ex BMNH 1947.2.27.92), female. (D) *Indirana leithii*, holotype of *Rana leithii*, NHM 69.8.28.50 (ex BMNH 1947.2.28.17), female. (E) *Indirana sarojamma*, SDBDU 2002.516, female. (F) *I*. *tysoni*, SDBDU 2012.73, female. (G) *I*. *yadera*, SDBDU 2012.2744, male (left), SDBDU 2015.2984, female (right). (H–M) *Indirana semipalmata* group. (H) *I*. *chiravasi*, SDBDU 2014.2423, male (left), SDBDU 2015.3087, female (right). (I) *I*. *duboisi*, SDBDU 2003.1086, male. (J) *I*. *gundia*, holotype of *Ranixalus gundia*, MNHN 1985.0633, male. (K) *Indirana paramakri* sp. nov., SDBDU 2015.3741, male (left), ZSI/WGRC/V/A888, female (right). (L) *I*. *salelkari*, SDBDU 2011.1330, female. (M) *I*. *semipalmata*, lectotype of *Rana semipalmata*, NHM 74.4.29.605 (ex BMNH 1947.2.29.50), female. (N–P) Genus *Sallywalkerana*. (N) *S*. *diplosticta*, lectotype of *Ixalus diplostictus*, NHM 74.4.29.1412 (ex BMNH 1947.2.2.21), female. (O) *Sallywalkerana leptodactyla*, lectotype of *Rana leptodactyla*, NHM 74.4.29.593 (ex BMNH 1947.2.29.39), female. (P) *Sallywalkerana phrynoderma*, lectotype of *Rana phrynoderma*, NHM 82.2.10.21 (ex BMNH 1947.2.3.8), female.

*Indirana beddomii* group. Morphologically this group can be distinguished from *Indirana semipalmata* group by the following suite of characters: small to large-sized adults (males: SVL 25.0–39.0 mm, females: SVL 31.0–63.0 mm), first finger longer than second finger, except in *Indirana bhadrai* sp. nov., *I*. *brachytarsus* and *I*. *leithii* (Fig 4), tympanum well-developed, smaller or nearly equal to horizontal diameter of eye (S2 Fig), and webbing on fourth toe extending up to the second subarticular tubercle on either side (Fig 5).

Members included. *Indirana beddomii*, *Indirana bhadrai* sp. nov., *I*. *brachytarsus*, *I*. *leithii*, *I*. *sarojamma*, *I*. *tysoni* and *I*. *yadera*.

***Indirana beddomii*** (Günther 1876)

Beddome's Leaping Frog [58]

(Figs 1–6; S2–S4 Figs; S1–S4 Tables; S1 File)

Original name and description. *Polypedates beddomii* Günther 1876. Third report on collections of Indian reptiles obtained by the British Museum, *Proceedings of the Zoological Society of London*, 1876 “1875”: 571–572. Lectotype. NHM 74.4.29.208 (ex BMNH 1947.2.27.72), designated by Dahanukar *et al*. [12], adult female, from “Malabar”, figured in Günther 1876: LXIII. Fig B. Type locality. “Malabar”, India, according to original description. Current status of specific name. Valid name, as *Indirana beddomii* (Günther 1876).

Comments. The original description [6] mentions several specimens from four different localities—Malabar, Travancore, Anamallays and Sevagherry, all from Col. Beddome’s collection. We examined all the specimens (five males, six females and nine sub-adults) available at NHM, and found NHM 74.4.29.208 (ex BMNH 1947.2.27.72) from “Malabar” to be in agreement with the original description, with respect to the snout-vent size (“Length of body 55 mm”), snout shape (“rather obtuse”) and webbing on foot (“toes two thirds webbed”). It was also found to be in well-preserved condition (S4 Fig). Dahanukar *et al*. [12] designated this specimen as the lectotype.

Genetic divergence. Intraspecific genetic variation within populations of *I*. *beddomii* is 1.2 ± 0.9% (range 0–3.5%, *N* = 22) for 16S mitochondrial gene sequences. *Indirana beddomii* is closely related to members of the *Indirana beddomii* group (Figs 1 and 2); differs from *I*. *bhadrai* by mean genetic divergence of 4.2 ± 0.3% (range 3.7–5.0%, *N* = 22); from *I*. *brachytarsus* by mean genetic divergence of 6.3 ± 0.4% (range 5.5–7.7%, *N* = 1408); from *I*. *leithii* by mean genetic divergence of 4.9 ± 0.7% (range 3.4–6.8%, *N* = 440); from *I*. *sarojamma* by mean genetic divergence of 5.5 ± 0.4% (range 5.0–7.0%, *N* = 110); from *I*. *tysoni* by mean genetic divergence of 4.7 ± 0.5% (range 4.0–5.9%, *N* = 198); and from *I*. *yadera* by mean genetic divergence of 6.2 ± 0.5% (range 5.5–8.5%, *N* = 264) (S3 and S4 Tables).

Distribution and natural history. *Indirana beddomii* is currently known only from the state of Kerala in the southern Western Ghats. In the present study, we found this species north of Palghat gap at Kakkayam, Settukunnu and Suganthagiri; and south of Palghat gap at Sairandhri (Silent Valley), Kuddam (Siruvani) and Pattiar (Siruvani) (Fig 3 and S1 Table). Specimens were located during night searches (between 17:00–19:00 hours) and were found either on earthen cutting inside evergreen forest or on forest floor in secondary forests. For details of the distribution records reported in this study see S1 Table, and for other genetically confirmed records see S2 Table.

For morphological comparison, description of lectotype, secondary sexual characters, variations, and list of specimens examined, see S1 File.

***Indirana bhadrai*** sp. nov.

urn:lsid:zoobank.org:act:9E20EF06-F9A4-4528-84C0-69C9CB4E0D91

**Fig 6:**
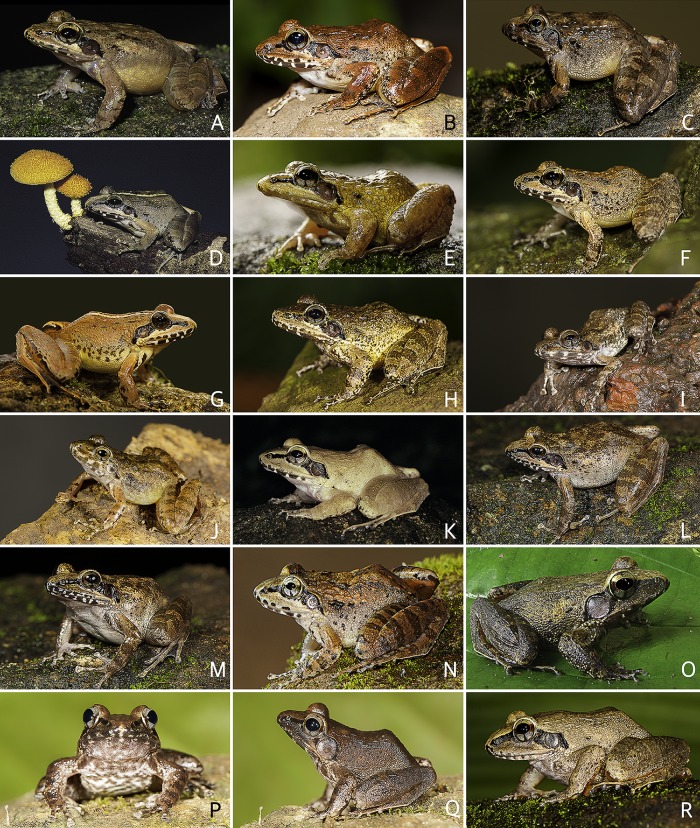
*Indirana beddomii* group in life. (A–B) *I*. *beddomii*. (A) An adult male (SDBDU 2010.225), from Kakkayam. (B) An adult female (SDBDU 2011.960), from Silent Valley. (C) *Indirana bhadrai*, holotype, an adult female (ZSI/WGRC/V/A887), from Muthodi. (D–H) *I*. *brachytarsus*. (D) An adult female (not preserved), from Ponmudi. (E) An adult male (SDBDU 2011.541), from Parambikulam. (F) An adult male (SDBDU 2006.4800), from Anchuruli. (G) An adult female (SDBDU 2011.280), from Pandimotta. (H) An adult male (SDBDU 2015.2995), from Upper Manalar. (I, J) *I*. *leithii*. (I) An adult female (SDBDU 2014.2514), from Matheran. (J) An adult male, (SDBDU 2011.1095), from Bhimashankar. (K) *I*. *sarojamma*, an adult female (SDBDU 2002.516) from Chathankod–Bonnacaud. (L–N) *I*. *tysoni*. (L, M) An adult female (SDBDU 2012.73), from Coorg. (L) Dorsolateral view. (M) Frontolateral view. (N) An adult male (SDBDU 2012.2228), from Coorg. (O–R) *I*. *yadera*. (O) An adult male (SDBDU 2012.2744), from Methooty. (P, Q) An adult male (SDBDU 2015.2984), from Kozhikana. (P) Frontal view. (Q) Dorsolateral view. (R) An adult female (SDBDU 2015.3155), from Vazhachal.

**Fig 7:**
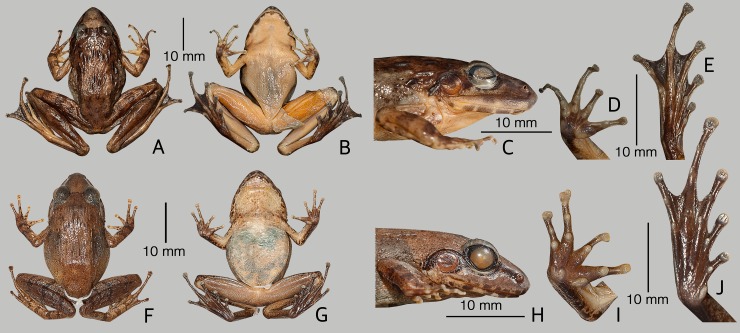
Two new *Indirana* species from the Western Ghats. (A–E) Holotype of *Indirana bhadrai* sp. nov., ZSI/WGRC/V/A887, female, in preservation. (F–J) Holotype of *Indirana paramakri* sp. nov., ZSI/WGRC/V/A888, female, in preservation. From left to right: Dorsal view, ventral view, lateral view of head, ventral view of hand, ventral view of foot.

Bhadra Leaping Frog

(Figs 1–7; Table 1; S2 and S3 Figs; S1–S4 Tables)

Etymology. The species name *bhadrai* is a noun in the genitive case, derived from Bhadra Wildlife Sanctuary, the region in which the type locality Muthodi forest is situated.

Holotype. ZSI/WGRC/V/A887, an adult female, from Muthodi forest, Bhadra Wildlife Sanctuary (12.2201°N 75.6557°E, 1176 m asl), Chikmagalur district, Karnataka state, collected by SDB and SG on 30 July 2012.

Referred specimen. SDBDU 2012.1471, an adult male, collected along with the holotype.

Comparison. Based on the overall morphology, *Indirana bhadrai* sp. nov. could be confused with *I*. *beddomii*, *I*. *brachytarsus*, *I*. *leithii*, *I*. *sarojamma*, *I*. *tysoni* and *I*. *yadera* within the *I*. *beddomii* group. However, *I*. *bhadrai* differs from all these species by its snout nearly pointed in dorsal view (vs. rounded in *I*. *beddomii*, *I*. *brachytarsus* and *I*. *leithii*; nearly truncate in *I*. *sarojamma*; sub-ovoid in *I*. *tysoni* and *I*. *yadera*), snout nearly acute in lateral view (vs. rounded in *I*. *beddomii*, *I*. *brachytarsus*, *I*. *leithii* and *I*. *tysoni*; obtuse in *I*. *sarojamma*), third toe webbing not extending up to the second subarticular tubercle on the inside, I1–2II1–2^1^/_4_III1–3^–^IV3^–^–1V (vs. up to the second subarticular tubercle in *I*. *beddomii*, *I*. *brachytarsus*, *I*. *leithii* and *I*. *tysoni*; and beyond the second subarticular tubercle in *I*. *yadera*, I1–2II1–2^−^III1–3^−^IV3^−^–1V) (Fig 5), first finger nearly equal to second finger, male FIL 5.1 mm, *N* = 1, female FIL 6.1 mm, *N* = 1 vs. male FIIL 5.2 mm, *N* = 1, female FIIL 6.2 mm, *N* = 1 (vs. longer in *I*. *beddomii*, *I*. *sarojamma*, *I*. *tysoni* and *I*. *yadera*: male FIL 4.1 ± 1.3 mm, *N* = 2, female FIL 6.9 ± 0.6 mm, *N* = 5 vs. male FIIL 3.1 ± 1.0 mm, *N* = 2, female FIIL 5.5 ± 0.3 mm, *N* = 5 in *I*. *beddomii*, male FIL 4.9 ± 0.1 mm, *N* = 2, female FIL 8.1 mm, *N* = 1 vs. male FIIL 3.8 ± 0.4 mm, *N* = 2, female FIIL 6.9 mm, *N* = 1 in *I*. *sarojamma*, male FIL 4.6 ± 0.4 mm, *N* = 4, female FIL 7.1 ± 0.3 mm, *N* = 2 vs. male FIIL 3.4 ± 0.1 mm, *N* = 4, female FIIL 5.9 ± 0.4 mm, *N* = 2 in *I*. *tysoni*, and male FIL 5.4 ± 0.5 mm, *N* = 3, female FIL 7.5 mm, *N* = 1 vs. male FIIL 3.9 ± 0.4 mm, *N* = 3, female FIIL 6.2 mm, *N* = 1 in *I*. *yadera*; and shorter in *I*. *leithii*: male FIL 2.6 ± 0.2 mm, *N* = 3, female FIL 3.4 ± 0.1 mm, *N* = 5 vs. male FIIL 3.8 ± 0.2 mm, *N* = 3, female FIIL 4.5 ± 0.3 mm, *N* = 5) (Fig 4), loreal region acute (vs. obtuse in *I*. *beddomii* and indistinct in *I*. *leithii*), and smaller adult size, male: SVL 30.2 mm, *N* = 1, female: SVL 38.7 mm, *N* = 1 (vs. larger, male: SVL 36.4 ± 2.6 mm, *N* = 2, female: SVL 61.2 mm, *N* = 1 in *I*. *sarojamma*). For more differences with *I*. *beddomii* see ‘Comparison’ of that species (S1 File).

For better clarity, we compare this new species with all other currently known species in this genus. *Indirana bhadrai* differs from the following members of *Indirana semipalmata* group: *I*. *gundia*, *I*. *paramakri* and *I*. *semipalmata* by its snout nearly pointed in dorsal view (vs. rounded in *I*. *gundia* and *I*. *semipalmata*, and sub-ovoid in *I*. *paramakri*), snout nearly acute in lateral view (vs. rounded in *I*. *gundia* and *I*. *semipalmata*), loreal region acute (vs. obtuse in *I*. *gundia* and *I*. *semipalmata*), first toe webbing extending up to the disc, I1–2II1–2^1^/_4_III1–3^−^IV3^−^–1V (vs. below in *I*. *gundia*, I1–2II1–2^1/4^III1–3^−^IV3^−^–1V; and just above the first subarticular tubercle in *I*. *semipalmata*, I2^−^–2^+^II2^−^–3^−^III2–3^1/4^IV3^1/2^–2V, and *I*. *paramakri*, I1^2/3^–2^+^II1^3/4^–2^3/4^III2^−^–3IV3–1^+^V); differs from *I*. *chiravasi* and *I*. *salelkari*, by its snout nearly pointed in dorsal view (vs. sub-ovoid in *I*. *chiravasi*, and rounded in *I*. *salelkari*), snout nearly acute in lateral view (vs. obtuse in *I*. *chiravasi*, and rounded in *I*. *salelkari*), and loreal region acute (vs. obtuse in *I*. *chiravasi* and *I*. *salelkari*); specifically differs from *I*. *chiravasi* by its third toe webbing below the first subarticular tubercle on the inside, I1–2II1–2^1^/_4_III1–3^−^IV3^−^–1V (vs. extending up to first subarticular tubercle, I1–2II1–2III1–3^−^IV3^−^–1V); differs from *I*. *duboisi* by its snout sub-ovoid in dorsal view (vs. nearly pointed), snout nearly acute in lateral view (vs. rounded), loreal region acute (vs. obtuse), and webbing between first, second and third toes not extending up to the disc on the outside, I1^2/3^–2^+^II1^3/4^–2^3/4^III2^−^–3IV3–1^+^V (vs. upto the disc, I1–2II1–2III1–3^−^IV3^−^–1V) (Fig 5).

Furthermore, *Indirana bhadrai* differs from members of the genus *Sallywalkerana* by its larger adult snout-vent size, male: SVL 30.2 mm, *N* = 1, female: SVL 38.7 mm, *N* = 1 (vs. smaller), first finger equal or nearly equal to second finger (vs. first finger shorter than second), and third toe webbing extending up to second subarticular tubercle on either side, I1–2II1–2^1^/_4_III1–3^−^IV3^−^–1V (vs. well below) (Fig 5).

Genetic divergence. Intraspecific genetic variation within populations of *Indirana bhadrai* could not be calculated since the present study included a single sample of this species. Based on 16S mitochondrial gene sequences and phylogenetic analyses, *I*. *bhadrai* is closely related to members of the *I*. *beddomii* group (Figs 1 and 2); differs from *I*. *brachytarsus* by mean genetic divergence of 5.8 ± 0.2% (range 5.5–6.6%, *N* = 64); from *I*. *leithii* by mean genetic divergence of 4.7 ± 0.7% (range 3.9–5.8%, *N* = 20); from *I*. *sarojamma* by mean genetic divergence of 5.1 ± 0.4% (range 4.9–5.7%, *N* = 5); from *I*. *tysoni* by mean genetic divergence of 3.1 ± 0.1% (range 3.0–3.1%, *N* = 9); and from *I*. *yadera* by mean genetic divergence of 5.6 ± 0.2% (range 5.3–6.1%, *N* = 12) (S3 and S4 Tables). See *I*. *beddomii* for comparison with that species.

Description of holotype (measurements in mm). Adult female (SVL 38.7); head small, its length (HL 14.9) subequal to the width (HW 15.0), flat above; snout nearly pointed in dorsal view, nearly acute in lateral view, its length (SL 6.4) longer than horizontal diameter of eye (EL 4.5); loreal region acute and concave with rounded canthus rostralis; interorbital space flat, wider (IUE 3.4) than upper eyelid (UEW 3.0), and narrower than internarial distance (IN 4.1); nostrils closer to tip of snout (NS 2.7) than eye (EN 3.7); tympanum (TYD 2.8) 62% of eye diameter (EL 4.5). Forelimbs (FAL 7.9) shorter than hand length (HAL 11.2), finger length formula: IV<I<II<III, finger discs moderately wide compared to finger width (FD_I_ 1.1, FW_I_ 0.6; FD_II_ 0.9, FW_II_ 0.5; FD_III_ 1.4, FW_III_ 0.6; FD_IV_ 1.4, FW_IV_ 0.5). Thigh length (TL 22.5) shorter than shank (SHL 25.0), and longer than foot (FOL 21.9), toe discs wide compared to toe width (TD_I_ 1.4, TW_I_ 0.6; TD_II_ 1.7, TW_II_ 0.5; TD_III_ 1.7, TW_III_ 0.6; TD_IV_ 1.7, TW_IV_ 0.6; TD_V_ 1.2, TW_V_ 0.5), foot webbing: I1–2II1–2^1^/_4_III1–3^−^IV3^−^–1V (Fig 5).

Skin of snout and between eyes shagreened, upper eyelids shagreened to sparsely granular; posterior part of dorsum sparsely granular; dorsum with a few longitudinal discontinuous folds, lateral surface granular; anterior and posterior parts of flanks shagreened to sparsely granular; dorsal surface of forelimbs shagreened to finely granular. Ventral surfaces of throat and chest smooth, abdomen and posterior parts of thigh granular.

Colour of holotype. In life. Dorsum light brown with irregular dark brown blotches especially along the dorsal skin folds; a dark greyish-brown band between the eyes which continues over the upper eyelid; snout lighter in colour than dorsum; margins of upper and lower jaw with alternate dark brown and cream coloured cross-bars (Fig 6); a dark blackish-brown band extending from the from tip of snout through the lower margin of eye, widening behind the eye and over the tympanum, and ending near the armpit on either sides of the head; tympanum dark greyish-brown; forelimbs (including fingers) and hindlimbs (including toes) light brown with dark brown transverse bands; anterior and posterior parts of flank light yellowish-brown. Ventral surface light grey with dark grey spots. In preservation. Dorsum brown with irregular dark-brown blotches, especially along with dorsal skin folds; snout light brown; margins of upper and lower jaw with alternate dark brown and cream coloured cross-bars (Fig 7); a dark brown band extending from the snout through the lower margin of eye, widening behind the eye and over the tympanum, and ending near the armpit on either sides of the head; tympanum reddish-brown; forelimbs and hindlimbs brown with dark brown cross-bands. Ventral surfaces light creamish-brown (Fig 7).

Variation. See Table 1 for morphometric data from an adult male and female.

Distribution and natural history. *Indirana bhadrai* is currently known only from its type locality Muthodi forest, Bhadra Wildlife Sanctuary, located north of Palghat Gap in the Western Ghats state of Karnataka (Fig 3). Animals were found on leaf litter in a secondary forest, between 18:00–19:00 hours.

**Table 1:** Measurements (in mm) of the type and referred specimens of two new species of *Indirana*. The range, mean value, and standard deviation are given for each parameter.

	*Indirana bhadrai* sp. nov.		*Indirana paramakri* sp. nov.	
	Male (*N* = 1)	Female (*N* = 1)	Male (*N* = 2)	Female (*N* = 2)
SVL	30.2	38.7	27.4–30.1 (28.8 ± 1.9)	32.9–34.1 (33.5 ± 0.8)
HW	11.8	15.0	11.3–11.7 (11.5 ± 0.3)	13.0–13.1 (13.1 ± 0.1)
HL	11.5	14.9	12.0–12.3 (12.2 ± 0.2)	13.6–13.8 (13.7 ± 0.1)
TYD	2.4	2.8	2.8–3.5 (3.2 ± 0.5)	3.1–3.2 (3.2 ± 0.1)
SL	5.4	6.4	4.5–5.2 (4.9 ± 0.5)	5.6–5.8 (5.7 ± 0.1)
EL	3.9	4.5	3.4–3.8 (3.6 ± 0.3)	3.9–4.1 (4.0 ± 0.1)
MN	10.2	11.6	9.1–9.6 (9.4 ± 0.4)	11.4–11.7 (11.6 ± 0.2)
EN	2.3	3.7	2.3–2.6 (2.5 ± 0.2)	2.4–3.1 (2.8 ± 0.5)
NS	1.9	2.7	1.5–1.6 (1.6 ± 0.1)	1.8–2.2 (2.0 ± 0.3)
IUE	3.1	3.4	2.8–3.2 (3.0 ± 0.3)	3.2–3.4 (3.3 ± 0.1)
UEW	2.1	3.0	1.9–2.1 (2.0 ± 0.1)	2.3–2.7 (2.5 ± 0.3)
FAL	6.7	7.9	5.4–6.6 (6.0 ± 0.8)	6.6–6.8 (6.7 ± 0.1)
HAL	8.1	11.2	6.6–7.9 (7.3 ± 0.9)	7.4–7.8 (7.6 ± 0.3)
FIL	5.1	6.1	3.7–3.8 (3.8 ± 0.1)	3.8–4.1 (4.0 ± 0.2)
FIIL	5.2	6.2	3.7–3.7 (3.7 ± 0.0)	3.7–4.0 (3.9 ± 0.2)
FIIIL	6.2	7.3	3.5–4.3 (3.9 ± 0.6)	4.3–5.2 (4.8 ± 0.6)
FIVL	4.1	5.3	2.9–3.0 (3.0 ± 0.1)	3.3–3.4 (3.4 ± 0.1)
TL	18.3	22.5	14.9–16.8 (15.9 ± 1.3)	16.4–16.7 (16.6 ± 0.2)
ShL	20.1	25.0	15.9–17.3 (16.6 ± 1.0)	17.9–18.6 (18.3 ± 0.5)
FOL	18.9	21.9	14.2–15.6 (14.9±1.0)	16.2–16.8 (16.5 ± 0.4)

***Indirana brachytarsus*** (Günther 1876)

Short-legged Leaping Frog [58]

(Figs 1–6; S1–S4 Figs; S1–S4 Tables; S1 File)

Original name and description. *Polypedates brachytarsus* Günther 1876. Third report on collections of Indian reptiles obtained by the British Museum, *Proceedings of the Zoological Society of London*, 1876 “1875”: 572. Lectotype. NHM 74.4.29.1307 (ex BMNH 1947.2.27.92), designated by Inger *et al*. [59], adult female, from “Anamallays”. Type locality. “Anamallays”, India, according to original description. Current status of specific name. Valid name, as *Indirana brachytarsus* (Günther 1876).

Comments. Originally described by Günther [6] as *Polypedates brachytarsus* from Anamallays and Sevagherry, this species was placed under the synonymy of *Rana beddomii* [7], and later resurrected as a distinct species [59]. Inger *et al*. [59] also designated NHM 74.4.29.1307 (ex BMNH 1947.2.27.92) (from Anamallays) as the lectotype of *Polypedates brachytarsus* (S4 Fig), but cited the voucher number as “BMNH 1947.2.27.1307”. In order to avoid confusion, we confirm the voucher number of the lectotype to be NHM 74.4.29.1307 (ex BMNH 1947.2.27.92) as per the bottle label and the NHM catalogue.

Genetic divergence. Intraspecific genetic variation within populations of *I*. *brachytarsus* is 0.4 ± 0.3% (range 0–1.5%, *N* = 64) for 16S mitochondrial gene sequences. *Indirana brachytarsus* is closely related to members of the *I*. *beddomii* group (Figs 1 and 2); from *I*. *leithii* by mean genetic divergence of 6.7 ± 0.6% (range 5.6–7.9%, *N* = 1280); from *I*. *sarojamma* by mean genetic divergence of 8.0 ± 0.4% (range 7.5–9.0%, *N* = 320); differs from *I*. *tysoni* by mean genetic divergence of 6.9 ± 0.2% (range 6.4–7.7%, *N* = 576); and from *I*. *yadera* by mean genetic divergence of 8.7 ± 0.3% (range 8.1–9.6%, *N* = 768) (S3 and S4 Tables). See *I*. *beddomii* and *I*. *bhadrai* for comparison with those species.

Distribution and natural history. *Indirana brachytarsus* is one of the most commonly occurring *Indirana* species in the Western Ghats states of Kerala and Tamil Nadu, but its distribution is restricted to the south of Palghat gap (Fig 3 and S1 Table). In our study, we found the northernmost occurrence of this species at Kaikatti (Nelliyampathy) in Palakkad district of Kerala, and at Andiparai shola (Valparai) in Coimbatore district of Tamil Nadu. In the present study, specimens were located mostly during the night (between 18:00–23:00 hours) either on the surface of wet rocks or on leaf litter near streams in moist deciduous to wet evergreen and semi-evergreen tropical forests, at elevations between 130–1508 m asl. For details of the distribution records reported in this study see S1 Table, and for other genetically confirmed records see S2 Table.

For morphological comparison, description of lectotype, secondary sexual characters, and list of specimens examined, see S1 File.

***Indirana leithii*** (Boulenger 1888)

Matheran Leaping Frog [12]

(Figs 1–6; S2, S3 and S5 Figs; S1–S4 Tables; S1 File)

Original name and description. *Rana leithii* Boulenger 1888. Description of two new Indian species of *Rana*. *Annals and Magazine of Natural History*, *Series 6*, 1888(2): 506. Holotype. NHM 1947.2.28.17 (ex BMNH 1869.8.28.50), “single (female) specimen, presented by Dr. Leith”, by original designation. Type locality. “Matheran, Bombay”, India. Current status of specific name. Valid name, as *Indirana leithii* (Boulenger 1888).

Genetic divergence. Intraspecific genetic variation within populations of *I*. *leithii* is 0.7 ± 0.7% (range 0–1.7%, *N* = 20) for 16S mitochondrial gene sequences. *I*. *leithii* is closely related to members of the *Indirana beddomii* group (Figs 1 and 2); differs from *I*. *sarojamma* by mean genetic divergence of 6.2 ± 0.6% (range 5.5–7.3%, *N* = 100); from *I*. *tysoni* by mean genetic divergence of 6.1 ± 0.7% (range 5.2–7.1%, *N* = 180); and from *I*. *yadera* by mean genetic divergence of 7.0 ± 0.7% (range 6.1–8.2%, *N* = 240) (S3 and S4 Tables). See *I*. *beddomii*, *I*. *bhadrai* and *I*. *brachytarsus* for comparison with those species.

Distribution and natural history. The distribution of *Indirana leithii* is restricted to the state of Maharashtra, north of Goa gap (Fig 3 and S1 Table). In this study, we recorded this species at Bhimashankar (Pune district), Matheran (Raigad district), and Mahabaleshwar (Satara district). Specimens were located mostly during night searches (between 17:00 to 22:00 hours) either on wet laterite soil in a secondary forest patch (Matheran) or on wet leaf litter (Bhimashankar, Mahabaleshwar). For details of the distribution records reported in this study see S1 Table, and for other genetically confirmed records see S2 Table.

For morphological comparison and list of specimens examined, see S1 File.

***Indirana sarojamma*** Dahanukar, Modak, Krutha, Nameer, Padhye and Molur 2016

Sarojamma’s Leaping Frog [12]

(Figs 1–6; S2, S3 and S5 Figs; S1–S4 Tables; S1 File)

Original name and description. *Indirana sarojamma* Dahanukar, Modak, Krutha, Nameer, Padhye and Molur 2016. Leaping frogs (Anura: Ranixalidae) of the Western Ghats of India: An integrated taxonomic review. *Journal of Threatened Taxa*, 2016(8): 9278–9280. Holotype. BNHS 5981, female, by original designation. Type locality. “Ponmudi Reserve Forest (8.736°N & 77.141°E, elevation 879m), Kerala, India”. Current status of specific name. Valid name, as *Indirana sarojamma* Dahanukar, Modak, Krutha, Nameer, Padhye and Molur 2016.

Genetic divergence. Intraspecific genetic variation within populations of *I*. *sarojamma* was 0.2 ± 0.2% (range 0–0.4%, *N* = 5) for 16S mitochondrial gene sequences. *I*. *sarojamma* is closely related to members of the *Indirana beddomii* group (Figs 1 and 2); differs from *I*. *tysoni* by mean genetic divergence of 5.8 ± 0.2% (range 5.6–6.1%, *N* = 45); and from *I*. *yadera* by mean genetic divergence of 3.5 ± 0.1% (range 3.3–3.7%, *N* = 60) (S3 and S4 Tables). For comparison with *I*. *beddomii*, *I*. *bhadrai*, *I*. *brachytarsus* and *I*. *leithii* see ‘Genetic divergence’ of those species.

Distribution and natural history. *Indirana sarojamma* is currently known only from a small geographic region, south of Palghat gap in the Thiruvananthapuram district of Kerala state (Fig 3 and S1 Table). In the present study, we found this species on the surface of wet rocks close to primary forest at Chathankod–Bonnacaud (488 m asl), and under leaf litter on the floor of evergreen forest at Ponmudi (1014 m asl) in the Agasthyamala hills. Specimens were mostly located at night (between 18:00–21:00 hours). For details of the distribution records reported in this study see S1 Table.

For morphological comparison, variations, and list of specimens examined, see S1 File.

***Indirana tysoni*** Dahanukar, Modak, Krutha, Nameer, Padhye and Molur 2016

Tyson’s Leaping Frog [12]

(Figs 1–6; S2, S3 and S6 Figs; S1–S4 Tables; S1 File)

Original name and description. *Indirana tysoni* Dahanukar, Modak, Krutha, Nameer, Padhye and Molur 2016. Leaping frogs (Anura: Ranixalidae) of the Western Ghats of India: An integrated taxonomic review. *Journal of Threatened Taxa*, 2016(8): 9258–9259. Holotype. BNHS 5979, male, by original designation. Type locality. “Ranipuram Vested Forest (12.419°N & 75.353°E, elevation 932m)”, Kerala, India. Current status of specific name. Valid name, as *Indirana tysoni* Dahanukar, Modak, Krutha, Nameer, Padhye and Molur 2016.

Genetic divergence. Intraspecific genetic variation within populations of *I*. *tysoni* is zero (*N* = 9) for 16S mitochondrial gene sequences. *I*. *tysoni* is closely related to members of the *I*. *beddomii* group (Figs 1 and 2) and differs from *I*. *yadera* by mean genetic divergence of 5.9 ± 0.1% (range 5.6–6.1%, *N* = 108) (S3 and S4 Tables). For comparison with *I*. *beddomii*, *I*. *bhadrai*, *I*. *brachytarsus*, *I*. *leithii* and *I*. *sarojamma* see ‘Genetic divergence’ of those species.

Distribution and natural history. *Indirana tysoni* is currently known only from the Western Ghats states of Karnataka and Kerala, north of Palghat gap (Fig 3). In the present study, this species is reported from Karnataka: Chikmagalur district (Charmadi Ghats) and Kodagu district (Bhagamandala, Coorg, Madikeri and Thalakaveri). Specimens were located in secondary forest patches, mostly on surfaces of moss-covered rocks or moist leaf litter away from water-bodies during the late evening hours. During daytime (around 16:00 hours) they were found the under leaf litter. For details of the distribution records reported in this study see S1 Table, and for other genetically confirmed records see S2 Table.

For morphological comparison, secondary sexual characters, variations, and list of specimens examined, see S1 File.

***Indirana yadera*** Dahanukar, Modak, Krutha, Nameer, Padhye and Molur 2016

Yadera Leaping Frog [12]

(Figs 1–6; S2, S3 and S6 Figs; S1–S4 Tables; S1 File)

Original name and description. *Indirana yadera* Dahanukar, Modak, Krutha, Nameer, Padhye and Molur 2016. Leaping frogs (Anura: Ranixalidae) of the Western Ghats of India: An integrated taxonomic review. *Journal of Threatened Taxa*, 2016(8): 9280–9282. Holotype. BNHS 5982, female, by original designation. Type locality. “Vathikudy, Idukki Wildlife Sanctuary (9.874°N & 77.076°E, elevation 797m)”, Kerala, India. Current status of specific name. Valid name, as *Indirana yadera* Dahanukar, Modak, Krutha, Nameer, Padhye and Molur 2016.

Genetic divergence. Intraspecific genetic variation within populations of *I*. *yadera* is 0.4 ± 0.3% (range 0–1.1%, *N* = 12) for 16S mitochondrial gene sequences. *I*. *yadera* is closely related to members of the *Indirana beddomii* group (Figs 1 and 2; S3 and S4 Tables). For comparison with *I*. *beddomii*, *I*. *bhadrai*, *I*. *brachytarsus*, *I*. *leithii*, *I*. *sarojamma* and *I*. *tysoni* see ‘Genetic divergence’ of those species.

Distribution and natural history. *Indirana yadera* is currently known only from south of Palghat gap in the Western Ghats state of Kerala. In the present study, this species was collected from: Methooty in Idukki district, Neriamangalam in Ernakulam district, Pakuthipaalam (Nelliampathy) in Palakkad district, Kozhikana and Nilakkal in Pathanamthitta district, and Vazhachal in Thrissur district (Fig 3 and S1 Table). Specimens were mostly located during night searches (between 20:00–22:00 hours) on rock surfaces close to hill streams either in disturbed secondary forest patches (Methooty and Nelliyampathy) or undisturbed forests (Gavi and Vazhachal). For details of the distribution records reported in this study see S1 Table, and for other genetically confirmed records see S2 Table.

For morphological comparison, secondary sexual characters, variations, and list of specimens examined, see S1 File.

*Indirana semipalmata* group. Morphologically this group can be distinguished from *I*. *beddomii* group by the following suite of characters: small to medium-sized adult (male: SVL 23.0–43.0 mm, female: SVL 30.0–56.0 mm), first finger equal or nearly equal to second finger (Fig 4), tympanum well developed, large or nearly equal to horizontal diameter of eye (S2 Fig), fourth toe webbing extending up to or beyond the second subarticular tubercle on either side, except in *I*. *semipalamata* (Fig 5), and presence of prominent discontinuous longitudinal skin folds on dorsum.

Members included. *Indirana chiravasi*, *I*. *duboisi*, *I*. *gundia*, *Indirana paramakri* sp. nov., *I*. *salelkari* and *I*. *semipalmata*.

***Indirana chiravasi*** Padhye, Modak and Dahanukar 2014

Amboli Leaping Frog [10]

(Figs 1–5 and 8; S2, S3 and S7 Figs; S1–S4 Tables; S1 File)

Original name and description. *Indirana chiravasi* Padhye, Modak and Dahanukar 2014. *Indirana chiravasi*, a new species of leaping frog (Anura: Ranixalidae) from Western Ghats of India. *Journal of Threatened Taxa*, 2014(6): 6293–6312. Holotype. BNHS 5888, an adult male, by original designation. Type locality. “Amboli, Sindhudurg District, Maharashtra, India”. Current status of specific name. Valid name, as *Indirana chiravasi* Padhye, Modak and Dahanukar 2014.

Comment. We could not examine the type specimens of this species, as they were unavailable during our visits to the BNHS museum in September 2015 and February 2016.

Genetic divergence. Intraspecific genetic variation within populations of *Indirana chiravasi* is 0.1 ± 0.1% (range 0–0.4%, *N* = 17) for 16S mitochondrial gene sequences. *I*. *chiravasi* is closely related to members of the *Indirana semipalmata* group (Figs 1 and 2); differs from *I*. *duboisi* by mean genetic divergence of 3.0 ± 0.2% (range 2.7–3.7%, *N* = 323); from *I*. *gundia* by mean genetic divergence of 3.9 ± 0.3% (range 3.5–4.6%, *N* = 357); from *I*. *paramakri* by mean genetic divergence of 6.7 ± 0.5% (range 6.1–7.8%, *N* = 136); from *I*. *salelkari* by mean genetic divergence of 3.2 ± 0.4% (range 2.5–3.7%, *N* = 102); and from *I*. *semipalmata* by mean genetic divergence of 5.1 ± 0.4% (range 4.3–6.4%, *N* = 646) (S3 and S4 Tables).

Distribution and natural history. *Indirana chiravasi* is presently known to occur only in the northern Western Ghats, Maharashtra state. In the present study, this species is reported from Amboli (Sindhudurg district), Phansad WLS (Raigad district) and Koyna (Satara district) of Maharashtra (Fig 3 and S1 Table). For details of the distribution records reported in this study see S1 Table, and for other genetically confirmed records see S2 Table.

For morphological comparison and list of specimens examined, see S1 File.

***Indirana duboisi*** Dahanukar, Modak, Krutha, Nameer, Padhye and Molur 2016

Dubois’s Leaping Frog [12]

(Figs 1–5 and 8; S2, S3 and S7 Figs; S1–S4 Tables; S1 File)

Original name and description. *Indirana duboisi* Dahanukar, Modak, Krutha, Nameer, Padhye and Molur 2016. Leaping frogs (Anura: Ranixalidae) of the Western Ghats of India: An integrated taxonomic review. *Journal of Threatened Taxa*, 2016(8): 9272–9274. Holotype. BNHS 5980, female, by original designation. Type locality. Kerekatte, Kudremukh National Park (13.322°N & 75.146°E, elevation 724m)”, Karnataka, India. Current status of specific name. Valid name, as *Indirana duboisi* Dahanukar, Modak, Krutha, Nameer, Padhye and Molur 2016.

Genetic divergence. Intraspecific genetic variation within populations of *Indirana duboisi* is 0.5 ± 0.3% (range 0–1.3%, *N* = 19) for 16S mitochondrial gene sequences. *I*. *duboisi* is closely related to members of the *Indirana semipalmata* group (Figs 1 and 2); differs from *I*. *gundia* by mean genetic divergence of 3.2 ± 0.3% (range 2.4–3.9%, *N* = 399); from *I*. *paramakri* by mean genetic divergence of 5.4 ± 0.6% (range 4.5–7.0%, *N* = 152); from *I*. *salelkari* by mean genetic divergence of 2.6 ± 0.3% (range 2.1–3.3%, *N* = 114); and from *I*. *semipalmata* by mean genetic divergence of 4.7 ± 0.3% (range 4.2–5.6%, *N* = 722) (S3 and S4 Tables). See *I*. *chiravasi* for comparison with that species.

Distribution and natural history. *Indirana duboisi* is currently known only from the Western Ghats state of Karnataka, north of Palghat gap. In this study, we report the presence of this species in Chikmagalur district (Bygoor and Charmadi Ghats), Dakshin Kannada district (Gundia–Kempholey), Hassan district (Kempholey and Kottigehara), Shimoga district (Agumbe), and Uttara Kannada district (Castle rock and Kathlekan) (Fig 3 and S1 Table). Specimens were mostly found on forest floor, either on surface of wet rocks or on leaf litter, inside secondary forests. For details of the distribution records reported in this study see S1 Table, and for other genetically confirmed records see S2 Table.

For morphological comparison and list of specimens examined, see S1 File.

***Indirana gundia*** (Dubois 1986)

Gundia Leaping Frog [12]

(Figs 1–5 and 8; S1–S3 and S8 Figs; S1–S4 Tables; S1 File)

Original name and description. *Ranixalus gundia* Dubois 1986. Diagnose préliminaire d’un nouveau genre de Ranoidea (Amphibiens, Anoures) du sud de l’Inde. *Alytes*, 1986(4): 114–118. Holotype. MNHNP 1985.633, female, by original designation. Type locality. “Gundia, forêt de Kemphole, à l'ouest de Sakleshpur, Karnataka, Inde”. Current status of specific name. Valid name, as *Indirana gundia* (Dubois 1986).

Genetic divergence. Intraspecific genetic variation within populations of *Indirana gundia* is 0.2 ± 0.2% (range 0–0.7%, *N* = 21) for 16S mitochondrial gene sequences. *Indirana gundia* is closely related to members of the *Indirana semipalmata* group (Figs 1 and 2); differs from *I*. *paramakri* by mean genetic divergence of 5.2 ± 0.5% (range 4.2–6.3%, *N* = 168); from *I*. *salelkari* by mean genetic divergence of 4.0 ± 0.3% (range 3.5–4.4%, *N* = 126); and from *I*. *semipalmata* by mean genetic divergence of 3.3 ± 0.2% (range 2.6–3.9%, *N* = 798) (S3 and S4 Tables). See *I*. *chiravasi* and *I*. *duboisi* for comparison with those species.

Distribution and natural history. *Indirana gundia* is currently known only from the Western Ghats states of Karnataka and Kerala, north of Palghat gap. In this study, we report the presence of this species at Gundia (Dakshin Kannada district), Kempholey (Hassan district), Monnangeri (Kodagu district) and Kudremukh (Udupi district) in Karnataka state; and Aralam (Kannur district) in Kerala state. The distribution of this species is restricted to the north of Palghat gap (Fig 3 and S1 Table). Animals were located both during day and night searches, mostly on forest floor covered with leaf litter or on rock surfaces near forest streams. For details of the distribution records reported in this study see S1 Table, and for other genetically confirmed records see S2 Table.

For morphological comparison, secondary sexual characters, and list of specimens examined, see S1 File.

***Indirana paramakri*** sp. nov.

urn:lsid:zoobank.org:act:AFE5AB9D-9B57-4C01-A9A5-AD3D73FA3399

**Fig 8:**
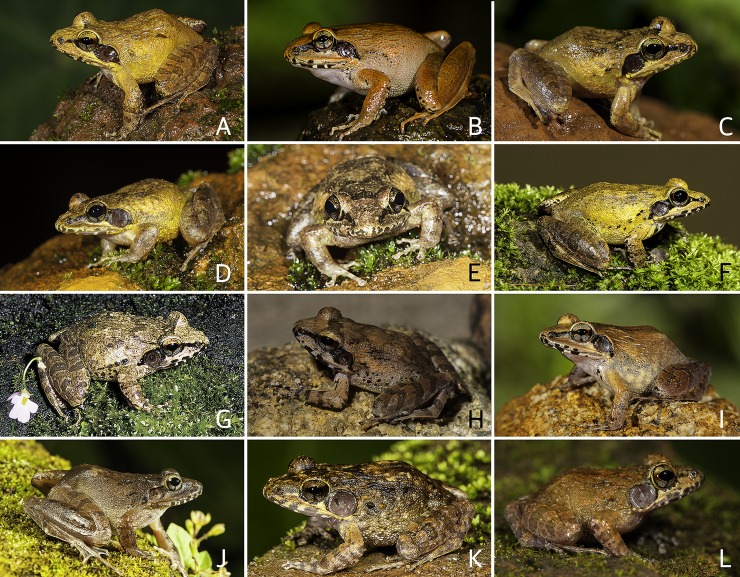
*Indirana semipalmata* group in life. (A–C) *I*. *chiravasi*. (A) An adult male (not preserved), from Amboli. (B) An adult female (SDBDU 2012.2124), from Amboli. (C) An adult male (SDBDU 2012.2125), from Amboli. (D–F) *I*. *duboisi*. (D, E) An adult male (SDBDU 2014.2517), from Agumbe. (D) Dorsolateral view. (E) Frontal view. (F) An adult male (SDBDU 2011.1399), from Charmadi Ghats. (G–H) *I*. *gundia*. (G) An adult male (SDBDU 2008.433), from Kempholey. (H) An adult male (SDBDU 2002.1115), from Kempholey. (I) *Indirana paramakri* sp. nov., holotype, an adult female (ZSI/WGRC/V/A888), from Suganthagiri. (J) *I*. *salelkari*, an adult female (SDBDU 2011.1330), from Dandeli, dorsolateral view. (K–L) *I*. *semipalmata*. (K) An adult female (SDBDU 2015.3033), from Parambikulam. (L) An adult male (SDBDU 2015.3014), from Singappara.

Rocky-terrain Leaping Frog

(Figs 1–5, 7 and 8; Table 1; S2 and S3 Figs; S1–S4 Tables)

Etymology. The species epithet ‘paramakri’ is derived from two Malayalam (the official language of Kerala state) words–*para* meaning ‘rock’ and *makri* for ‘frog’–referring to the predominant occurrence of Leaping frog species in rocky terrains. The species name is a noun standing in apposition to the generic name, and therefore invariable.

Holotype. ZSI/WGRC/V/A888, an adult female, from Suganthagiri (11.5386°N 76.0539°E, 852 m asl), Wayanad district, Kerala state, collected by SDB on 15 October 2005.

Paratypes. ZSI/WGRC/V/A889, an adult female, from Settukunnu (11.6172°N 75.9913°E, 823 m asl), Wayanad district, Kerala state, collected by SDB and SG on 5 June 2015; ZSI/WGRC/V/A890, an adult male, also from Settukunnu, Wayanad district, Kerala state, collected by SDB 20 July 2003.

Referred specimen. SDBDU 2005.3741, an adult male, from Suganthagiri (11.5386°N 76.0539°E, 852 m asl), Wayanad district, Kerala state, collected along with the holotype.

Comparison. Based on the overall morphology, *Indirana paramakri* sp. nov. could be confused with members of the *Indirana semipalmata* group. However, *I*. *paramakri* differs from *I*. *chiravasi*, *I*. *duboisi*, *I*. *gundia*, *I*. *salelkari* and *I*. *semipalmata* by its loreal region acute (vs. obtuse in all five species); specifically differs from *I*. *duboisi*, *I*. *gundia*, *I*. *salelkari* and *I*. *semipalmata* by its snout sub-ovoid in dorsal view (vs. nearly pointed in *I*. *duboisi*; rounded in *I*. *gundia*, *I*. *salelkari* and *I*. *semipalmata*); differs from *I*. *chiravasi*, *I*. *duboisi*, *I*. *gundia* and *I*. *salelkari* by its webbing between first, second and third toe well below the disc on the outside, I1^2/3^–2^+^II1^3/4^–2^3/4^III2^−^–3IV3–1^+^V (vs. extending up to the disc in all four species) (Fig 5); specifically differs from *I*. *semipalmata* by its snout sub-ovoid in dorsal view (vs. rounded), fourth toe webbing extending up to the second subarticular tubercle on either side, I1^2/3^–2^+^II1^3/4^–2^3/4^III2^−^–3IV3–1^+^V (vs. below, I2^−^–2^+^II2^−^–3^−^III2–3^1/4^IV3^1/2^–2V), and fifth toe webbing extending well above the first subarticular tubercle, I1^2/3^–2^+^II1^3/4^–2^3/4^III2^−^–3IV3–1^+^V (vs. up to the first subarticular tubercle, I2^−^–2^+^II2^−^–3^−^III2–3^1/4^IV3^1/2^–2V) (Fig 5).

For better clarity, we compare this new species with all other currently known species in the genus. *Indirana wayanadi* differs from all the members of *Indirana beddomii* group by its webbing between first, second and third toe well below the disc on the outside, I1^2/3^–2^+^II1^3/4^–2^3/4^III2^−^–3IV3–1^+^V (vs. extending up to the disc in all species) (Fig 5), and relatively smaller snout-vent size (except *I*. *leithii*), male SVL 27.4–30.1 mm, *N* = 2, female SVL 32.9–34.1 mm, *N* = 2 (vs. larger, *I*. *beddomii* male SVL 28.2–35.2 mm, *N* = 2, female SVL 44.8–55.5 mm, *N* = 5; *I*. *bhadrai*, male SVL 30.2 mm, *N* = 1, female SVL 38.7 mm, *N* = 1; *I*. *brachytarsus*, male SVL 30.6–34.3 mm, *N* = 3, female SVL 37.5–45.2 mm, *N* = 6; *I*. *sarojamma*, male SVL 34.5–38.2 mm, *N* = 2, female SVL 61.2 mm, *N* = 1; *I*. *tysoni*, male SVL 31.7–33.9 mm, *N* = 4, female SVL 51.3–52.8 mm, *N* = 2; *I*. *yadera*, male SVL 40.1–45.5 mm, *N* = 3, female SVL 58.6 mm, *N* = 1). More specifically differs from *I*. *beddomii*, *I*. *leithii*, *I*. *sarojamma*, *I*. *tysoni* and *I*. *yadera* by its first finger equal or nearly equal to second finger (vs. shorter in *I*. *leithii*, and longer in other four species) (Fig 4).

Furthermore, *Indirana paramakri* differs from members of the genus *Sallywalkerana* by its first finger equal or nearly equal to second finger (vs. shorter in all members) and fourth toe webbing extending up to the second subarticular tubercle on either side, I1^2/3^–2^+^II1^3/4^–2^3/4^III2^−^–3IV3–1^+^V (vs. well below in all members) (Fig 5).

Genetic divergence. Intraspecific genetic variation within populations of *Indirana paramakri* sp. nov. is 0.6 ± 0.4% (range 0–1.3%, *N* = 8) for 16S mitochondrial gene sequences. *Indirana paramakri* is closely related to members of the *Indirana semipalmata* group (Figs 1 and 2); differs from *I*. *salelkari* by mean genetic divergence of 6.1 ± 0.5% (range 5.5–7.0%, *N* = 48); and from *I*. *semipalmata* by mean genetic divergence of 4.5 ± 0.5% (range 3.7–5.9%, *N* = 304) (S3 and S4 Tables). For comparison with *I*. *chiravasi*, *I*. *duboisi* and *I*. *gundia* see ‘Genetic divergence’ of those species.

Description of holotype (measurements in mm). Adult female (SVL 32.9); head small, longer than wide (HW 13.1, HL 13.8), flat above; snout sub-ovoid in dorsal view, rounded in lateral view, its length (SL 5.6) longer than horizontal diameter of eye (EL 4.1); loreal region acute and concave with rounded canthus rostralis; interorbital space flat, wider (IUE 3.4) than upper eyelid (UEW 2.7) and equal to internarial distance (IN 3.4); nostril closer to tip of snout (NS 1.8) than eye (EN 3.1); tympanum (TYD 3.1) 76% of eye diameter (EL 4.1). Forelimbs (FAL 6.6) shorter than hand length (HAL 7.8), finger length formula: IV<II<I<III, finger discs moderately wide compared to finger width FD_I_ 0.8, FW_I_ 0.5; FD_II_ 0.8, FW_II_ 0.6; FD_III_ 1.0, FW_III_ 0.7; FD_IV_1.0, FW_IV_ 0.5). Thigh length (TL 16.7) shorter than shank (SHL 17.9), and longer than foot (FOL 16.2), toe discs wide compared to toe width (TD_I_ 0.9, TW_I_ 0.4; TD_II_ 1.0, TW_II_ 0.4; TD_III_ 1.2, TW_III_ 0.5; TD_IV_ 1.3, TW_IV_ 0.6; TD_V_ 0.9, TW_V_ 0.4), foot webbing: I1^2/3^–2^+^II1^3/4^–2^3/4^III2^−^–3IV3–1^+^V.

Skin of snout and between eyes shagreened, upper eyelids shagreened to sparsely granular; posterior part of dorsum sparsely granular; dorsum with a few discontinuous longitudinal folds; lateral sides of head shagreened; anterior and posterior parts of flanks shagreened to sparsely granular; dorsal surface of forelimbs shagreened to finely granular; thigh, tibia and tarsus with weakly developed granular projections. Ventral surface of throat and chest smooth, abdomen and posterior parts of thigh prominently granular.

Colour of holotype. In life. Dorsum reddish-brown (Fig 8); a dark blackish-brown band extending from the nostril through the lower margin of eye, widening behind the eye and over the tympanum, and ending near the armpit on either sides of the head; tympanum blackish-brown, space between tympanum and eye dark blackish-brown; margins of upper and lower jaw with alternate dark brown and cream coloured cross-bars (Fig 8); forelimbs (including fingers) and hind limbs (including toes) reddish-brown with brown transverse bands; anterior and posterior parts of flanks light greyish-brown. Ventral surface light grey with a few scattered blackish-brown spots. In preservation. Dorsum greyish-brown with minute brown speckles; margins of upper and lower jaw with alternate dark brown and cream coloured cross-bars; a brown band extending from the nostril through the lower margin of eye, widening behind the eye and over the tympanum, and ending near the armpit on either sides of the head; tympanum brown; forelimbs and hindlimbs brown with dark brown transverse bands. Ventral surfaces light brown with a few scattered greyish-brown spot on throat and chest; ventral surface of limbs light greyish-brown, margins mottled with dark brown (Fig 7).

Secondary sexual characters. *Male* (ZSI/WGRC/V/A890), femoral glands present, nuptial pads present; *female* (ZSI/WGRC/V/A888), large pigmented eggs (diameter 0.5–0.8 mm, *N* = 20).

Variation. See Table 1 for morphometric data from two adult males and two adult females.

Distribution and natural history. *Indirana paramakri* is currently known only from two localities (Settukunnu and Suganthagiri), north of Palghat gap, in Wayanad district of the Western Ghats state of Kerala (Fig 3 and S1 Table). Animals were found in disturbed forest areas, either on surfaces of wet rocks near streams (Settukunnu) or under leaf litter and vegetation adjacent to a seasonal pond (Suganthagiri). Collections were made between 18:00–21:00 hours.

***Indirana salelkari*** Modak, Dahanukar, Gosavi and Padhye 2015

Netravali Leaping Frog [11]

(Figs 1–5 and 8; S2, S3 and S8 Figs; S1–S4 Tables; S1 File)

Original name and description. *Indirana salelkari* Modak, Dahanukar, Gosavi and Padhye 2015. *Indirana salelkari*, a new species of leaping frog (Anura: Ranixalidae) from Western Ghats of Goa, India. *Journal of Threatened Taxa*, 2015(7): 7493–7509. Holotype. BNHS 5931, male, by original designation. Type locality. “Tanshikar Spice Farm in Neturlim (15.095°N & 74.211°E; elevation 78m), Sanguem Taluk, South Goa District, Goa, India”. Current status of specific name. Valid name, as *Indirana salelkari* Modak, Dahanukar, Gosavi and Padhye 2015.

Comment. We could not examine the type specimens of this species, as they were unavailable during our visits to the BNHS museum in September 2015 and February 2016.

Genetic divergence. Intraspecific genetic variation within populations of *Indirana salelkari* is 1.2 ± 0.9% (range 0–2.2%, *N* = 6) for 16S mitochondrial gene sequences. *Indirana salelkari* is closely related to members of the *Indirana semipalmata* group (Figs 1 and 2) and differs from *I*. *semipalmata* by mean genetic divergence of 5.4 ± 0.5% (range 4.3–6.3%, *N* = 228) (S3 and S4 Tables). See *I*. *chiravasi*, *I*. *duboisi*, *I*. *gundia* and *I*. *paramakri* for comparison with those species.

Distribution and natural history. *Indirana salelkari* is currently known only from the Western Ghats states of Goa and Karnataka, north of Palghat gap. The present study reports the occurrence of this species in Uttara Kannada district (Dandeli and Unchali falls) and Shimoga district (Jog falls) of Karnataka (Fig 3 and S1 Table). The presence of *I*. *salelkari* at Unchali falls and Jog falls was only confirmed genetically, as the specimen from Unchali falls was a sub-adult while only a tissue sample (without specimen) was collected from Jog falls. We observed this species on leaf litter or rock cuttings adjacent to streams inside secondary forests. For details of the distribution records reported in this study see S1 Table, and for other genetically confirmed records see S2 Table.

For morphological comparison and specimen examined see S1 File.

***Indirana semipalmata*** (Boulenger 1882)

Brown Leaping Frog [58]

(Figs 1–5 and 8; S1–S3 and S8 Figs; S1–S4 Tables; S1 File)

Original name and description. *Rana semipalmata* Boulenger 1882. Catalogue of the Batrachia Salientia s. Ecaudata in the Collection of the British Museum. Second Edition. London: Taylor and Francis, 1882: 56–57. Lectotype. NHM 74.4.29.605 (ex BNHS 1947.2.29.50), designated by Dahanukar *et al*. [12], an adult female, from “Malabar”, figured in Boulenger 1882: Plate IV. Fig 3. Type locality. “Malabar”, India, according to original description. Current status of specific name. Valid name, as *Indirana semipalmata* (Boulenger 1882).

Comments. The original description [7] mentions two syntypes from Col. Beddome’s collection from “Malabar”. We examined the syntypes (a male and a female) at NHM, and found NHM 74.4.29.605 (ex BNHS 1947.2.29.50), an adult female, to be in agreement with the original description, with respect to snout-vent size (“From snout to vent 36 millim”), tympanum size (“tympanum distinct, about as large as the eye”) and webbing on foot (“toes half-webbed”). This specimen was also found to be in well-preserved condition (S8 Fig). Dahanukar *et al*. [12] designated NHM 74.4.29.605 (ex BNHS 1947.2.29.50) as the lectotype.

Genetic divergence. Intraspecific genetic variation within populations of *Indirana semipalmata* is 1.1 ± 0.7% (range 0–2.4%, *N* = 38) for 16S mitochondrial gene sequences. *Indirana semipalmata* is closely related to members of the *Indirana semipalmata* group (Figs 1 and 2; S3 and S4 Tables). See *I*. *chiravasi*, *I*. *duboisi*, *I*. *gundia*, *I*. *paramakri* and *I*. *salelkari* for comparison with those species.

Distribution and natural history. The distribution of *Indirana semipalmata* is restricted to the Western Ghats states of Kerala and Tamil Nadu. In the present study, this species is reported from Kerala: Ernakulam district (Neriamangalam), Idukki district (Kattapana, Kulamav, Pampadumpara and Periyar TR), Palakkad district (Nelliyampathi, Nenmara, Parambikulam TR and Siruvani), Pathanamthitta district (Gavi), and Thiruvananthapuram district (Chathankod–Bonnacaud and Kallar); Tamil Nadu: Top Slip, Karian Shola (Coimbatore district) (Fig 3 and S1 Table). Specimens were located during both day and night searches, and usually found on wet rocks close to streams or under leaf litter in primary and secondary forests. For details of the distribution records reported in this study see S1 Table, and for other genetically confirmed records see S2 Table.

For morphological comparison, description of lectotype, secondary sexual characters, variations, and list of specimens examined, see S1 File.

Genus. *Sallywalkerana* Dahanukar, Modak, Krutha, Nameer, Padhye and Molur 2016

Type species. *Ixalus diplostictus* Günther 1876 [= *Sallywalkerana diplosticta* (Günther 1876)]

Species included. *Sallywalkerana diplosticta* (Günther 1876), *S*. *leptodactyla* (Boulenger 1882), and *S*. *phrynoderma* (Boulenger 1882).

Distribution. The geographical range is restricted to south of Palghat gap in the Western Ghats. Distribution extends from Athirimala (Thiruvananthapuram district) to Eravikulam (Idukki district) in Kerala, and from Kakkachi (Tirunelveli district) to Valparai (Coimbatore district) in Tamil Nadu. For details of distribution records reported in the present study see Fig 3 and S1 Table.

Salient morphological characters. Male SVL 22.0–34.0 mm, female SVL 27.0–39.0 mm; pupil oval; presence of discontinuous dorsal skin folds or prominent skin warts; presence of various sized blotches on the ventral surface; vomerine teeth present; tongue emarginated posteriorly with lingual papillae; tympanum well-developed, smaller than horizontal diameter of eye (S2 Fig); first finger shorter than second finger (Fig 4); tips of fingers and toes with discs having distinct dorsoterminal grooves; interdigital webbing absent between fingers; webbing on foot reduced, just above the last subarticluar tubercles on toes II–IV (Fig 5).

***Sallywalkerana diplosticta*** (Günther 1876)

Spotted Leaping Frog [58]

(Figs 1–5 and 9; S2, S3 and S9 Figs; S1–S4 Tables; S1 File)

Original name and description. *Ixalus diplostictus* Günther 1876. Third report on collections of Indian reptiles obtained by the British Museum, *Proceedings of the Zoological Society of London*, 1876 “1875”: 574. Lectotype. NHM 1874.4.29.1412 (ex BMNH 1947.2.2.21), designated by Dahanukar *et al*. [12], adult female, from “Malabar”, figured in Günther 1876: LXIII. Fig C. Type locality. “Malabar”, India, according to original description. Current status of specific name. Valid name, as *Sallywalkerana diplosticta* (Günther 1876).

Comments. The original description [6] mentions four syntypes from “Malabar” based on Col. Beddome’s collection. We examined the three adult syntypes (one female and two males) available at NHM, and found NHM 1874.4.29.1412 (ex BMNH 1947.2.2.21), an adult female, to be in agreement with the original description, with respect to webbing between toes (“with a very short web”) and markings (“symmetrical black spots on the sides—one in front of the axil, another on the middle of the side of the trunk, a third above the loin”). This specimen was also found to be in relatively well-preserved condition (S9 Fig) and was designated as the lectotype by Dahanukar *et al*. [12].

Genetic divergence. Intraspecific genetic variation within populations of *S*. *diplosticta* is 1.1 ± 0.6% (range 0–1.7%, *N* = 9) for 16S mitochondrial gene sequences. *S*. *diplosticta* differs from *S*. *leptodactyla* by mean genetic divergence of 11.2 ± 0.4% (range 10.0–12.7%, *N* = 144); and from *I*. *phrynoderma* by mean genetic divergence of 12.2 ± 0.5% (range 11.5–12.7%, *N* = 18) (S3 and S4 Tables).

Distribution and natural history. *Sallywalkerana diplosticta* is currently known from a small geographical region in Agasthyamala hills, south of Palghat gap in the Western Ghats state of Kerala. In this study, we report this species from Kollam district (Pandimotta, Shendurney WLS) and Thiruvananthapuram district (Athirimala, Ponmudi and Pandipath) (Fig 3). Specimens were located during the night (between 18:00 to 21:00 hours) on wet rocks in primary forest at Athirimala, under vegetation near earthen cuttings at Pandipath, or on forest floor in evergreen forests at Ponmudi and Pandimotta. For details of the distribution records reported in this study see S1 Table, and for other genetically confirmed records see S2 Table.

**Fig 9:**
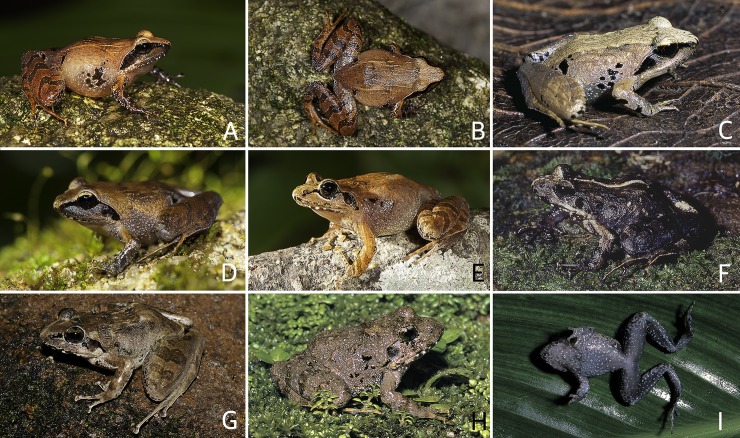
Genus *Sallywalkerana* in life. (A–D) *S*. *diplosticta*. (A, B) An adult male (SDBDU 2015.2956), from Pandipath. (A) Dorsolateral view. (B) Dorsal view. (C) An adult male (SDBDU 2003.40103), from Athirimala. (D) An adult male (SDBDU 2011.283), from Pandimotta. (E–G) *S*. *leptodactyla*. (E) An adult female (SDBDU 2013.911), from Mattupetti, dorsolateral view. (F) An adult female (SDBDU 2002.917), from Kodaikanal. (G) An adult female (SDBDU 2004.40336), from Valparai. (H, I) *S*. *phrynoderma*, an adult male (SDBDU 2002.1181), from Grass Hills. (H) Dorsolateral view. (I) Ventral view.

See S1 File for morphological comparison, description of lectotype, secondary sexual characters, and list of specimens examined, and Fig 9 for colouration in life.

***Sallywalkerana leptodactyla*** (Boulenger 1882)

Slender-toed Leaping Frog [12]

(Figs 1–5 and 9; S1–S3 and S9 Figs; S1–S4 Tables; S1 File)

Original name and description. *Polypedates brevipalmatus* Günther 1876 (Secondary homonym of *Rana brevipalmata* Peters, 1871). Third report on collections of Indian reptiles obtained by the British Museum, *Proceedings of the Zoological Society of London*, 1876 “1875”: 572–573. Lectotype. NHM 1874.4.29.593 (ex BMNH 1947.2.29.39), designated by Dahanukar *et al*. [12], adult female, from “Malabar”. Type locality. “Malabar”, India, according to original description. Current status of specific name. Valid name, as *Sallywalkerana leptodactyla* (Boulenger 1882).

Comments. The original description [6] mentions “several specimens” from “Malabar” and one specimen from “Anamallays”, based on Col. Beddome’s collection. We examined two adult syntypes (a female and a male) at NHM and found NHM 1874.4.29.593 (ex BMNH 1947.2.29.39), an adult female from “Malabar”, to be in agreement with the original description, with respect to webbing between toes (“toes long, with a very short web”), relative finger lengths (“Fingers without any web: the second rather longer than the first, and equal to the fourth, the third being the longest”), cross-band between the eyes (“a dark interocular cross-band”) and nearly similar SVL 35 mm (“Spec. B. 34 millim.”). This specimen was also found to be in relatively well-preserved condition (S9 Fig) and was designated as the lectotype by Dahanukar *et al*. [12].

Genetic divergence. Intraspecific genetic variation within populations of *S*. *leptodactyla* is 0.3 ± 0.3% (range 0–1.4%, *N* = 16) for 16S mitochondrial gene sequences. *S*. *leptodactyla* differs from *S*. *phrynoderma* by mean genetic divergence of 6.6 ± 0.3% (range 5.9–7.6%, N = 16) (S3 and S4 Tables). See *S*. *diplosticta* for comparison with that species.

Distribution and natural history. *Sallywalkerana leptodactyla* is currently known only from south of Palghat gap in the Western Ghats. In the present study, we report this species from Kerala: Idukki district (Eravikulam NP, Mattupetti and Munnar) and Thiruvananthapuram district (Ponkalapara) in Kerala; and Tamil Nadu: Coimbatore district (Grass hills and Valparai) and Dindigul district (Kodaikanal) (Fig 3 and S1 Table). Specimens were located during the night (between 16:00 to 20:00 hours), on surface of moss-covered rocks in open grasslands at Eravikulam NP, or under floor leaf litter in evergreen forest at Kodaikanal. For details of the distribution records reported in this study see S1 Table, and for other genetically confirmed records see S2 Table.

See S1 File for morphological comparison, description of lectotype, secondary sexual characters, variations, and list of specimens examined, and Fig 9 for colouration in life.

***Sallywalkerana phrynoderma*** (Boulenger 1882)

Warty-skinned Leaping Frog [12]

(Figs 1–5 and 9; S1–S3 and S9 Figs; S1–S4 Tables; S1 File)

Original name and description. *Rana phrynoderma* Boulenger 1882. Catalogue of the Batrachia Salientia s. Ecaudata in the Collection of the British Museum. Second Edition. London: Taylor and Francis, 1882: 462. Lectotype. NHM 82.2.10.21 (ex BMNH 1947.2.3.8), designated by Dahanukar *et al*. [12], female, from “Anamallays”. Type locality. “Anamallays”, India, according to original description. Current status of specific name. Valid name, as *Sallywalkerana phrynoderma* (Boulenger 1882).

Comments. The original description [7] mentions “*a-b*. Hgr.” from Anamallays based on Col. Beddome’s collection. We examined the two adult syntypes (a female and a male) at NHM, and found NHM 82.2.1021 (ex BMNH 1947.2.3.8), “Anamallays” to be in agreement with the original description, with respect to its adult size (“35 millim”) and dorsal skin texture (“upper parts covered with warts of different sizes and short glandular folds”). Dahanukar *et al*. [12] designated this specimen as the lectotype.

Genetic divergence. Intraspecific genetic variation among populations of *Sallywalkerana phrynoderma* was zero (*N* = 2) for 16S mitochondrial gene sequences. *S*. *phrynoderma* is closely related to *S*. *diplosticta* and *S*. *leptodactyla* (Figs 1 and 2; S4 Table). See *S*. *diplosticta* and *S*. *leptodactyla* for comparison with these species.

Distribution and natural history. *Sallywalkerana phrynoderma* is known from a small geographical region, south of Palghat gap in the Western Ghats. In the present study we report this species from Grass Hills (Akkamalai shola) in Coimbatore district of Tamil Nadu state (Fig 3 and S1 Table). Specimens were located during the day (around 11:00 hours) on moist leaf litter in a forest patch adjacent to open grassland. For details of the distribution records reported in this study see S1 Table, and for other genetically confirmed records see S2 Table.

See S1 File for morphological comparison, secondary sexual characters, and list of specimens examined, and Fig 9 for colouration in life.

## Discussion

While we formally describe two new species of Leaping frogs in the present work, our molecular data suggests that several other genetic lineages in family Ranixalidae (Fig 1) are likely to be complexes of cryptic species. For instance, a high degree of genetic differentiation is observed between various populations of *Indirana beddomii* (S3 Table). The distribution trend suggests genetic isolation between populations found north and south of the Palghat Gap, with up to 3.5% divergence for the mitochondrial 16S gene. A single DNA sequence of *I*. *beddomii* from Peruvannamuzhi (KX641761), situated north of Palghat gap, was found to nest with populations from south of Palghat gap, which could possibly be a locality error considering that other sequences (KX641759–60) from the same locality grouped with the northern populations (Fig 1). Two other species in our study were observed to have more than 2% intraspecific divergence (up to 2.2% for *I*. *salelkari*; up to 2.4% for *I*. *semipalmata*), which is comparable to the shallow interspecific genetic distances (minimum 2.1–2.7%) observed among some of the previously known members of the *Indirana semipalmata* group (e.g., minimum 2.1% between *I*. *duboisi* and *I salelkari*) (S4 Table). However, we found all these above-mentioned genetically divergent populations to be morphologically very similar to their closest relatives. Besides this, based on our preliminary genetic analysis one candidate species is suggested to be present in the genus *Indirana* (closely related to *I*. *tysoni*) and another in the genus *Sallywalkerana* (sister to *S*. *leptodactyla*). These populations could not be studied in the present work due to lack of voucher specimens or locality information for the DNA sequences found to be genetically divergent (JQ596673 and JQ596681–85, respectively), as well as sufficient samples in our collection. Nonetheless, the new and available genetic data clearly indicates the possible occurrence of undescribed cryptic ranixalid species. Considering the recent trends in description of numerous new anuran species from the Western Ghats, particularly in ancient and endemic lineages such as Micrixalidae [36] and Nyctibatrachidae [53], a high diversity of Leaping frogs is not unexpected but it is indicative that cryptic species remain undescribed even in recently and relatively well-studied groups. Detailed studies will be required to understand the observed intraspecific variations, particularly in *Indirana beddomii*, *I*. *salelkari*, *I*. *semipalmata*, *I*. *tysoni* and *Sallywalkerana leptodactyla*. Furthermore, genus *Sallywalkerana* was recognized largely based on reduced webbing, phylogenetic position and genetic divergence of >11% with all members of the genus *Indirana* [12]. According to our molecular analyses, high intraspecific genetic distances of >11% are even observed between *Sallywalkerana diplosticta* and populations of the other two *Sallywalkerana* species (with *S*. *leptodactyla*: mean 11.2%, range 10.0–12.7%; and with *S*. *phrynoderma*: mean 12.2%, range 11.5–12.7%) (S4 Table). Future integrated studies using multi-gene phylogenetic and phylogeographic analyses, bioacoustics as well as ecological data can provide better insights into the patterns of diversification and recognized taxonomic groupings in this ancient family.

Our findings also provide new insight into the distribution patterns of ranixalid species within the Western Ghats, while also reinforcing the role of elevational discontinuities (especially the 30 km wide Palghat gap) in determining distribution of anuran amphibians (e.g., [49, 52]). Members of the genus *Sallywalkerana* (*S*. *diplosticta*, *S*. *leptodactyla* and *S*. *phrynoderma*) are endemic to regions south of the Palghat gap, in the states of Kerala and Tamil Nadu. On the other hand, all the species in *Indirana beddomii* group and *Indirana semipalmata* group (except *I*. *semipalmata*) are restricted either to the north (*Indirana beddomii* group: *I*. *beddomii*, *I*. *bhadrai*, *I*. *leithii* and *I*. *tysoni*; *Indirana semipalmata* group: *I*. *chiravasi*, *I*. *duboisi*, *I*. *gundia*, *I*. *paramakri* and *I*. *salelkari*) or south (*Indirana beddomii* group: *I*. *brachytarsus*, *I*. *sarojamma* and *I*. *yadera*) of the Palghat gap (Fig 3). The exception–*Indirana semipalmata* is also largely restricted to the south of Palghat gap, but one population (from Siruvani) in our study was found north of this gap. *Indirana leithii* marks the northern most distribution limit of Leaping frogs, with its range restricted to the north of Goa gap, largely in the northern Western Ghats state of Maharashtra, as also discussed earlier [30]. In the present study, *Indirana* species were found between low-to mid elevations (66–1508 m asl) and many were usually observed close to human-altered landscapes in and around mixed deciduous (in Karnataka) to dry deciduous (Maharashtra) forests, which could make them prone to human-induced threats. On the other hand, *Sallywalkerana* species were found in mid-to high elevation forests (741–2310 m asl). Detailed natural history studies will be required to understand the various ecological adaptations of ranixalid members.

Ranixalid species have often been misidentified (e.g., [10, 11, 28, 29, 32, 58, 60–63]) due to their highly conserved external morphology resulting in taxonomic confusions and uncertainties. Dahanukar *et al*. [12] clarified the genetic identification of various previously available DNA sequences and the same is further confirmed in our study (Fig 1 and S2 Table). Besides this, we also provide additional distribution records for most of the previously known as well as all the recently described species, including many that were known only from their type localities. Hence, with the additional genetically and morphologically confirmed distribution records, descriptions of new species and new estimates of species diversity based on integrated evidence, our study provides an updated review of the family Ranixalidae. This will facilitate any future studies using integrated approaches to provide a better understanding of evolutionary relationships among ranixalid frogs.

Updated systematic revisions also provide vital information for conservation prioritization not only by enabling proper identification and range delineation of species, but also by facilitating conservation assessment of species (e.g., [64, 65]). According to the present conservation status of Leaping frogs [66], six species (genus *Indirana*: *I*. *brachytarsus*, *I*. *gundia* and *I*. *leithii*; genus *Sallywalkerana*: *S*. *diplosticta*, *S*. *leptodactyla* and *S*. *phrynoderma*) are already facing extinction threats, while others are unevaluated (genus *Indirana*: *I*. *chiravasi*, *I*. *duboisi*, *I*. *salelkari*, *I*. *sarojamma*, *I*. *tysoni* and *I*. *yadera*) (Fig 1C). With the availability of new information presented in this study, proper evaluation of all the previously known and newly described species will be facilitated. Since setting up of conservation priorities largely depend on the threat status of species [65], a reassessment of IUCN categorizations of all ranixalid species will be necessary for effective conservation of these relic frogs.

Through this work, we also draw attention to the taxonomic status of *Indirana longicrus* and *I*. *tenuilingua*, which have been considered as incertae sedis by Dahanukar *et al*. [12]. Longstanding taxonomic uncertainties regarding the status of these species [17] have existed largely due to unavailability of type specimens [31, 33] or new collections from the type locality. The new molecular and morphological data presented in our study can possibly yield different interpretations regarding the status of these two species. Though the original descriptions of *I*. *longicrus* and *I*. *tenuilingua* [13] discuss various ‘vague characters’ [12, 17], the type localities mentioned are not imprecise. Our genetic samples from Kempholey and vicinity are found to nest with the recently described *I*. *duboisi* [12] and *I*. *gundia* [9] (Fig 1 and S2 Table). Based on certain morphological characters (such as snout to vent length, tibium “longer than the thigh”, “arm equals the length of the snout”, tympanum “about half the diameter of the eye”) and the illustration accompanying the original description [13], our new collections from Kempholey and its vicinity could be assigned to *I*. *longicrus*, since this nominal taxon represents a member of the genus *Indirana*, as also previously noted by Bossuyt and Dubois [17]. However, in favour of maintaining taxonomic stability, we provisionally refrain from proposing new taxonomic actions to change the present status of *I*. *longicrus* and *I*. *tenuilingua*. At the same time, we find it important to point that available names should not be overlooked simply because of unavailability of type specimens and vague descriptions [17, 67, 68]. It would be prudent to acknowledge that taxonomy as a science has evolved drastically due to availability of modern tools and techniques (e.g., [69, 70]), which were unavailable for historical descriptions. Hence, it is advisable to refer to new collections from precisely known type localities for better insights and to use existing names, as much as possible (e.g., [36, 49, 53]), in order to allow ‘nomenclatural parsimony’ [71].

## Supporting Information

S1 Fig Ventral coloration and femoral glands in ranixalid species. (A, B) *Indirana semipalmata*, male (SDBDU 2015.3034) with femoral glands. (A) In life. (B) In preservation. (C) *I*. *gundia*, male (MNHN 1985.0633) with femoral glands (in preservation). (D) *I*. *brachytarsus*, male (SDBDU 2015.2931), without femoral glands (in life). (E) *Sallywalkerana leptodactyla*, male (SDBDU 2003.40336), without femoral glands (in life). (F) *S*. *phrynoderma*, female (SDBDU 2002.1 181), having a distinct bluish-black ventral surface with scattered grey color speckles (in life).
(PDF)

S2 Fig Lateral view of head in ranixalid species. (A–G) *Indirana beddomii* group. (A) *I*. *beddomii*, male (SDBDU 2010.225) and female (SDBDU 2011.961). (B) *I*. *bhadrai*, female (ZSI/WGRC/V/A887). (C) *I*. *brachytarsus*, male (SDBDU 2015.2931) and female (SDBDU 2012.814). (D) *I*. *leithii*, male (SDBDU 2014. 2515) and female (SDBDU 2014.2514). (E) *I*. *sarojamma*, male (SDBDU 2002.334) and female (SDBDU 2002.516). (F) *I*. *tysoni*, male (SDBDU 2012.74) and female (SDBDU 2012.73). (G) *I*. *yadera*, male (SDBDU 2012.2744) and female (SDBDU 2015.3155). (H–M) *Indirana semipalmata* group. (H) *I*. *chiravasi*, male (SDBDU 2012.2125) and female (SDBDU 2015.3087). (I) *I*. *duboisi*, male (SDBDU 2003.1086) and female (SDBDU 2011.1399). (J) *I*. *gundia*, male (MNHN 1985.0633) and female (MNHN 1985.0621). (K) *I*. *paramakri*, male (SDBDU 2005.3741) and female (ZSI/WGRC/V/A888). (L) *I*. *salelkari*, female (SDBDU 2011.1330). (M) *I*. *semipalmata*, male (SDBDU 2015.3035) and female (SDBDU 2006.4773A). (N–P) Genus *Sallywalkerana*. (N) *S*. *diplosticta*, male (SDBDU 2003.40103) and female (SDBDU 2002.513). (O) *S*. *leptodactyla*, male (SDBDU 2004.40336) and female (SDBDU 2013.911). (P) *S*. *phrynoderma*, male (SDBDU 2002.1181).
(PDF)

S3 Fig Dorsal (left) and ventral (right) views of head in ranixalid species. (A–G) *Indirana beddomii* group. (A) *I*. *beddomii*, female (SDBDU 2011.961). (B) *I*. *bhadrai*, female (ZSI/WGRC/V/A887). (C) *I*. *brachytarsus*, female (SDBDU 2002.4091). (D) *I*. *leithii*, female (SDBDU 2002.2010). (E) *I*. *sarojamma*, female (SDBDU 2002.516). (F) *I*. *tysoni*, female (SDBDU 2012.73). (G) *I*. *yadera*, male (SDBDU 2012.2744). (H–M) *Indirana semipalmata* group. (H) *I*. *chiravasi*, female (SDBDU 2015.3087). (I) *I*. *duboisi*, male (SDBDU 2003.1086). (J) *I*. *gundia*, male (MNHN 1985.0633). (K) *I*. *paramakri*, female (ZSI/WGRC/V/A888). (L) *I*. *salelkari*, female (SDBDU 2011.1330). (M) *I*. *semipalmata*, female (SDBDU 2006.4773). (N–P) Genus *Sallywalkerana*. (N) *S*. *diplosticta*, female (SDBDU 2002.1249). (O) *S*. *leptodactyla*, female (SDBDU 2002.917). (P) *S*. *phrynoderma*, male (SDBDU 2002.1181).
(PDF)

S4 Fig *Indirana beddomii* group in preservation. From left to right: Dorsal view, ventral view, lateral view of head, ventral view of hand, ventral view of foot. (A–E) Lectotype of *Polypedates beddomii* (= *Indirana beddomii*), NHM 74.4.29.208 (ex BMNH 1947.2.27.72), female. (F–J) Lectotype of *Polypedates brachytarsus* (= *Indirana brachytarsus*), NHM 74.4.29.1307 (ex BMNH 1947.2.27.92), female.
(PDF)

S5 Fig *Indirana beddomii* group in preservation. From left to right: Dorsal view, ventral view, lateral view of head, ventral view of hand, ventral view of foot. (A–E) Holotype of *Rana leithii* (= *Indirana leithii*), NHM 69.8.28.50 (ex BMNH 1947.2.28.17), female. (F–J) *Indirana sarojamma*, SDBDU 2002.516, female.
(PDF)

S6 Fig *Indirana beddomii* group in preservation. From left to right: Dorsal view, ventral view, lateral view of head, ventral view of hand, ventral view of foot. (A–E) *Indirana tysoni*, SDBDU 2012.73, female. (F–J) *Indirana yadera*, SDBDU 2012.2744, male.
(PDF)

S7 Fig *Indirana semipalmata* group in preservation. From left to right: Dorsal view, ventral view, lateral view of head, ventral view of hand, ventral view of foot. (A–E) *Indirana chiravasi*, SDBDU 2015.3087, female. (F–J) *Indirana duboisi*, SDBDU 2003.1086, male.
(PDF)

S8 Fig *Indirana semipalmata* group in preservation. From left to right: Dorsal view, ventral view, lateral view of head, ventral view of hand, ventral view of foot. (A–E) Holotype of *Ranixalus gundia* (= *Indirana gundia*), MNHN 1985.0633, male. (F–J) *Indirana salelkari*, SDBDU 2011.1330, female. (K–O) Lectotype of *Rana semipalmata* (= *Indirana semipalmata*), NHM 74.4.29.605 (ex BMNH 1947.2.29.50), female.
(PDF)

S9 Fig Genus *Sallywalkerana* in preservation. From left to right: Dorsal view, ventral view, lateral view of head, ventral view of hand, ventral view of foot. (A–E) Lectotype of *Ixalus diplostictus* (= *Sallywalkerana diplosticta*), NHM 74.4.29.1412 (ex BMNH 1947.2.2.21), female. (F–J) Lectotype of *Rana leptodactyla* (= *Sallywalkerana leptodactyla*), NHM 74.4.29.593 (ex BMNH 1947.2.29.39), female. (K–O) Lectotype of *Rana phrynoderma* (= *Sallywalkerana phrynoderma*), NHM 82.2.10.21 (ex BMNH 1947.2.3.8), female.
(PDF)

S1 File Taxonomic accounts of previously known ranixalid species. (PDF)

S1 Table Collection localities of ranixalid species reported in this study. Localities are arranged by State.
(PDF)

S2 Table List of DNA sequences included in the study. (PDF)

S3 Table Uncorrected intraspecific pairwise distances between 16S mitochondrial gene sequences. The table gives mean and standard deviation values over all pairwise comparisons among individuals or populations of a species. N is the number of individuals for each species. The original p-distances are shown in percentage.
(PDF)

S4 Table Uncorrected interspecific pairwise distances between 16S mitochondrial gene sequences. The table gives mean and standard deviation values over all pairwise comparisons of individuals sequenced from the two taxa being compared. N is the number of pairwise comparisons. N1 and N2 represent number of individuals for Taxon 1 and Taxon 2, respectively. The original p-distances are shown in percentage.
(PDF)
