# Supplementary material for: Molecular and Morphological Study of Leaping Frogs (Anura, Ranixalidae) with Description of Two New Species
Source: PLoS One. 2016 Nov 16;11(11):e0166326. doi: 10.1371/journal.pone.0166326 (PMC5112961; doi:10.1371/journal.pone.0166326)
Supplement: S1 Fig — (A, B) Indirana semipalmata, male (SDBDU 2015.3034) with femoral glands. (A) In life. (B) In preservation. (C) I. gundia, male (MNHN 1985.0633) with femoral glands (in preservation). (D) I. brachytarsus, male (SDBDU 2015.2931), without femoral glands (in life). (E) Sallywalkerana leptodactyla, male (SDBDU 2003.40336), without femoral glands (in life). (F) S. phrynoderma, female (SDBDU 2002.1 181), having a distinct bluish-black ventral surface with scattered grey color speckles (in life). (PDF) [file pone.0166326.s001.pdf]

Supporting information **Figure 1**

Molecular and morphological study of Leaping frogs (Anura, Ranixalidae) with description of two new species

Sonali Garg and SD Biju | PLoS One 2016

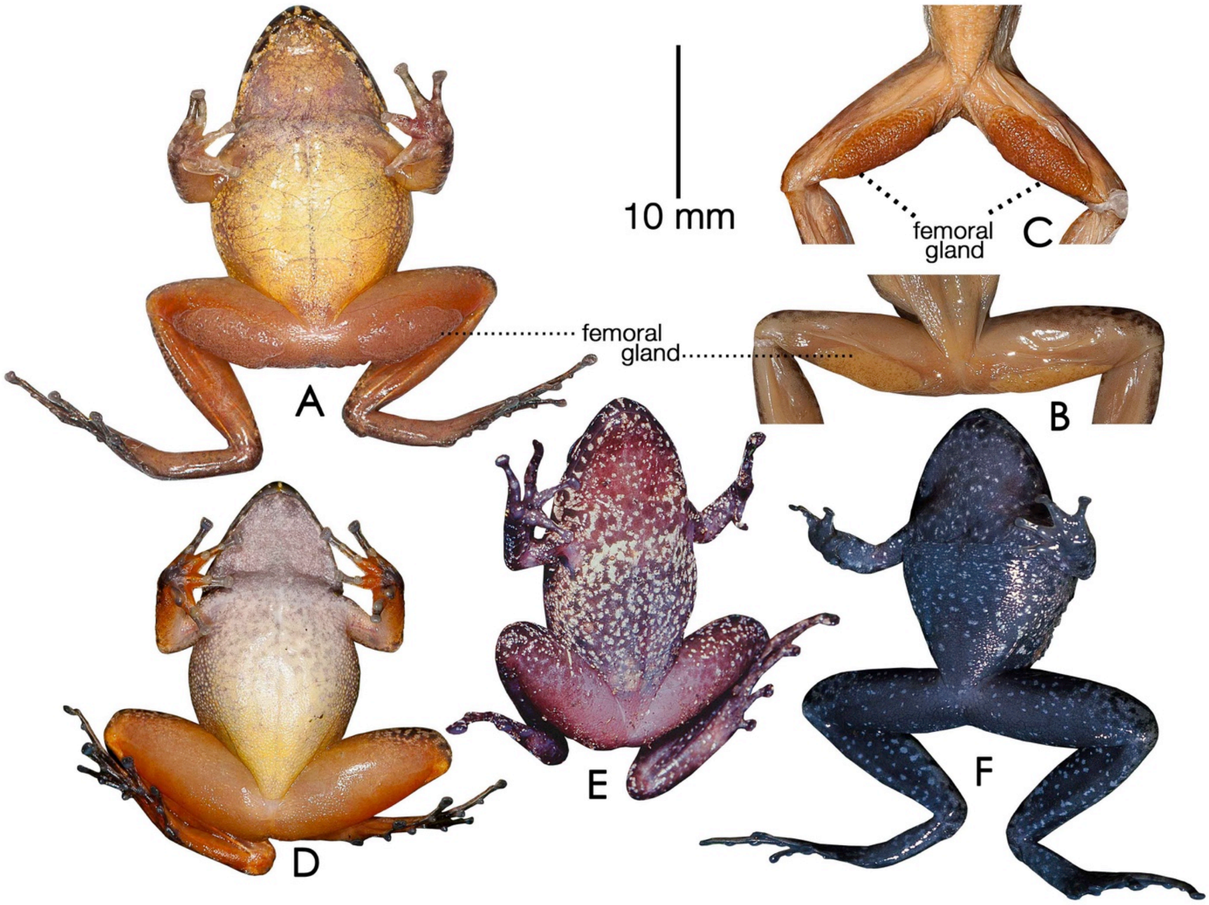

S1 Fig. Ventral coloration and femoral glands in ranixalid species. (A, B) *Indirana semipalmata*, male (SDBDU 2015.3034) with femoral glands. (A) In life. (B) In preservation. (C) *I. gundia*, male (MNHN 1985.0633) with femoral glands (in preservation). (D) *I. brachytarsus*, male (SDBDU 2015.2931), without femoral glands (in life). (E) *Sallywalkerana leptodactyla*, male (SDBDU 2003.40336), without femoral glands (in life). (F) *S. phrynoderma*, female (SDBDU 2002.1 181), having a distinct bluish-black ventral surface with scattered grey color speckles (in life).
