# Supplementary material for: Molecular and Morphological Study of Leaping Frogs (Anura, Ranixalidae) with Description of Two New Species
Source: PLoS One. 2016 Nov 16;11(11):e0166326. doi: 10.1371/journal.pone.0166326 (PMC5112961; doi:10.1371/journal.pone.0166326)
Supplement: S2 Fig — (A–G) Indirana beddomii group. (A) I. beddomii, male (SDBDU 2010.225) and female (SDBDU 2011.961). (B) I. bhadrai, female (ZSI/WGRC/V/A887). (C) I. brachytarsus, male (SDBDU 2015.2931) and female (SDBDU 2012.814). (D) I. leithii, male (SDBDU 2014. 2515) and female (SDBDU 2014.2514). (E) I. sarojamma, male (SDBDU 2002.334) and female (SDBDU 2002.516). (F) I. tysoni, male (SDBDU 2012.74) and female (SDBDU 2012.73). (G) I. yadera, male (SDBDU 2012.2744) and female (SDBDU 2015.3155). (H–M) Indirana semipalmata group. (H) I. chiravasi, male (SDBDU 2012.2125) and female (SDBDU 2015.3087). (I) I. duboisi, male (SDBDU 2003.1086) and female (SDBDU 2011.1399). (J) I. gundia, male (MNHN 1985.0633) and female (MNHN 1985.0621). (K) I. paramakri, male (SDBDU 2005.3741) and female (ZSI/WGRC/V/A888). (L) I. salelkari, female (SDBDU 2011.1330). (M) I. semipalmata, male (SDBDU 2015.3035) and female (SDBDU 2006.4773A). (N–P) Genus Sallywalkerana. (N) S. diplosticta, male (SDBDU 2003.40103) and female (SDBDU 2002.513). (O) S. leptodactyla, male (SDBDU 2004.40336) and female (SDBDU 2013.911). (P) S. phrynoderma, male (SDBDU 2002.1181). (PDF) [file pone.0166326.s002.pdf]

Supporting information **Figure 2**

Molecular and morphological study of Leaping frogs (Anura, Ranixalidae) with description of two new species

Sonali Garg and SD Biju | PLoS One 2016

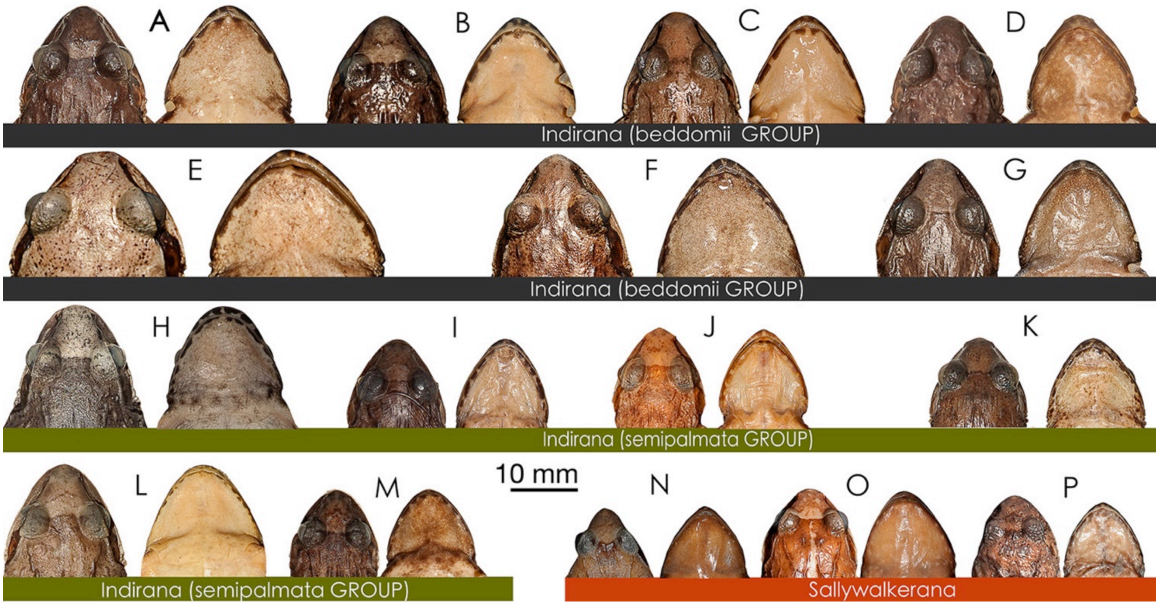

S2 Fig. Lateral view of head in ranixalid species. (A–G) *Indirana beddomii* group. (A) *I. beddomii*, male (SDBDU 2010.225) and female (SDBDU 2011.961). (B) *I. bhadrai*, female (ZSI/WGRC/V/A887). (C) *I. brachytarsus*, male (SDBDU 2015.2931) and female (SDBDU 2012.814). (D) *I. leithii*, male (SDBDU 2014. 2515) and female (SDBDU 2014.2514). (E) *I. sarojamma*, male (SDBDU 2002.334) and female (SDBDU 2002.516). (F) *I. tysoni*, male (SDBDU 2012.74) and female (SDBDU 2012.73). (G) *I. yadera*, male (SDBDU 2012.2744) and female (SDBDU 2015.3155). (H–M) *Indirana semipalmata* group. (H) *I. chiravasi*, male (SDBDU 2012.2125) and female (SDBDU 2015.3087). (I) *I. duboisi*, male (SDBDU 2003.1086) and female (SDBDU 2011.1399). (J) *I. gundia*, male (MNHN 1985.0633) and female (MNHN 1985.0621). (K) *I. paramakri*, male (SDBDU 2005.3741) and female (ZSI/WGRC/V/A888). (L) *I. salelkari*, female (SDBDU 2011.1330). (M) *I. semipalmata*, male (SDBDU 2015.3035) and female (SDBDU 2006.4773A). (N–P) Genus *Sallywalkerana*. (N) *S. diplosticta*, male (SDBDU 2003.40103) and female (SDBDU 2002.513). (O) *S. leptodactyla*, male (SDBDU 2004.40336) and female (SDBDU 2013.911). (P) *S. phrynoderma*, male (SDBDU 2002.1181).
