# Supplementary material for: Molecular and Morphological Study of Leaping Frogs (Anura, Ranixalidae) with Description of Two New Species
Source: PLoS One. 2016 Nov 16;11(11):e0166326. doi: 10.1371/journal.pone.0166326 (PMC5112961; doi:10.1371/journal.pone.0166326)
Supplement: S3 Fig — (A–G) Indirana beddomii group. (A) I. beddomii, female (SDBDU 2011.961). (B) I. bhadrai, female (ZSI/WGRC/V/A887). (C) I. brachytarsus, female (SDBDU 2002.4091). (D) I. leithii, female (SDBDU 2002.2010). (E) I. sarojamma, female (SDBDU 2002.516). (F) I. tysoni, female (SDBDU 2012.73). (G) I. yadera, male (SDBDU 2012.2744). (H–M) Indirana semipalmata group. (H) I. chiravasi, female (SDBDU 2015.3087). (I) I. duboisi, male (SDBDU 2003.1086). (J) I. gundia, male (MNHN 1985.0633). (K) I. paramakri, female (ZSI/WGRC/V/A888). (L) I. salelkari, female (SDBDU 2011.1330). (M) I. semipalmata, female (SDBDU 2006.4773). (N–P) Genus Sallywalkerana. (N) S. diplosticta, female (SDBDU 2002.1249). (O) S. leptodactyla, female (SDBDU 2002.917). (P) S. phrynoderma, male (SDBDU 2002.1181). (PDF) [file pone.0166326.s003.pdf]

Supporting information **Figure 3**

Molecular and morphological study of Leaping frogs (Anura, Ranixalidae) with description of two new species

Sonali Garg and SD Biju | PLoS One 2016

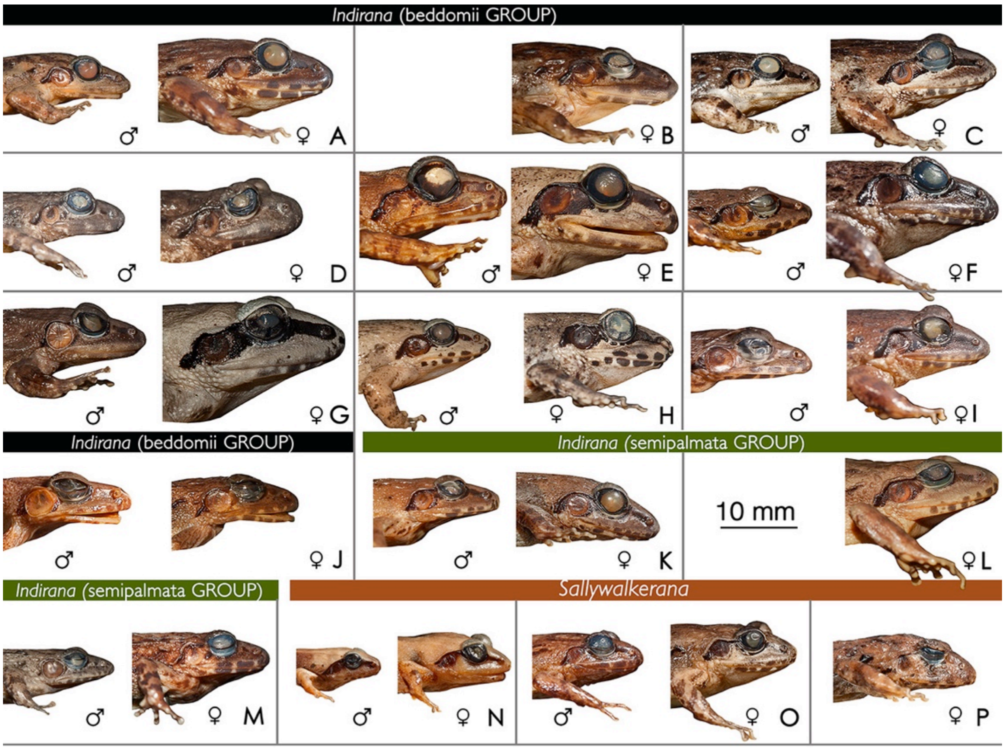

S3 Fig. Dorsal (left) and ventral (right) views of head in ranixalid species. (A–G) *Indirana beddomii* group. (A) *I. beddomii*, female (SDBDU 2011.961). (B) *I. bhadrai*, female (ZSI/WGRC/V/A887). (C) *I. brachytarsus*, female (SDBDU 2002.4091). (D) *I. leithii*, female (SDBDU 2002.2010). (E) *I. sarojamma*, female (SDBDU 2002.516). (F) *I. tysoni*, female (SDBDU 2012.73). (G) *I. yadera*, male (SDBDU 2012.2744). (H–M) *Indirana semipalmata* group. (H) *I. chiravasi*, female (SDBDU 2015.3087). (I) *I. duboisi*, male (SDBDU 2003.1086). (J) *I. gundia*, male (MNHN 1985.0633). (K) *I. paramakri*, female (ZSI/WGRC/V/A888). (L) *I. salelkari*, female (SDBDU 2011.1330). (M) *I. semipalmata*, female (SDBDU 2006.4773). (N–P) Genus *Sallywalkerana*. (N) *S. diplosticta*, female (SDBDU 2002.1249). (O) *S. leptodactyla*, female (SDBDU 2002.917). (P) *S. phrynoderma*, male (SDBDU 2002.1181).
