# Supplementary material for: Molecular and Morphological Study of Leaping Frogs (Anura, Ranixalidae) with Description of Two New Species
Source: PLoS One. 2016 Nov 16;11(11):e0166326. doi: 10.1371/journal.pone.0166326 (PMC5112961; doi:10.1371/journal.pone.0166326)
Supplement: S4 Fig — From left to right: Dorsal view, ventral view, lateral view of head, ventral view of hand, ventral view of foot. (A–E) Lectotype of Polypedates beddomii (= Indirana beddomii), NHM 74.4.29.208 (ex BMNH 1947.2.27.72), female. (F–J) Lectotype of Polypedates brachytarsus (= Indirana brachytarsus), NHM 74.4.29.1307 (ex BMNH 1947.2.27.92), female. (PDF) [file pone.0166326.s004.pdf]

Supporting information Figure 4

Molecular and morphological study of Leaping frogs (Anura, Ranixalidae) with description of two new species

Sonali Garg and SD Biju | PLoS One 2016

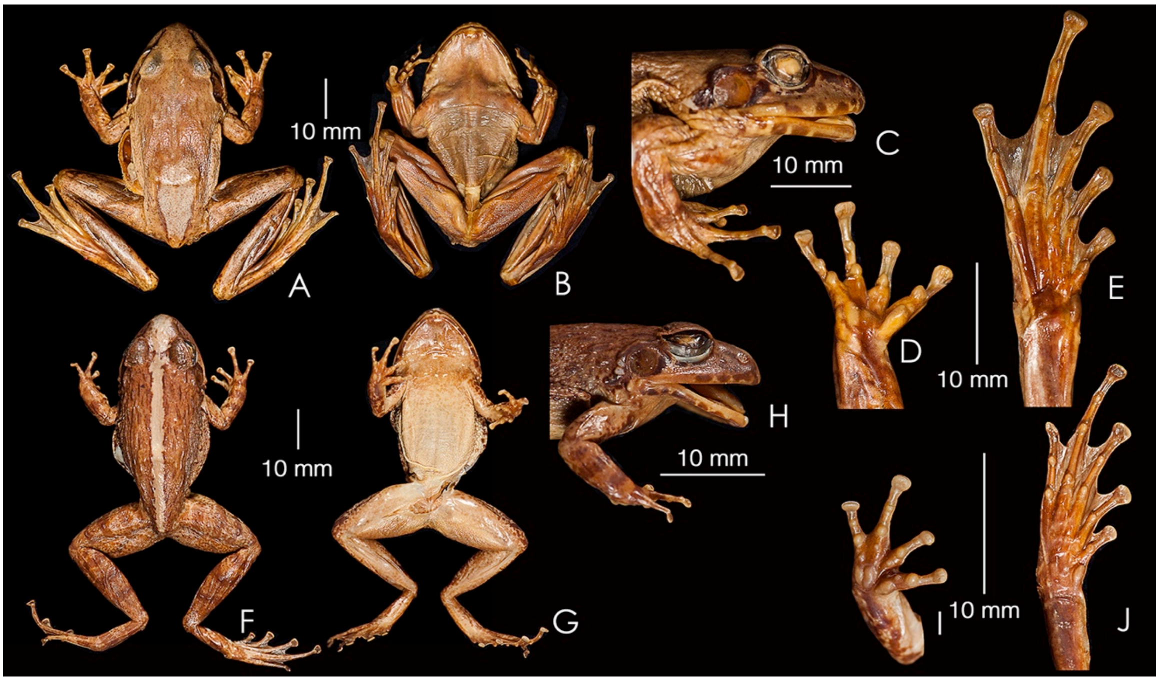

S4 Fig. *Indirana beddomii* group in preservation. From left to right: Dorsal view, ventral view, lateral view of head, ventral view of hand, ventral view of foot. (A–E) Lectotype of *Polypedates beddomii* (= *Indirana beddomii*), NHM 74.4.29.208 (ex BMNH 1947.2.27.72), female. (F–J) Lectotype of *Polypedates brachytarsus* (= *Indirana brachytarsus*), NHM 74.4.29.1307 (ex BMNH 1947.2.27.92), female.
