# Supplementary material for: Molecular and Morphological Study of Leaping Frogs (Anura, Ranixalidae) with Description of Two New Species
Source: PLoS One. 2016 Nov 16;11(11):e0166326. doi: 10.1371/journal.pone.0166326 (PMC5112961; doi:10.1371/journal.pone.0166326)
Supplement: S5 Fig — From left to right: Dorsal view, ventral view, lateral view of head, ventral view of hand, ventral view of foot. (A–E) Holotype of Rana leithii (= Indirana leithii), NHM 69.8.28.50 (ex BMNH 1947.2.28.17), female. (F–J) Indirana sarojamma, SDBDU 2002.516, female. (PDF) [file pone.0166326.s005.pdf]

Supporting information **Figure 5**

Molecular and morphological study of Leaping frogs (Anura, Ranixalidae) with description of two new species

Sonali Garg and SD Biju | PLoS One 2016

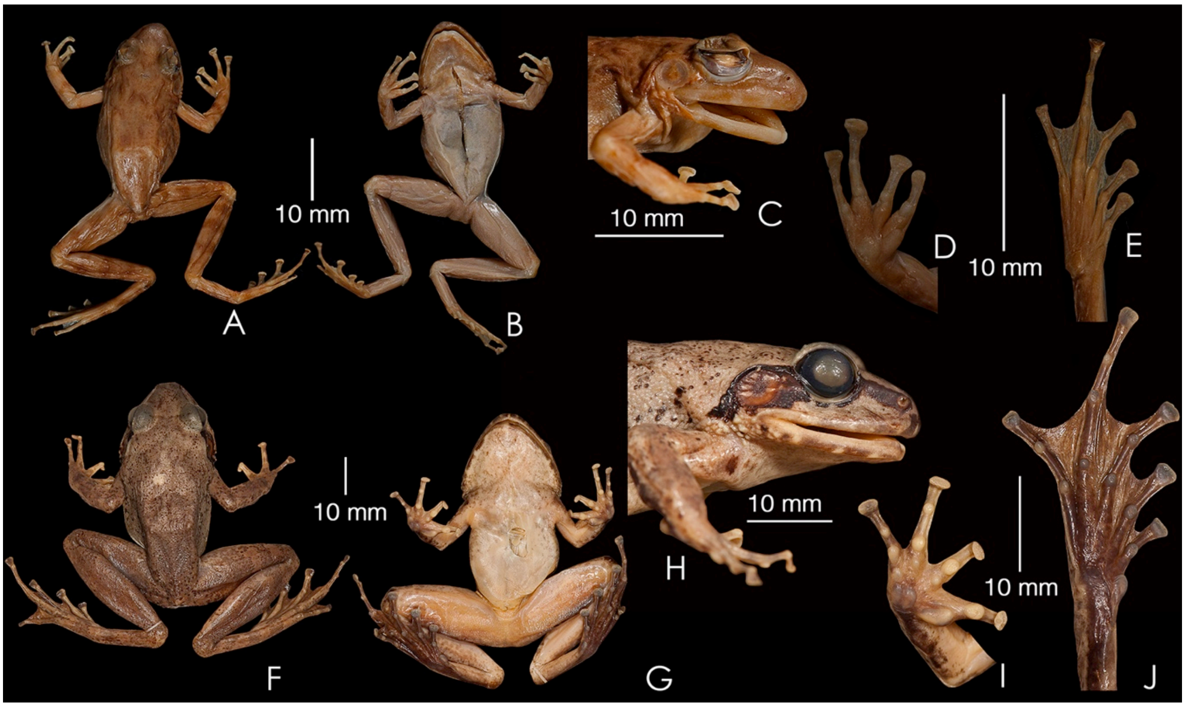

S5 Fig. *Indirana beddomii* group in preservation. From left to right: Dorsal view, ventral view, lateral view of head, ventral view of hand, ventral view of foot. (A–E) Holotype of *Rana leithii* (= *Indirana leithii*), NHM 69.8.28.50 (ex BMNH 1947.2.28.17), female. (F–J) *Indirana sarojamma*, SDBDU 2002.516, female.
