# Supplementary material for: Molecular and Morphological Study of Leaping Frogs (Anura, Ranixalidae) with Description of Two New Species
Source: PLoS One. 2016 Nov 16;11(11):e0166326. doi: 10.1371/journal.pone.0166326 (PMC5112961; doi:10.1371/journal.pone.0166326)
Supplement: S8 Fig — From left to right: Dorsal view, ventral view, lateral view of head, ventral view of hand, ventral view of foot. (A–E) Holotype of Ranixalus gundia (= Indirana gundia), MNHN 1985.0633, male. (F–J) Indirana salelkari, SDBDU 2011.1330, female. (K–O) Lectotype of Rana semipalmata (= Indirana semipalmata), NHM 74.4.29.605 (ex BMNH 1947.2.29.50), female. (PDF) [file pone.0166326.s008.pdf]

Supporting information **Figure 8**

Molecular and morphological study of Leaping frogs (Anura, Ranixalidae) with description of two new species

Sonali Garg and SD Biju | PLoS One 2016

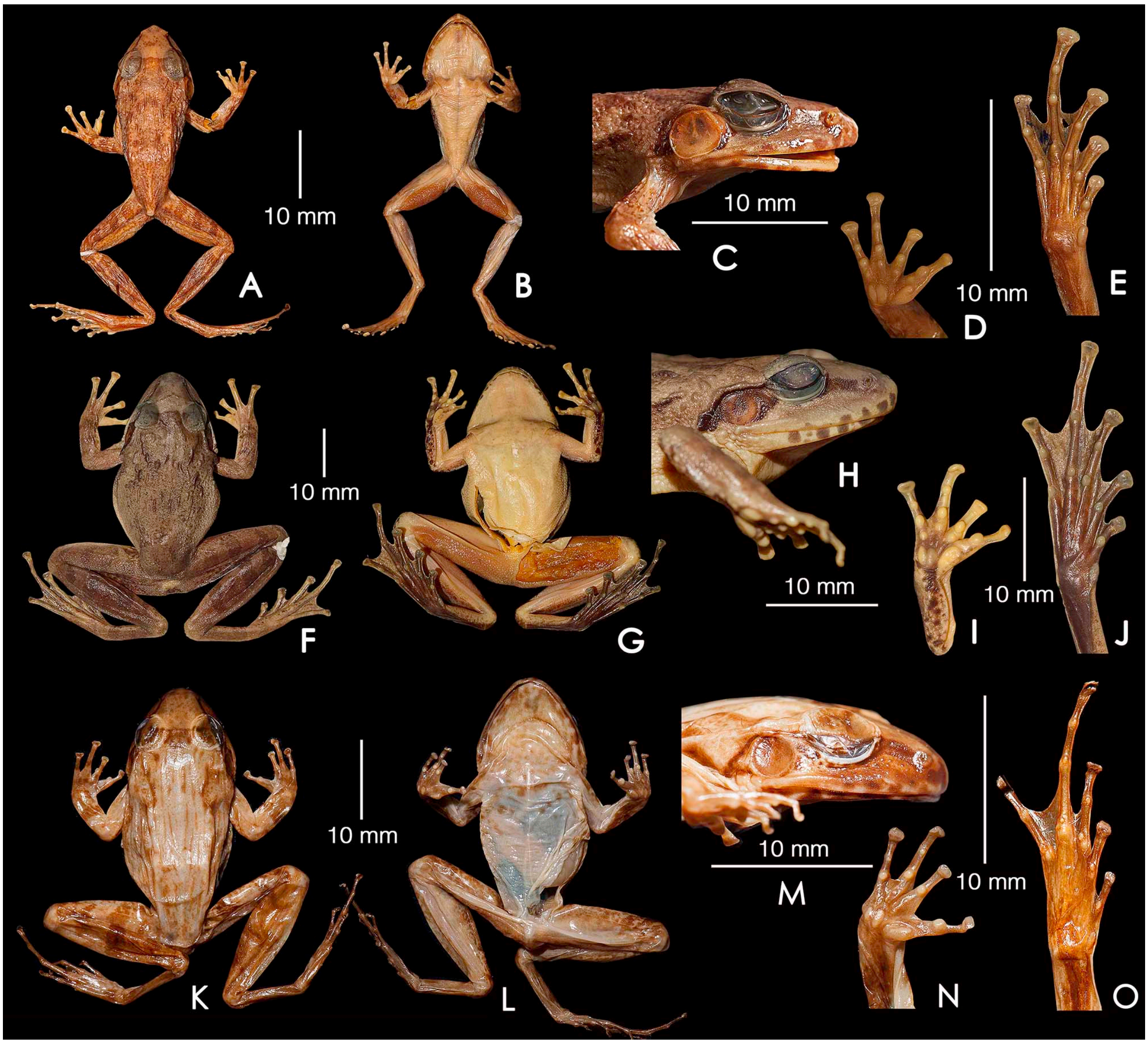

S8 Fig. *Indirana semipalmata* group in preservation. From left to right: Dorsal view, ventral view, lateral view of head, ventral view of hand, ventral view of foot. (A–E) Holotype of *Ranixalus gundia* (= *Indirana gundia*), MNHN 1985.0633, male. (F–J) *Indirana salelkari*, SDBDU 2011.1330, female. (K–O) Lectotype of *Rana semipalmata* (= *Indirana semipalmata*), NHM 74.4.29.605 (ex BMNH 1947.2.29.50), female.
