# Supplementary material for: Molecular and Morphological Study of Leaping Frogs (Anura, Ranixalidae) with Description of Two New Species
Source: PLoS One. 2016 Nov 16;11(11):e0166326. doi: 10.1371/journal.pone.0166326 (PMC5112961; doi:10.1371/journal.pone.0166326)
Supplement: S9 Fig — From left to right: Dorsal view, ventral view, lateral view of head, ventral view of hand, ventral view of foot. (A–E) Lectotype of Ixalus diplostictus (= Sallywalkerana diplosticta), NHM 74.4.29.1412 (ex BMNH 1947.2.2.21), female. (F–J) Lectotype of Rana leptodactyla (= Sallywalkerana leptodactyla), NHM 74.4.29.593 (ex BMNH 1947.2.29.39), female. (K–O) Lectotype of Rana phrynoderma (= Sallywalkerana phrynoderma), NHM 82.2.10.21 (ex BMNH 1947.2.3.8), female. (PDF) [file pone.0166326.s009.pdf]

Supporting information **Figure 9**

Molecular and morphological study of Leaping frogs (Anura, Ranixalidae) with description of two new species

Sonali Garg and SD Biju | PLoS One 2016

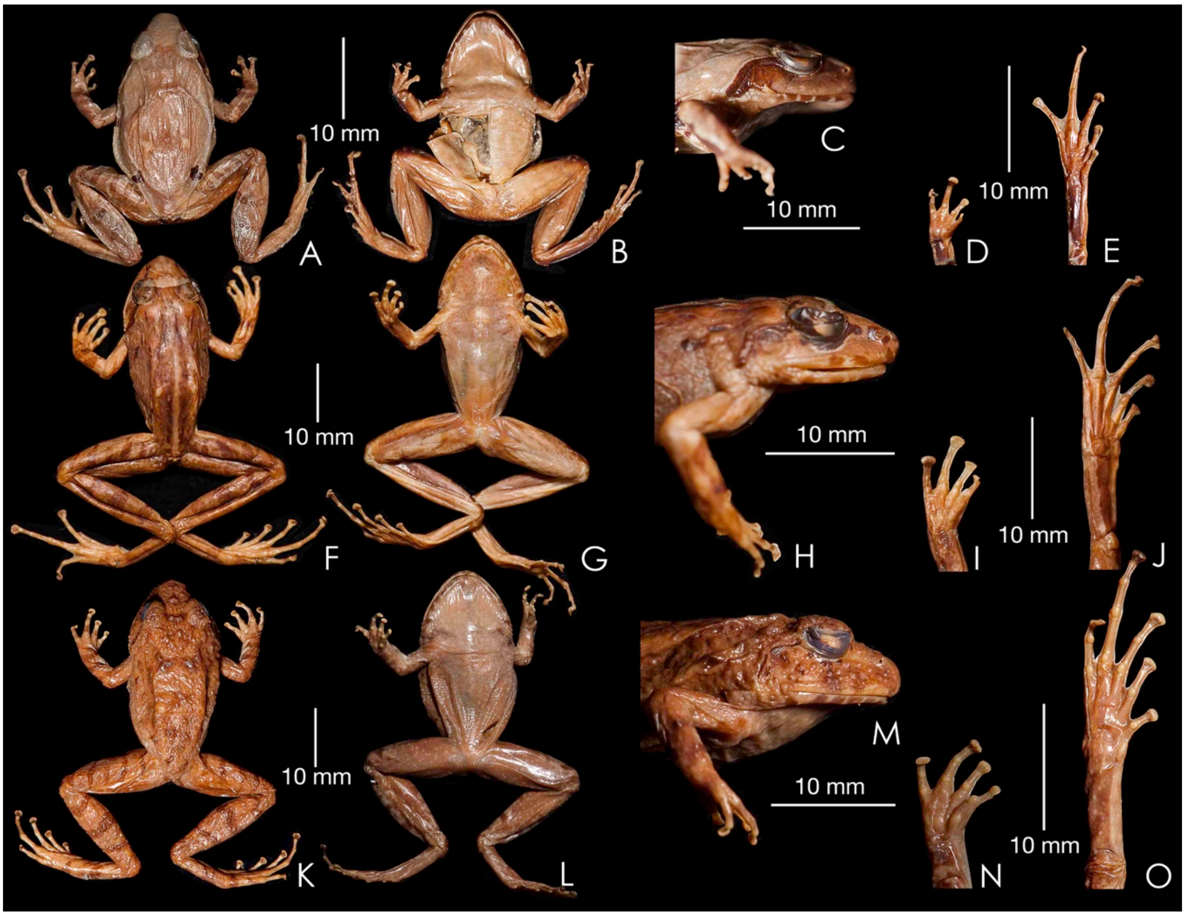

S9 Fig. Genus *Sallywalkerana* in preservation. From left to right: Dorsal view, ventral view, lateral view of head, ventral view of hand, ventral view of foot. (A–E) Lectotype of *Ixalus diplostictus* (= *Sallywalkerana diplosticta*), NHM 74.4.29.1412 (ex BMNH 1947.2.2.21), female. (F–J) Lectotype of *Rana leptodactyla* (= *Sallywalkerana leptodactyla*), NHM 74.4.29.593 (ex BMNH 1947.2.29.39), female. (K–O) Lectotype of *Rana phrynoderma* (= *Sallywalkerana phrynoderma*), NHM 82.2.10.21 (ex BMNH 1947.2.3.8), female.
