# Supplementary material for: Molecular and Morphological Study of Leaping Frogs (Anura, Ranixalidae) with Description of Two New Species
Source: PLoS One. 2016 Nov 16;11(11):e0166326. doi: 10.1371/journal.pone.0166326 (PMC5112961; doi:10.1371/journal.pone.0166326)
Supplement: S1 File — (PDF) [file pone.0166326.s010.pdf]

Supporting information: **S1 File**

Molecular and morphological study of Leaping frogs (Anura, Ranixalidae) with description of two new species

Sonali Garg, SD Biju

S1 File. Taxonomic accounts of previously known ranixalid species.

Genus *Indirana*

Indirana beddomii group

Indirana beddomii (Günther 1876)
Beddome's Leaping Frog [58]
(Figs 1–6; S2–S4 Figs; S1–S4 Tables)

Comparison. Based on the overall morphology, *Indirana beddomii* could be confused with *I. bhadrai*, *I. brachytarsus, I. leithii*, *I. sarojamma*, *I. tysoni* and *I. yadera* within the *Indirana beddomii* group. However, *I. beddomii* differs from *I. bhadrai*, *I. brachytarsus* and *I. leithii* first finger longer than second finger, male FIL 4.1 ± 1.3 mm, *N* = 2, female FIL 6.9 ± 0.6 mm, *N* = 5 vs. male FIIL 3.1 ± 1.0 mm, *N* = 2, female FIIL 5.5 ± 0.3 mm, *N* = 5 (vs. nearly equal to second finger, male FIL 5.1 mm, *N* = 1, female FIL 6.1 mm, *N* = 1 vs. male FIIL 5.2 mm, *N* = 1, female FIIL 6.2 mm, *N* = 1 in *I. bhadrai*; equal to second finger, male FIL 3.3 ± 0.4 mm, *N* = 3, female FIL 5.0 ± 0.7 mm, *N* = 6 vs. male FIIL 3.3 ± 0.3 mm, *N* = 3, female FIIL 5.0 ± 0.6 mm, *N* = 6 in *I. brachytarsus*; shorter, male FIL 2.6 ± 0.2 mm, *N* = 3, female FIL 3.4 ± 0.1 mm, *N* = 5 vs. male FIIL 3.8 ± 0.2 mm, *N* = 3, female FIIL 4.5 ± 0.3 mm, *N* = 5 in *I. leithii*) (Fig 4); specifically also differs from *I. bhadrai* by its snout rounded in dorsal view (vs. nearly pointed), rounded in lateral view (vs. nearly acute), loreal region obtuse (vs. acute), and third toe webbing up to the second subarticular tubercle on the inside, I1–2II1–2III1–$2^{1}/_{2}$IV$^{1}/_{2}$–1V (vs. below, I1–2II1–$2^{1}/_{4}$III1–3^{-} IV3^{-}–1V) (Fig 5); and from *I. leithii* by its larger adult size, male SVL 31.7 ± 4.9 mm, *N* = 2, female SVL 47.7 ± 4.5 mm, *N* = 5 (vs. smaller, male SVL 27.2 ± 1.8 mm, *N* = 3, female SVL 34.4 ± 2.0 mm, *N* = 5). *I. beddomii* differs from *I. sarojamma*, *I. tysoni* and *I. yadera* by its head rounded in dorsal view (vs. nearly truncate, and sub-ovoid in *I. tysoni* and *I. yadera*); specifically also differs from *I. tysoni* by its fourth toe webbing extending beyond the second subarticular tubercle on either side (vs. below); and from *I. yadera* by its relatively smaller adult snout-vent size male SVL 31.7 ± 4.9 mm, *N* = 2, female SVL 47.7 ± 4.5 mm, *N* = 5 (vs. SVL 42.4 ± 2.8 mm, *N* = 3, female SVL 58.6 mm, *N* = 1) and loreal region obtuse (vs. acute). Detailed comparisons with all members of the proposed *Indirana beddomii* group, except *I. bhadrai*, was also provided by Dahanukar *et al*. [12].

For better clarity, we compare *I. beddomii* with all the members of *Indirana semipalmata* group from which this species differs by its first finger longer than second finger (vs. equal or nearly equal) (Fig 4). Furthermore, *I. beddomii* differs from all species currently known in the genus *Sallywalkerana* by its larger adult snout-vent size, male SVL 31.7 ± 4.9 mm, *N* = 2, female

SVL 47.7 ± 4.5 mm, *N* = 5 (vs. smaller); first finger longer than second finger (vs. shorter), and fourth toe webbing extending above the second subarticular tubercle on either side, I1–2II1–2III1–$2^1/_2$IV$2^1/_2$–1V (vs. just above the third subarticular tubercle) (Fig 5).

Description of lectotype *(measurements in mm)*. Adult female (SVL 55.5); head wider (HW 23.3) than long (HL 22.5); snout nearly truncate in dorsal view, sub-ovoid in ventral view, obtuse in lateral view, its length (SL 9.5) longer than horizontal diameter of eye (EL 5.9); loreal region obtuse and concave with rounded canthus rostralis; interorbital space (IUE 5.5) wider than upper eyelid (UEW 4.1); tympanum (TYD 4.2) 71% of eye diameter (EL 5.9). Forearm (FAL 11.9) shorter than hand length (HAL 14.9), finger length formula: II<IV<I<III, finger discs moderately wide compared to finger width (FD_I 1.9, FW_I 0.6; FD_{II} 1.8, FW_{II} 0.6; FD_{III} 1.9, FW_{III} 0.7; FD_{IV} 2.1, FW_{IV} 0.7). Thigh (TL 34.0) shorter than shank (SHL 36.1), and longer than foot (FOL 32.0), toe discs wide compared to toe width (TD_I 1.7, TW_I 0.6; TD_{II} 1.9, TW_{II} 0.7; TD_{III} 2.5, TW_{III} 0.7; TD_{IV} 2.4, TW_{IV} 0.6; TD_V 1.9, TW_V 0.7), foot webbing: I1–2II1–2III1–3IV3–1V (Fig 5).

Dorsal skin sparsely granular with faintly developed discontinuous folds. Ventral surfaces of throat and chest smooth, abdomen and posterior parts of thigh granular (Fig 6, S4 Fig).

Colour of lectotype. *In preservation*. Dorsum light brown with irregular dark spots; snout lighter in colour than dorsum; margins of upper and lower jaw with alternate dark brown and cream coloured cross-bars (S4 Fig); a dark brown band extending from the tip of the snout through the lower margin of eye, widening behind the eye and over the tympanum, and ending near the armpit on either sides of the head; tympanum dark brown. *In life* (SDBDU 2010.255). See Figure 6A, and the detailed description provided by Inger *et al*. [59] and Dahanukar *et al*. [12].

Secondary sexual characters. *Male* (SDBDU 2010.225), femoral glands absent, nuptial pads present; *female* (SDBDU 2011.961), large pigmented eggs (diameter 0.6–0.8 mm, *N* = 25).

Variations. SDBDU 2010.255 (male): dorsum yellowish-brown in colour, sparse spinular projections on feet and margins of lower jaw (Fig 6A); SDBDU 2011.1269 (female): dorsum with a mid dorsal line extending from snout to vent.

Specimens examined. Males. Kerala state: Kozhikode district. SDBDU 2010.225, an adult male, from Kakkayam, collected by SDB and team, 27 May 2010. Palakkad district. SDBDU 2011.1270, an adult male, from Siruvani, Kuddam, collected by SDB and SG, 18 October 2011. **Females.** Kerala state: Palakkad district. SDBDU 2011.960–961, two adult females, from Sairandhri, Silent Valley, collected by SDB and SG on 18 September 2011; SDBDU 2011.1269, an adult female, from Siruvani, Kuddam, collected by SDB and SG, 18 October 2011. Wayanad district. SDBDU 2011.850, an adult female, Settukunnu, collected by SDB and team on 15 August 2011. Colonial locality: "Malabar". NHM 74.4.29.208 (ex 1947.2.27.72), Col. Beddome.

Indirana brachytarsus (Günther 1876)
Short-legged Leaping Frog [58]
(Figs 1–6; S1–S4 Figs; S1–S4 Tables)

Comparison. Within the *Indirana beddomii* group, *I. brachytarsus* differs from *I. leithii*, *I. sarojamma*, *I. tysoni* and *I. yadera* by its first finger equal to second finger, male FIL 3.3 ± 0.4 mm, *N* = 3, female FIL 5.0 ± 0.7 mm, *N* = 6 vs. male FIIL 3.3 ± 0.3 mm, *N* = 3, female FIIL 5.0 ± 0.6 mm, *N* = 6 (vs. shorter, male FIL 2.6 ± 0.2 mm, *N* = 3, female FIL 3.4 ± 0.1 mm, *N* = 5 vs.

male FIIL 3.8 ± 0.2 mm, *N* = 3, female FIIL 4.5 ± 0.3 mm, *N* = 5 in *I. leithii*; longer in *I. sarojamma*, *I. tysoni* and *I. yadera*: male FIL 4.9 ± 0.1 mm, *N* = 2, female FIL 8.1 mm, *N* = 1 vs. male FIIL 3.8 ± 0.4 mm, *N* = 2, female FIIL 6.9 mm, *N* = 1 in *I. sarojamma*, male FIL 4.6 ± 0.4 mm, *N* = 4, female FIL 7.1 ± 0.3 mm, *N* = 2 vs. male FIIL 3.4 ± 0.1 mm, *N* = 4, female FIIL 5.9 ± 0.4 mm, *N* = 2 in *I. tysoni*, and male FIL 5.4 ± 0.5 mm, *N* = 3, female FIL 7.5 mm, *N* = 1 vs. male FIIL 3.9 ± 0.4 mm, *N* = 3, female FIIL 6.2 mm, *N* = 1 in *I. yadera*); specifically also differs from *I. sarojamma*, *I. tysoni* and *I. yadera* by its head rounded in dorsal view (vs. nearly truncate in *I. sarojamma*, and sub-ovoid in *I. tysoni* and *I. yadera*); and further differs from *I. sarojamma* by its smaller adult size, male SVL 32.1 ± 1.9 mm, *N* = 3, female SVL 41.0 ± 3.2 mm, *N* = 6 (vs. larger, male SVL 36.4 ± 2.6 mm, *N* = 2, female SVL 61.2, *N* = 1) and head rounded in lateral view (vs. obtuse).

For differences with *I. beddomii* and *I. bhadrai* see 'Comparison' of those species. Detailed comparisons with all members of the proposed *Indirana beddomii* group, except *I. bhadrai*, was also provided by Dahanukar *et al*. [12].

Description of lectotype *(measurements in mm)*. Adult female (SVL 38.7); head longer than wide (HW 14.2, HL 14.7); snout rounded in dorsal and lateral view, its length (SL 6.0) longer than horizontal diameter of eye (EL 4.9); loreal region obtuse and concave with rounded canthus rostralis; interorbital space subequal (IUE 3.0) to upper eyelid (UEW 3.2); tympanum (TYD 2.6) 53% of eye diameter (EL 4.9). Forelimbs (FAL 7.2) shorter than hand length (HAL 8.5), finger length formula: IV<I<II<III, finger discs moderately wide compared to finger width (FD_I 1.1, FW_I 0.6; FD_{II} 1.3, FW_{II} 0.6; FD_{III} 1.4, FW_{III} 0.7; FD_{IV} 1.3, FW_{IV} 0.6). Thigh (TL 18.1) shorter than shank (SHL 21.1) and foot (FOL 20.2), toe discs wide compared to toe width (TD_I 1.2, TW_I 0.5; TD_{II} 1.4, TW_{II} 0.4; TD_{III} 1.5, TW_{III} 0.6; TD_{IV} 1.5, TW_{IV} 0.6; TD_V 1.2, TW_V 0.6), foot webbing: I1–2II1–2III1–3IV3–1V (Fig 5).

Dorsal skin sparsely granular with well developed discontinuous folds. Ventral surfaces of throat and chest smooth, abdomen and posterior parts of thigh granular (S4 Fig).

Colour of lectotype. *In preservation*. Dorsum light brown; margins of upper and lower jaw with alternate dark brown and cream coloured cross-bars (S4 Fig); a black band extending from the tip of the snout through the lower margin of eye, widening behind the eye and over the tympanum, and ending near the armpit on either sides of the head; tympanum blackish-brown; cream coloured mid-dorsal line extending from the snout up to the vent (S4 Fig). *In life.* See Figure 6, and the detailed description provided by Inger *et al*. [59] and Dahanukar *et al*. [12].

Secondary sexual character. *Male* (SDBDU 2015.2931), femoral glands absent (S1 Fig), nuptial pads well developed; *female* (NHM 74.4.29.1307 (ex BMNH 1947.2.27.92), large pigmented eggs (diameter 1.1–1.3 mm, *N* = 10).

Specimens examined. Males. Kerala: Palakkad district. SDBDU 2011.541, from Parambikulam, collected by SDB and team. Thiruvananthapuram district. SDBDU 2007.4329, from Athirimala, collected by SDB; SDBDU 2015.2931, from Chathankod–Bonnacaud, collected by SDB and SG. **Females.** Kerala: Idukki district. SDBDU 2012.814, from Munnar, collected by SDB. Kollam district. SDBDU 2011.280, from Pandimotta, collected by SDB and team. Thiruvananthapuram district. SDBDU 2015.2932, from Chathankod–Bonnacaud, collected by SDB and SG. Tamil Nadu: Coimbatore district. SDBDU 2011.549A, from Valparai, collected SDB and team. Tirunelveli district. SDBDU 2002.4091, from Kakkachi, collected by SDB. Colonial locality: Anamallays. NHM 1947.2.27.92 (74.4.29.1307), Col. Beddome.

Indirana leithii (Boulenger 1888)
Matheran Leaping Frog [12]
(Figs 1–6; S2–S3, S5 Figs; S1–S4 Tables)

Comparison. Within the *Indirana beddomi* group, *I. leithii* differs from all the members by its first finger shorter than second finger, male FIL 2.6 ± 0.2 mm, *N* = 3, female FIL 3.4 ± 0.1 mm, *N* = 5 vs. male FIIL 3.8 ± 0.2 mm, *N* = 3, female FIIL 4.5 ± 0.3 mm, *N* = 5 (vs. longer in *I. beddomii*, *I. sarojamma*, *I. tysoni* and *I. yadera*, and equal or nearly equal in *I. bhadrai* and *I. brachytarsus*) (Fig 4); specifically also differs from *I. sarojamma* by its smaller adult size, male SVL 27.2 ± 1.8 mm, *N* = 3, female SVL 34.4 ± 2.0 mm, *N* = 5 (vs. larger in *I. sarojamma*, male SVL 36.4 ± 2.6 mm, *N* = 2, female SVL 61.2 mm, *N* = 1), head rounded in dorsal view (vs. nearly truncate) and rounded in lateral view (vs. obtuse); and from *I. tysoni* by its head rounded in dorsal view (vs. sub-ovoid).

For more differences with *I. beddomii*, *I. bhadrai* and *I. brachytarsus* see 'Comparison' of those species. Detailed comparisons with all members of the proposed *Indirana beddomii* group, except *I. bhadrai*, was also provided by Dahanukar *et al*. [12].

Description of holotype. A general description was provided by Boulenger [8].

Specimens examined. Males. Maharashtra: Raigad district. SDBDU 2014.2515, from Matheran, collected by SDB and SG. Satara district. SDBDU 2002.2012, from Koyna, collected by SDB; SDBDU 2011.1095, from Bhimashankar, collected by SDB. **Females.** Maharashtra: Pune district. SDBDU 2011.1094 from Bhimashankar, collected by SDB. Raigad district. NHM 1947.2.28.17 (ex BMNH 1869.8.28.50), from "Matheran, Bombay", "single (female) specimen, presented by Dr. Leith"; SDBDU 2002.2010–11, from Matheran, collected by SDB; SDBDU 2014.2514, from Matheran, collected by SDB and SG.

Indirana sarojamma Dahanukar, Modak, Krutha, Nameer, Padhye and Molur 2016
Sarojamma's Leaping Frog [12]
(Figs 1–6; S2–S3, S5 Figs; S1–S4 Tables)

Comparison. Within the *Indirana beddomii* group, *I. sarojamma* differs from *I. tysoni* and *I. yadera* by its head nearly truncate in dorsal view (vs. sub-ovoid in both species) and head obtuse in lateral view (vs. rounded in in both species).

For more differences with *I. beddomii*, *I. bhadrai*, *I. brachytarsus* and *I. leithii* see 'Comparison' of those species. Detailed comparisons with all members of the proposed *Indirana beddomii* group, except *I. bhadrai*, was also provided by Dahanukar *et al*. [12].

Description of holotype. A detailed general description was published by Dahanukar *et al.* [12].

Secondary sexual character. *Male* (SDBDU 2013.2310), femoral glands absent, nuptial pads weakly developed.

Specimens examined. Males. Kerala state: Thiruvananthapuram district. SDBDU 2002.334 from Ponmudi, collected by SDB; SDBDU 2013.2310, from Ponmudi, collected by SDB and SG. **Females.** Kerala state: Thiruvananthapuram district. SDBDU 2002.516 from Chathankod–Bonnacaud, collected by SDB.

Indirana tysoni Dahanukar, Modak, Krutha, Nameer, Padhye and Molur 2016
Tyson's Leaping Frog [12]
(Figs 1–6; S2–S3, S6 Figs; S1–S4 Tables)

Comparison. Within the *Indirana beddomii* group, *Indirana tysoni* differs from *I. yadera* by its smaller adult snout-vent size, male: SVL 32.9 ± 1.0 mm, *N* = 4, female: SVL 52.1 ± 1.1 mm, *N* = 2 (vs. large, male: SVL 42.4 ± 2.8 mm, *N* = 3, female: SVL 58.6 mm, *N* = 1), loreal region obtuse (vs. acute) and absence of prominent spinules (vs. presence of sharp spinules on lateral sides of the abdomen and ventral margins of throat in adult males).

For more differences with *I. beddomii*, *I. bhadrai*, *I. brachytarsus*, *I. leithii* and *I. sarojamma* see 'Comparison' of those species. Detailed comparisons with all members of the proposed *Indirana beddomii* group, except *I. bhadrai*, was also provided by Dahanukar *et al.* [12].

Description of holotype. A detailed general description was published by Dahanukar *et al.* [12].

Secondary sexual characters. *Male* (SDBDU 2012.2228), femoral glands absent, nuptial pads well developed; *female* (SDBDU 2012.73), large pigmented eggs (diameter 1.2–1.6 mm, *N* = 10).

Variation. SDBDU 2012.2228: Dorsum with discontinuous folds, with irregular dark brown blotches, especially along the skin folds (Fig 6N).

Specimens examined. Males. Karnataka state: Kodagu district. SDBDU 2012.71, SDBDU 2012.74 and SDBDU 2012.2228, from Yavakapady, Coorg, collected by SDB and SG on 3 October 2012; SDBDU 2007.5093, an adult male, from Thalakaveri, collected by SDB on 15 August 2007. **Females.** Karnataka state: Kodagu district. SDBDU 2012.72b and SDBDU 2012.73, from Yavakapady, Coorg, collected by SDB and SG on 3 October 2012.

Indirana yadera Dahanukar, Modak, Krutha, Nameer, Padhye and Molur 2016
Yadera Leaping Frog [12]
(Figs 1–6; S2–S3, S6 Figs; S1–S4 Tables)

Comparison. Within the *Indirana beddomii* group, *I. yadera* differs from *I. bhadrai*, *I. brachytarsus* and *I. leithii* by its first finger longer than second finger, male FIL 5.4 ± 0.5 mm, *N* = 3, female FIL 7.5 mm, *N* = 1 vs. male FIIL 3.9 ± 0.4 mm, *N* = 3, female FIIL 6.2 mm, *N* = 1 (vs. equal or nearly equal in *I. bhadrai* and *I. brachytarsus*, and shorter in *I. leithii*); and differs from *I. beddomii* and *I. tysoni* its relatively large adult snout-vent size, male SVL 42.4 ± 2.8 mm, *N* = 3, female SVL 58.6 mm, *N* = 1 (vs. smaller in *I. beddomii* and *I. tysoni*).

For more differences with *I. beddomii*, *I. bhadrai*, *I. brachytarsus*, *I. leithii*, *I. sarojamma* and *I. tysoni* see 'Comparison' of those species. Detailed comparisons with all members of the proposed *Indirana beddomii* group, except *I. bhadrai*, was also provided by Dahanukar *et al.* [12].

For better clarity, we compare this species with other currently known species in this genus. *I. yadera* differs from members of the *Indirana semipalmata* group by its first finger longer than second finger (vs. equal or nearly equal) (Fig 4). Furthermore, this species differs from members of the genus *Sallywalkerana* by its larger adult snout-vent size (vs. smaller), first finger longer than second finger (vs. shorter), and fourth toe webbing above the second subarticular tubercle on either side, I1– 2II1–2^{-}III1–3^{-}IV3^{-}–1V (vs. just above the third subarticular tubercle) (Fig 5).

Description of holotype. A detailed general description was published by Dahanukar *et al.* [12].

Secondary sexual character. *Male* (SDBDU 2012.2744), femoral glands absent, nuptial pads present.

Variations. SDBDU 2015.2984 (male): dorsum reddish-brown, ventral surface light grey with dark grey reticulations, especially on throat (Fig 6P–Q); SDBDU 2015.3155 (female): dorsum light yellowish-brown, ventral surfaces of throat and belly greyish-white, thighs, hands and finger base brick red (Fig 6R).

Specimens examined. Males. Kerala state: Idukki district. SDBDU 2012.2744–45, two adult males, from Methooty, collected by SDB on 29 May 2012; SDBDU 2015.2984, an adult male, from Kozhikana, Gavi, collected by SDB and team on 2 July 2015. **Females.** Thrissur district. SDBDU 2015.3155, an adult female, from Vazhachal, collected by SDB and SG on 11 September 2015.

Indirana semipalmata **group**

Indirana chiravasi Padhye, Modak and Dahanukar 2014
Amboli Leaping Frog [10]
(Figs 1–5, 8; S2–S3, S7 Figs; S1–S4 Tables)

Comparison. Detailed comparisons with all members of the proposed *Indirana semipalmata* group, except *I. paramakri*, was provided by Padhye, Modak and Dahanukar [10], and Dahanukar *et al*. [12]. *Indirana chiravasi* differs from *I. paramakri* by its larger adult size (vs. smaller), loreal region obtuse (vs. acute), and webbing between first, second and third toe extending up to the disc on the outside, I1–2II1–2III1–3^{-}IV3^{-}–1V (vs. well below, I$1^{2/3}$– 2^{+}II$1^{3/4}$–$2^{3/4}$III2^{-}–3IV3–1^{+}V) (Fig 5).

Description of holotype. A detailed general description was published by Padhye Modak and Dahanukar [10].

Specimens examined. Males. Maharashtra: Sindhudurg district. SDBDU 2012.2112–13, SDBDU 2012.2125, SDBDU 2014.2483, from Amboli, collected by SDB and SG. Raigad district. SDBDU 2011.1448, from Phansad, collected by SDB and SG. **Females.** Maharashtra: Sindhudurg district. SDBDU 2008.01, from Amboli, collected by SDB; SDBDU 2012.2111, SDBDU 2012.2124, from Amboli, collected by SDB and SG; SDBDU 2015.3087, from Amboli, collected by SDB and SG.

Indirana duboisi Dahanukar, Modak, Krutha, Nameer, Padhye and Molur 2016
Dubois's Leaping Frog [12]
(Figs 1–5, 8; S2–S3, S7 Figs; S1–S4 Tables)

Comparison. A detailed comparison of *Indirana duboisi* with all members of the *Indirana semipalmata* group, except *I. paramakri*, was provided by Dahanukar *et al.* [12]. *Indirana duboisi* differs from *I. paramakri* by its snout nearly pointed in dorsal view (vs. sub-ovoid), snout rounded in lateral view (vs. nearly acute), loreal region obtuse (vs. acute), and webbing between first, second and third toes extending up to the disc on the outside, I1– 2II1–2III1–3^{-}IV3^{-}–1V (vs. well

below, $I1^{2/3}-2^{+}II1^{3/4}-2^{3/4}III2^{-}-3IV3-1^{+}V$) (Fig 5).

Description of holotype. A detailed general description was published by Dahanukar *et al.* [12].

Specimens examined. Males. Karnataka: Dakshin Kannada district. SDBDU 2003.1086, from Gundia–Kempholey, collected by SDB; Chikmagalur district. SDBDU 2011.510, from Bygoor, collected by SDB and team. Shimoga district. SDBDU 2014.2517, from Agumbe, collected by SDB. Uttara Kannada district. SDBDU 2003.40199, SDBDU 2003.40201, from Castle rock, collected by SDB; SDBDU 2011.1366, from Kathlekan, collected by SDB and team. **Females.** SDBDU 2011.1399, from Charmadi Ghats, collected by SDB and SG.

Indirana gundia (Dubois 1986)
Gundia Leaping Frog [12]
(Figs 1–5, 8; S1–S3, S8 Figs; S1–S4 Tables)

Comparison. A detailed comparison of *Indirana gundia* with all members of the *Indirana semipalmata* group, except *I. paramakri*, was provided by Dubois [9], Padhye, Modak and Dahanukar [10], and Dahanukar *et al.* [12]. *Indirana gundia* differs from *I. paramakri* by its snout rounded in dorsal view (vs. sub-ovoid), loreal region obtuse (vs. acute), and webbing between first, second and third toes extending up to the disc on the outside, $I1-2II1-2^{1/4}III1-3^{-}IV3^{-}-1V$ (vs. just above the first subarticular tubercle, $I1^{2/3}-2^{+}II1^{3/4}-2^{3/4}III2^{-}-3IV3-1^{+}V$) (Fig 5).

Description of holotype. A detailed general description was published by Dubois [9].

Secondary sexual character. *Male* (MNHN 1985.0633), femoral glands present (S1 Fig), nuptial pads weakly developed; *female* (SDBDU 2008.431), large pigmented eggs (diameter 1.1–1.2 mm, $N = 10$).

Specimens examined. Males. Karnataka: Dakshin Kannada district. MNHN 1985.0633, from Gundia. Hassan district. SDBDU 2008.433, from Kempholey, collected by SDB. Udupi district. SDBDU 2002.1114, from Kudremukh, collected by SDB. Kerala: Kannur district. SDBDU 2011.1057 and SDBDU 2011.1059, from Aralam, collected by SDB and team. **Females.** Karnataka: Dakshin Kannada district. MNHN 1985.0621, from Gundia. Kerala: Kannur district. SDBDU 2008.431–32, from Aralam, collected by SDB.

Indirana salelkari Modak, Dahanukar, Gosavi and Padhye 2015
Netravali Leaping Frog [11]
(Figs 1–5, 8; S2–S3, S8 Figs; S1–S4 Tables)

Comparison. A detailed comparison of *Indirana salelkari* with all members of the *Indirana semipalmata* group, except *I. paramakri*, was provided by Modak *et al.* [11] and Dahanukar *et al.* [12]. *Indirana salelkari* differs from *I. paramakri* by its snout rounded in dorsal view (vs. sub-ovoid), loreal region obtuse (vs. acute), and webbing between first, second and third toes extending up to the disc on the outside, $I1-2II1-2^{1/3}III1-3^{-}IV3^{-}-1V$ (vs. well below, $I1^{2/3}-2^{+}II1^{3/4}-2^{3/4}III2^{-}-3IV3-1^{+}V$) (Fig 5).

Description of holotype. A detailed general description was published in Modak *et al.* [11].

Specimens examined. Males. Karnataka: Uttara Kannada district. SDBDU 2011.1330, from Dandeli, collected by SDB and team.

Indirana semipalmata (Boulenger 1882)
Brown Leaping Frog [58]
(Figs 1–5, 8; S1–S3, S8 Figs; S1–S4 Tables)

Comparison. Within the *Indirana semipalmata* group, *I. semipalmata* differs from *I. chiravasi*, *I. duboisi, I. gundia* and *I. salelkari* by its first, second and third toe webbing up to the first subarticular tubercle on the outside, I2^{-}–2^{+}II2^{-}–3^{-}III2–3$^{1/4}$IV3$^{1/2}$–2V (vs. extending up to the disc in all four species) (Fig 5); specifically differs from *I. chiravasi* and *I. duboisi* by its snout rounded in dorsal view (vs. sub-ovoid in *I. chiravasi,* nearly pointed in *I. duboisi*); and further differs from *I. chiravasi* by its snout rounded in lateral view (vs. obtuse); differs from *I. paramakri* by its snout rounded in dorsal view (vs. sub-ovoid), fourth toe webbing below the second subarticluar tubercle on either side, I2^{-}–2^{+}II2^{-}–3^{-}III2–3$^{1/4}$IV3$^{1/2}$–2V (vs. up to the second subarticular tubercle, I1$^{2/3}$–2^{+}II1$^{3/4}$–2$^{3/4}$III2^{-}–3IV3–1^{+}V), and fifth toe webbing up to the first subarticular tubercle, I2^{-}–2^{+}II2^{-}–3^{-}III2–3$^{1/4}$IV3$^{1/2}$–2V (vs. well above, I1$^{2/3}$– 2^{+}II1$^{3/4}$–2$^{3/4}$III2^{-}–3IV3–1^{+}V) (Fig 5). A detailed comparison with all members of the *Indirana semipalmata* group, except *I. paramakri*, was also provided by Dahanukar *et al*. [12].

Description of lectotype *(measurements in mm)*. Adult female (SVL 34.8); head longer (HL 13.0) than its width (HW 12.6); snout rounded in dorsal and lateral view, its length (SL 5.1) longer than horizontal diameter of eye (EL 4.1); loreal region obtuse and concave; interorbital space (IUE 3.0) wider than upper eyelid (UEW 2.8) and narrower than internarial distance (IN 3.4); nostrils closer to tip of snout (NS 2.1) than eye (EN 2.6). Forelimbs (FAL 7.2) shorter than hand length (HAL 8.2), finger lengths formula: IV<II<I<III, finger discs moderately wide compared to finger width (FD_{I} 1.0, FW_{I} 0.6; FD_{II} 1.1, FW_{II} 0.7; FD_{III} 1.6, FW_{III} 0.7; FD_{IV} 1.1, FW_{IV} 0.6). Thigh length (TL 18.6) subequal to shank (SHL 18.5), and longer than foot (FOL 17.2), toe discs wide compared to toe width (TD_{I} 0.9, TW_{I} 0.4; TD_{II} 1.1, TW_{II} 0.5; TD_{III} 1.0, TW_{III} 0.5; TD_{IV} 1.2, TW_{IV} 0.6; TD_{V} 1.1, TW_{V} 0.5), foot webbing: I2^{-}–2^{+}II2^{-}–3^{-}III2–3$^{1/4}$IV3$^{1/2}$–2V.

Dorsal skin sparsely granular with short discontinuous folds. Ventral surfaces of throat and chest smooth, and abdomen and posterior parts of thigh granular.

Colour of lectotype. *In preservation*. Dorsum light brown with dark brown spots; snout lighter in colour than dorsum; margins of upper and lower jaw with alternate dark brown and cream coloured cross-bars (S8 Fig); a dark brown band extending from the tip of snout through the lower margin of eye, widening behind the eye and over the tympanum, and ending near the armpit on either sides of the head; tympanum dark brown. *In life* (SDBDU 2015.3033). Dorsum yellowish-brown with irregular dark brown blotches especially along the dorsal skin folds; snout yellowish-brown; margins of upper and lower jaw with alternate greyish-brown and light yellow coloured cross-bars; space between tympanum and eye dark brown; tympanum light reddish-brown; forelimbs (including fingers) and hindlimbs (including toes) light brown with dark brown transverse bands; anterior and posterior parts of flank light yellowish-grey. Ventral surfaces light greyish-brown (Fig 8).

Secondary sexual character. *Male* (SDBDU 2015.3034), femoral glands present (S1 Fig), nuptial pads weakly developed; *female* [NHM 74.4.29.605 (ex BNHS 1947.2.29.50)], large pigmented eggs (diameter 1.4–1.7 mm, N = 10).

Variation. SDBDU 2015.3014: Dorsum reddish-brown with faint irregular brown coloured blotches, margins of upper and lower jaw with alternate blackish-brown and cream coloured cross-

bars; a dark blackish-brown band extending from the nostril through the lower margin of eye, widening behind the eye and over the tympanum, and ending near the armpit on either sides of the head; limbs light reddish-brown with faint brown transverse bands (Fig 8).

Specimens examined. Males. Kerala: Idukki district. SDBDU 2008.443, SDBDU 2008.904, from Double Cut, Kattappana, collected by SDB; SDBDU 2010.201 from Kulamavu, collected by SDB. Palakkad district. SDBDU 2002.522, from Nelliampathy, Pakuthipaalam, collected by SDB; SDBDU 2005.26, from Nelliyampathy, Kaikatti, collected by SDB; SDBDU 2015.3014 from Siruvani, Singappara, collected by SDB and SG; SDBDU 2015.3034, from Parambikulam TR, collected by SDB and SG. Pathanamthitta district. SDBDU 2006.4819, from Gavi, collected by SDB. Thiruvananthapuram district. SDBDU 2002.520, from Chathankod–Bonnacaud, collected by SDB. **Females.** Kerala: Thiruvananthapuram district. SDBDU 2006.4773A and SDBDU 2006.4773B, from Kallar, Ponmudi, collected by SDB. Colonial locality: Malabar. NHM 74.4.29.605 (ex BNHS 1947.2.29.50).

Genus *Sallywalkerana*

Sallywalkerana diplosticta (Günther 1876)
Spotted Leaping Frog [58]
(Figs 1–5, 9; S2–S3, S9 Figs; S1–S4 Tables)

Comparison. *Sallywalkerana diplosticta* differs from *S. leptodactyla* and *S. phrynoderma* by its smaller adult size, male: SVL 22.8–28.4 mm, *N* = 4, female: SVL 27.7–28.2 mm, *N* = 3 (vs. larger, male: SVL 26.5–29.6 mm, *N* = 3, female: SVL 34.1–38.8 mm, *N* = 4; male: SVL 31.5–33.2 mm, *N* = 2, female: SVL 33.9 mm, *N* = 1, respectively), black coloured sharp nuptial spines on medial surface of the first finger (Fig 4) (vs. absent in both species), greyish-black spots present on lateral surfaces and two dark spots on posterior side of the dorsum, near the groin (Fig 9) (vs. absence of dark spots on lateral surfaces and posterior side of the dorsum, in both species). More specifically, differs from *S. leptodactyla* by its first toe webbing just above the first subarticular tubercle, $\mathrm{I}2^{-}$–$2\mathrm{II}2$–$3\mathrm{III}3^{-}$–$4^{-}\mathrm{IV}3^{2}/_{3}$–$2^{1}/_{5}\mathrm{V}$ (vs. up to the disc, $\mathrm{I}1$–$2\mathrm{II}2$–$3\mathrm{III}3^{-}$–$4^{-}\mathrm{IV}3^{3}/_{4}$–$2\mathrm{V}$); and from *S. phrynoderma* by its fifth toe webbing extending well beyond the second subarticular tubercle, $\mathrm{I}2^{-}$–$2\mathrm{II}2$–$3\mathrm{III}3^{-}$–$4^{-}\mathrm{IV}3^{2}/_{3}$–$2^{1}/_{5}\mathrm{V}$ (vs. just above the second subarticular tubercle, $\mathrm{I}2^{-}$–$2\mathrm{II}2$–$3\mathrm{III}3^{-}$–$4^{-}\mathrm{IV}4^{-}$–$3^{-}\mathrm{V}$) (Fig 5). A detailed comparison with all members of the genus *Sallywalkerana* was also provided by Dahanukar *et al.* [12].

Description of lectotype *(measurements in mm).* Adult female (SVL 27.7); head length (HL 10.8) subequal to head width (HW 10.9); snout sub-ovoid in dorsal view, its length (SL 3.9) longer than horizontal diameter of eye (EL 3.3); loreal region acute with indistinct canthus rostralis; interorbital space (IUE 2.6) wider than upper eyelid (UEW 2.0); tympanum (TYD 1.6) 48% of eye diameter (EL 3.3). Forelimbs (FAL 5.6) shorter than hand length (HAL 6.9), finger length formula: I<IV<II<III, finger discs slightly wider compared to finger width (FD_{I} 0.6, FW_{I} 0.3; FD_{II} 0.7, FW_{II} 0.4; FD_{III} 0.9, FW_{III} 0.5; FD_{IV} 0.9, FW_{IV} 0.4). Thigh (TL 13.9) shorter than shank (SHL 15.3), and nearly equal to foot (FOL 13.6), toe discs slightly wider compared to toe width (TD_{I} 0.8, TW_{I} 0.4; TD_{II} 1.1, TW_{II} 0.5; TD_{III} 1.2, TW_{III} 0.5; TD_{IV} 1.2, TW_{IV} 0.4; TD_{V} 1.1, TW_{V} 0.5), foot webbing: $\mathrm{I}2^{-}$–$2\mathrm{II}2$–$3\mathrm{III}3^{-}$–$4^{-}\mathrm{IV}3^{2}/_{3}$–$2^{1}/_{5}\mathrm{V}$ (Fig 5).

Dorsal skin shagreened with faintly developed discontinuous folds; limbs sparsely granular. Ventral surfaces of throat, chest, abdomen and posterior parts of thigh, smooth.

Colour of lectotype. *In preservation*. Dorsum light brown; margins of upper and lower jaw with alternate dark brown and cream coloured cross-bars (S9 Fig); lateral sides of head greyish-black; dark brown spots present on lateral surfaces and two dark brown spots on posterier side of the dorsum, near the groin (S9 Fig). *In life*. See Figure 9 for colouration, and the detailed description provided by Dahanukar *et al*. [12].

Secondary sexual character. *Male* (SDBDU 2015.2957), femoral glands absent, sharp nuptial spines present on medial surface of the first finger (Fig 4); *female* (SDBDU 2002.1249), large pigmented eggs (diameter 1.3–1.5 mm, *N* = 10).

Specimens examined. Males. Kerala: Thiruvananthapuram district. SDBDU 2001.123, from Ponmudi, collected by SDB; SDBDU 2003.40103, from Athirimala, collected by SDB; SDBDU 2015.2956–57, from Pandipath, collected by SDB and SG. **Females.** Kerala: Thiruvananthapuram district. SDBDU 2002.513 and SDBDU 2002.1249, from Athirimala, collected by SDB. Colonial locality: "Malabar". NHM 1874.4.29.1412 (ex BMNH 1947.2.2.21).

Sallywalkerana leptodactyla (Boulenger 1882)
Slender-toed Leaping Frog [12]
(Figs 1–5, 9; S1–S3, S9 Figs; S1–S4 Tables)

Comparison. *Sallywalkerana leptodactyla* differs from *S. phrynoderma* by its dorsal skin shagreened to sparsely granular (vs. prominently granular and warty), and fifth toe webbing extending up to the first subarticular tubercle, I1–2II2–3III3^{-}–4^{-}IV3^{3}/$_{4}$–2V (vs. just above the second subarticular tubercle, I2^{-}–2II2–3III3^{-}–4^{-}IV4^{-}–3^{-}V) (Fig 5).

For differences with *S. diplosticta*, see 'Comparison' of that species. A detailed comparison with all members of the genus *Sallywalkerana* was also provided by Dahanukar *et al*. [12].

Description of lectotype *(measurements in mm)*. Adult female (SVL 35.3); head wider (HW 14.0) than long (HL 13.6); snout rounded in dorsal view, its length (SL 5.5) longer than horizontal diameter of eye (EL 4.4); loreal region obtuse with rounded canthus rostralis; interorbital space (IUE 3.6) wider than upper eyelid (UEW 2.2); tympanum (TYD 2.4) 55% of eye diameter (EL 4.4). Forelimbs (FAL 6.7) shorter than hand length (HAL 9.3), finger length formula: I<IV<II<III, finger discs slightly wider compared to finger width (FD_{I} 0.9, FW_{I} 0.5; FD_{II} 1.2, FW_{II} 0.6; FD_{III} 1.4, FW_{III} 0.5; FD_{IV} 1.5, FW_{IV} 0.9). Thigh (TL 18.5) shorter than shank (SHL 21.2) and foot (FOL 19.5), toe discs slightly wider compared to toe width (TD_{I} 1.1, TW_{I} 0.5; TD_{II} 1.4, TW_{II} 0.6; TD_{III} 1.4, TW_{III} 0.6; TD_{IV} 1.5, TW_{IV} 0.6; TD_{V} 1.2, TW_{V} 0.5), foot webbing: I1–2II2–3III3^{-}–4^{-}IV3^{3}/$_{4}$–2V (Fig 5).

Dorsal skin shagreened to granular with faintly developed discontinuous folds, limbs sparsely granular. Ventral surfaces of throat, chest, abdomen and posterior parts of thigh, smooth.

Colour of lectotype. *In preservation*. Dorsum light brown; margins of upper and lower jaw with alternate dark brown and cream coloured cross-bars (S9 Fig). *In life*. See Figure 9 and the below 'variation' section, and the detailed description provided by Dahanukar *et al*. [12].

Secondary sexual characters. *Male* (SDBDU 2003.40336), femoral glands absent (S1 Fig); *Female* (SDBDU 2002.917), large pigmented eggs (diameter 1.3–1.4 mm, *N* = 20).

Variations. Dorsal colour is variable. SDBDU 2013.911 (female): reddish brown dorsum

with prominent brown cross bands on limbs (Fig 9E); SDBDU 2002.917 (female): dorsum bluish-brown with a mid dorsal line extending from the tip of snout up to the vent (Fig 9F); SDBDU 2004.40336 (male): dorsum light brown with scattered dark brown spots (Fig 9G).

Specimens examined. Males. Kerala: Idukki district. SDBDU 2002.1026, from Anamudi, collected by SDB; SDBDU 2011.1058A, from Eravikulam NP, collected by SDB and team. Tamil Nadu: Coimbatore district. SDBDU 2004.40336, Valparai, Andiparai shola, collected by SDB. **Females.** Kerala: Idukki district. SDBDU 2013.911, from Mattupetti, collected by SDB and team. Tamil Nadu: Dindigul district. SDBDU 2002.916–17, from Kodaikanal, collected by SDB. Colonial locality: "Malabar". NHM 1874.4.29.593 (ex BMNH 1947.2.29.39).

***Sallywalkerana phrynoderma* (Boulenger 1882)**
Warty-skinned Leaping Frog [12]
(Figs 1–5, 9; S1–S3, S9 Figs; S1–S4 Tables)

Comparison. *Sallywalkerana phrynoderma* differs from *S. diplosticta* and *S. leptodactyla* by its prominently granular warts on dorsal skin. For more differences see 'Comparison' of those species. A detailed comparison with all members of the genus *Sallywalkerana* was also provided by Dahanukar *et al*. [12].

Description of lectotype *(measurements in mm).* Adult female (SVL 33.9); head wider (HW 13.7) than long (HL 13.1); snout rounded in dorsal view, its length (SL 5.3) longer than horizontal diameter of eye (EL 4.0); loreal region obtuse with indistinct canthus rostralis; interorbital space (IUE 2.9) smaller than upper eyelid (UEW 3.3); tympanum (TYD 2.1) 53% of eye diameter (EL 4.0). Forelimbs (FAL 6.2) shorter than hand length (HAL 8.3), finger length formula: I=IV<II<III, finger discs slightly wider compared to finger width (FD_{I} 1.0, FW_{I} 0.5; FD_{II} 1.2, FW_{II} 0.5; FD_{III} 1.2, FW_{III} 0.6; FD_{IV} 1.2, FW_{IV} 0.6). Thigh (TL 18.4) shorter than shank (SHL 20.1), and longer than foot (FOL 17.8), toe discs slightly wider compared to toe width (TD_{I} 1.0, TW_{I} 0.6; TD_{II} 1.2, TW_{II} 0.6; TD_{III} 1.4, TW_{III} 0.6; TD_{IV} 1.4, TW_{IV} 0.6; TD_{V} 1.1, TW_{V} 0.6), foot webbing: $I2^{-}–2II2–3III3^{-}–4^{-}IV4^{-}–3^{-}V$ (Fig 5).

Dorsal skin covered with prominent glandular warts. Ventral surfaces of throat, chest, and abdomen, shagreened, and posterior parts of thigh, sparsely granular.

Colour of lectotype. *In preservation*. Dorsum greyish-brown; margins of upper and lower jaw with alternate dark brown and cream coloured cross-bars (S9 Fig). *In life* (SDBDU 2002.1181) Dorsum light brown; lateral surface with black patches; dark grey cross bands on limbs; tympanum black (Figs 9H, I). Detailed description was also provided by Dahanukar *et al.* [12].

Secondary sexual character. *Male* (SDBDU 2002.1181), femoral glands absent (S1 Fig).

Specimens examined. Males. Tamil Nadu: Coimbatore district. SDBDU 2002.1181–82, from Grass Hills, Akkamalai shola, collected by SDB. **Female.** Colonial locality: "Anamallays". NHM 82.2.10.21 (ex BMNH 1947.2.3.8).
