# Supplementary material for: Molecular and Morphological Study of Leaping Frogs (Anura, Ranixalidae) with Description of Two New Species
Source: PLoS One. 2016 Nov 16;11(11):e0166326. doi: 10.1371/journal.pone.0166326 (PMC5112961; doi:10.1371/journal.pone.0166326)
Supplement: S1 Table — Localities are arranged by State. (PDF) [file pone.0166326.s011.pdf]

Supporting information: Tables

Molecular and morphological study of Leaping frogs (Anura, Ranixalidae) with description of two new species

Sonali Garg, SD Biju

S1 Table. Collection localities of ranixalid species reported in this study. Localities are arranged by State.

Locality	Altitude	Coordinates		Species recorded
	(meters)	Latitude (°N)	Longitude (°E)	
INDIA				
**Tamil Nadu**				
Coimbatore district				
Grass Hills	1800	10.3167	77.0667	*S. leptodactyla*
Grass Hills, Akkamalai shola	1750	10.3415	77.0212	*S. phrynoderma*
Valparai	1480	10.3887	76.9964	*S. leptodactyla*
Valparai, Andiparai shola	1228	10.3678	76.9672	*I. brachytarsus, S. leptodactyla*
Top Slip, Karian Shola	775	10.4667	76.9167	*I. semipalmata*
Dindigul district				
Kodaikanal	1780	10.2333	77.4833	*S. leptodactyla*
Kanyakumari district				
Glenback Estate, Kiriparai	450	08.4188	77.4182	*I. brachytarsus*
Tirunelveli district				
Kakkachi	1266	08.5488	77.3904	*I. brachytarsus*
Kakkachi	1200	08.6667	77.6333	*I. brachytarsus*
**Kerala**				
Ernakulam district				
Neriamangalam	205	10.0646	76.7868	*I. semipalmata*, *I. yadera*
Idukki district				
Eravikulam NP, Anamudi	2310	10.1750	77.0697	*S. leptodactyla*
Eravikulam NP	2105	10.1846	77.0900	*S. leptodactyla*
Eravikulam NP, Vagvarai	1798	10.1753	77.0948	*S. leptodactyla*
Kattapana, Double cut	1082	09.8282	77.0175	*I. semipalmata*
Kulamav	440	09.8444	76.8722	*I. semipalmata*
Munnar, Kadalar	1429	10.1311	77.0005	*I. brachytarsus*
Munnar	1450	10.0847	77.0586	*S. leptodactyla*
Mattupetti	1578	10.1042	77.1211	*S. leptodactyla*
Pampadumpara	1025	09.8010	77.1540	*I. semipalmata*
Methooty	443	09.8416	76.8704	*I. brachytarsus*, *I. yadera*
Anchuruli, Periyar TR	929	09.5873	77.1520	*I. brachytarsus*
Thekkady, Periyar TR	980	09.5803	77.1593	*I. brachytarsus*, *I. semipalmata*
Upper Manalar, Periyar TR	1508	09.5958	77.3456	*I. brachytarsus*
Kannur district				
Aralam WLS	66	11.9221	75.7913	*I. gundia*
Aralam WLS, Meenmutty	358	11.9384	75.8573	*I. gundia*
Kollam district				
Kattilappara, Shendurney WLS	130	08.9179	77.0953	*I. brachytarsus*
Pandimotta, Shendurney WLS	741	08.8502	77.1801	*S. diplosticta*
Pandimotta, Shendurney WLS	1222	08.8271	77.2165	*I. brachytarsus*
Kottayam district				
Valanjamkanam falls	875	08.5629	76.9778	*I. brachytarsus*
Kozhikode district				
Kakkayam	752	11.5542	75.9196	*I. beddomii*
Palakkad district				

Nelliyampathy, Kaikatti	965	10.5373	76.6764	*I. brachytarsus*, *I. semipalmata*
Nelliyampathy, Kesavapara	923	10.5243	76.6673	*I. brachytarsus*
Nelliyampathy, Pakuthipaalam	980	10.4797	76.6736	*I. semipalmata*
Nenmara	648	10.5289	76.6660	*I. brachytarsus*, *I. semipalmata*
Parambikulam TR	544	10.4453	76.8130	*I. brachytarsus*
Parambikulam TR	546	10.4372	76.7801	*I. semipalmata*
Parambikulam TR, Poopara	955	10.3516	76.8215	*I. brachytarsus*
Sairandhri, Silent Valley	962	11.0949	76.4505	*I. beddomii*
Siruvani, Kuddam	826	10.9795	76.6151	*I. beddomii*
Siruvani, Pattiar	908	10.9696	76.6545	*I. beddomii*
Siruvani, Singappara	856	10.9798	76.6150	*I. semipalmata*
Pathanamthitta district				
Gavi	1107	09.4192	77.1517	*I. brachytarsus*, *I. semipalmata*
Kozhikana, Periyar TR	947	09.4901	77.1363	*I. yadera*
Nilakkal	112	09.3724	76.9898	*I. yadera*
Thiruvananthapuram district				
Athirimala	993	08.6178	77.2297	*I. brachytarsus*, *S. diplosticta*
Athirimala	1425	08.6238	77.2454	*S. diplosticta*
Chathankod	140	08.6593	77.1494	*I. brachytarsus*
Chathankod–Bonnacaud	488	08.6737	77.1575	*I. brachytarsus*, *I. sarojamma*, *I. semipalmata*
Kallar	140	08.7108	77.1293	*I. semipalmata*
Ponmudi	1014	08.7682	77.1101	*I. brachytarsus*, *I. sarojamma*, *S. diplosticta*
Ponkalapara	1370	08.6247	77.2425	*I. brachytarsus*, *S. leptodactyla*
Pandipath	1308	08.6810	77.1924	*S. diplosticta*
Thrissur district				
Vazhachal	533	10.2814	76.6904	*I. brachytarsus*, *I. yadera*
Vazhachal	577	10.2838	76.6851	*I. brachytarsus*
Peechi-Vazhani WLS	460	10.5032	76.4653	*I. brachytarsus*
Wayanad district				
Settukunnu	823	11.6172	75.9913	*I. beddomii*, *I. paramakri*
Suganthagiri	852	11.5386	76.0539	*I. beddomii*, *I. paramakri*
**Karnataka**				
Chikmagalur district				
Bygoor	1042	13.3111	75.6128	*I. duboisi*
Charmadi Ghats	489	13.0753	75.4552	*I. duboisi*
Charmadi Ghats	608	13.0798	75.4650	*I. duboisi*
Charmadi Ghats	746	13.0982	75.4793	*I. duboisi*
Charmadi Ghats	826	13.1113	75.4883	*I. tysoni*
Muthodi, Bhadra WLS	1105	13.3688	75.6566	*I. bhadrai*
Dakshin Kannada district				
Gundia	130	12.8280	75.5715	*I. gundia*
Gundia	128	12.8250	75.5690	*I. gundia*
Gundia	224	12.8290	75.6070	*I. gundia*
Gundia–Kempholey	650	12.8567	75.6955	*I. duboisi*
Hassan district				
Kempholey	800	12.8706	75.7053	*I. duboisi*
Kempholey	880	12.8567	75.6955	*I. gundia*
Kottigehara	798	13.1230	75.4954	*I. duboisi*
Kodagu district				
Monnangeri	250	12.4696	75.6094	*I. gundia*
Bhagamandala	889	12.3856	75.5343	*I. tysoni*
Madikeri, Abby falls	1085	12.4568	75.7106	*I. tysoni*
Thalakaveri	1183	12.3857	75.5026	*I. tysoni*
Yavakapady, Coorg	1176	12.2201	75.6557	*I. tysoni*
Shimoga district				
Agumbe	670	13.5170	75.0883	*I. duboisi*
Jog falls	600	13.4421	75.1704	*I. salelkari*

Udupi district				
Kudremukh	745	13.2853	75.1420	*I. gundia*
Uttara Kannada district				
Castle rock	577	15.5217	74.3823	*I. duboisi*
Dandeli	494	15.1669	74.6323	*I. salelkari*
Kathlekan	554	14.2739	74.7469	*I. duboisi*
Unchali falls	444	14.4091	74.7468	*I. salelkari*
**Maharashtra**				
Pune district				
Bhimashankar	972	19.0681	73.6047	*I. leithii*
Raigad district				
Matheran	749	18.9800	73.2700	*I. leithii*
Matheran	819	18.9869	73.2708	*I. leithii*
Phansad	221	18.4551	72.9262	*I. chiravasi*
Satara district				
Humbarli, Koyna	827	17.4033	73.7367	*I. chiravasi*
Koyna Nagar	941	17.4163	73.7252	*I. chiravasi*
Koyna Nagar	998	17.4162	73.7294	*I. chiravasi*
Mahabaleshwar, Dhobi falls	1235	17.9172	73.6536	*I. leithii*
Sindhudurg district				
Amboli	719	15.9599	73.9973	*I. chiravasi*
Amboli	733	15.9691	73.9882	*I. chiravasi*
Amboli	744	15.9560	73.9970	*I. chiravasi*
