# Supplementary material for: Molecular and Morphological Study of Leaping Frogs (Anura, Ranixalidae) with Description of Two New Species
Source: PLoS One. 2016 Nov 16;11(11):e0166326. doi: 10.1371/journal.pone.0166326 (PMC5112961; doi:10.1371/journal.pone.0166326)
Supplement: S2 Table — (PDF) [file pone.0166326.s012.pdf]

Supporting information: Tables

Molecular and morphological study of Leaping frogs (Anura, Ranixalidae) with description of two new species

Sonali Garg, SD Biju

S2 Table. List of DNA sequences included in the study.

Family/Species	Collection Locality	Voucher No.	Accession nos.	Reference
**Ranixalidae**				
Indirana beddomii	Kakkayam, Kerala	SDBDU 2010.225	KX966028	This study
	Sairandhri, Silent Valley, Kerala	SDBDU 2011.958	KX966029	This study
	Sairandhri, Silent Valley, Kerala	SDBDU 2011.960	KX966030, KX966163	This study
	Settukkunu, Wayanad, Kerala	SDBDU 2011.850	KX966031	This study
	Siruvani, Kuddam, Kerala	SDBDU 2011.1269	KX966032	This study
	Siruvani, Kuddam, Kerala	SDBDU 2011.1270	KX966033	This study
	Siruvani, Pattiar, Kerala	SDBDU 2011.1256	KX966034	This study
	Suganthagiri, Wayanad, Kerala	SDBDU 2005.1232	KX966035	This study
	Peruvannamuzhi	WILD-14-AMP-411	KX641759	Dahanukar *et al*. 2016
	Peruvannamuzhi	WILD-14-AMP-414	KX641760	Dahanukar *et al*. 2016
	Peruvannamuzhi	WILD-14-AMP-420	KX641761	Dahanukar *et al*. 2016
	Kunthipuzha	WILD-14-AMP-421	KX641762	Dahanukar *et al*. 2016
	Aralam	WILD-13-AMP-138	KX641763	Dahanukar *et al*. 2016
	Sairandhri	WILD-14-AMP-409	KX641764	Dahanukar *et al*. 2016
	Kakkayam	WILD-14-AMP-413	KX641765	Dahanukar *et al*. 2016
	–	AA87	JQ596658	Nair *et al*. 2012
	–	AA88	JQ596659	Nair *et al*. 2012
	–	AA89	JQ596660	Nair *et al*. 2012
	–	AA95	JQ596661	Nair *et al*. 2012
	–	AA99	JQ596662	Nair *et al*. 2012
	Ooty	–	AF215392	Vences 2000
	India	IN1 5411	KF991280	Barej *et al*. 2014
Indirana bhadrai sp. nov.	Muthodi, Bhadra WLS, Karnataka	ZSI/WGRC/V/A887	KX966036, KX966164	This study
Indirana brachytarsus	Anchurulli, Periyar TR, Kerala	SDBDU 2006.4800	KX966037	This study
	Anchurulli, Periyar TR, Kerala	SDBDU 2009.2513	KX966038	This study
	Athirimala, Kerala	SDBDU 2007.4323	KX966039	This study
	Bonnacaud, Kerala	SDBDU 2014.2662	KX966040	This study
	Chathankod, Kerala	SDBDU 2005.1231	KX966041	This study
	Chathankod–Bonnacaud, Kerala	SDBDU 2015.2931	KX966042	This study
	Chathankod–Bonnacaud, Kerala	SDBDU 2015.2932	KX966043	This study
	Gavi, Kerala	SDBDU 2006.4867	KX966044	This study
	Gavi, Kerala	SDBDU 2009.2501	KX966045	This study
	Kadalar, Munnar, Kerala	SDBDU 2011.1046	KX966046	This study
	Kadalar, Munnar, Kerala	SDBDU 2012.814	KX966047	This study
	Kadalar, Munnar, Kerala	SDBDU 2012.870	KX966048	This study
	Kaikatti, Nelliyampathy, Kerala	SDBDU 2010.254	KX966049	This study
	Kattilappara, Shendurney WLS, Kerala	SDBDU 2011.276	KX966050	This study
	Kesavapara, Nelliyampathy, Kerala	SDBDU 2011.1181	KX966051	This study
	Kesavapara, Nelliyampathy, Kerala	SDBDU 2011.1182	KX966052	This study
	Kesavapara, Nelliyampathy,	SDBDU 2011.1183	KX966053	This study

	Kerala			
	Valanjamkanam falls, Kerala	SDBDU 2008.329	KX966054	This study
	Upper Manalar, Periyar TR, Kerala	SDBDU 2015.2995	KX966055	This study
	Methooty, Kerala	SDBDU 2011.2731	KX966056	This study
	Methooty, Kerala	SDBDU 2011.2741	KX966057	This study
	Methooty, Kerala	SDBDU 2011.2747	KX966058	This study
	Methooty, Kerala	SDBDU 2013.2287	KX966059	This study
	Methooty, Kerala	SDBDU 2014.2686	KX966060	This study
	Nenmara, Kerala	SDBDU 2011.1132	KX966061	This study
	Pandimotta, Shendurney WLS, Kerala	SDBDU 2011.280	KX966062	This study
	Pandimotta, Shendurney WLS, Kerala	SDBDU 2011.281	KX966063	This study
	Parambikulam TR, Kerala	SDBDU 2011.541	KX966064	This study
	Peechi-Vazhani WLS, Kerala	SDBDU 2005.1251	KX966065	This study
	Ponkalapara, Kerala	SDBDU 2007.4327	KX966066	This study
	Ponkalapara, Kerala	SDBDU 2007.4329	KX966067	This study
	Ponmudi, Kerala	SDBDU 2008.1776	KX966068	This study
	Poopara, Parambikulam TR, Kerala	SDBDU 2011.549	KX966069	This study
	Thekkady, Periyar TR, Kerala	SDBDU 2012.1825	KX966070	This study
	Thekkady, Periyar TR, Kerala	SDBDU 2012.1828	KX966071	This study
	Vazhachal, Kerala	SDBDU 2015.3156	KX966072	This study
	Vazhachal, Kerala	SDBDU 2015.3160	KX966073	This study
	Glenback estate, Kiriparai, Tamil Nadu	SDBDU 2008.1936	KX966074	This study
	Kakkachi, Tamil Nadu	SDBDU 2008.2014	KX966075	This study
	Kakkachi, Tamil Nadu	SDBDU 2002.4091	KX966076	This study
	Valparai, Andiparai shola, Tamil Nadu	SDBDU 2011.549A	KX966077, KX966165	This study
	–	AA72	JQ596642	Nair *et al*. 2012
	–	AA75	JQ596643	Nair *et al*. 2012
	–	AA77	JQ596644	Nair *et al*. 2012
	–	AA23	JQ596645	Nair *et al*. 2012
	–	AA71	JQ596646	Nair *et al*. 2012
	–	AA638	JQ596647	Nair *et al*. 2012
	India	IN-2 isolate 5412	KF991281	Barej *et al*. 2014
	India	KR-2003	AY322298	Roelants *et al*. 2004
	Neyyar	WILD-13-AMP-234	KX641766	Dahanukar *et al*. 2016
	Ponmudi	WILD-13-AMP-301	KX641767	Dahanukar *et al*. 2016
	Painavu	WILD-14-AMP-358	KX641768	Dahanukar *et al*. 2016
	Vellakkamaly	WILD-14-AMP-437	KX641769	Dahanukar *et al*. 2016
	Chimmony	WILD-14-AMP-475	KX641770	Dahanukar *et al*. 2016
	Chimmony	WILD-14-AMP-477	KX641771	Dahanukar *et al*. 2016
	Peechi-Vazhani	WILD-14-AMP-478	KX641772	Dahanukar *et al*. 2016
	Topslip	WILD-15-AMP-609	KX641773	Dahanukar *et al*. 2016
	Ponmudi	WILD-13-AMP-241	KX641774	Dahanukar *et al*. 2016
	Neyyar	WILD-13-AMP-247	KX641775	Dahanukar *et al*. 2016
	Ponmudi	WILD-13-AMP-285	KX641776	Dahanukar *et al*. 2016
	Ponmudi	WILD-13-AMP-293	KX641777	Dahanukar *et al*. 2016
	Painavu	WILD-14-AMP-359	KX641778	Dahanukar *et al*. 2016
	Vellakkamaly	WILD-14-AMP-442	KX641779	Dahanukar *et al*. 2016
	Kodaikanal	–	AF215391	Vences 2000
Indirana chiravasi	Amboli, Maharashtra	SDBDU 2004.4511	KX966078	This study
	Amboli, Maharashtra	SDBDU 2006.1431	KX966079	This study
	Amboli, Maharashtra	SDBDU 2012.2112	KX966080, KX966166	This study
	Amboli, Maharashtra	SDBDU 2012.2114	KX966081	This study

	Amboli, Maharashtra	SDBDU 2012.2124	KX966082	This study
	Amboli, Maharashtra	SDBDU 2014.2483	KX966083	This study
	Amboli, Maharashtra	SDBDU 2015.3086	KX966084	This study
	Amboli, Maharashtra	BNHS 5890	KM386531	Padhye *et al*. 2014
	Amboli, Maharashtra	WILD-14-AMP-489	KM386530	Padhye *et al*. 2014
	Koyna, Maharashtra	SDBDU 2004.4506	KX966085	This study
	Koyna, Maharashtra	SDBDU 2006.1420	KX966086	This study
	Koyna, Maharashtra	SDBDU 2007.6046	KX966087	This study
	Koyna, Maharashtra	SDBDU 2010.372	KX966088	This study
	Phansad, Maharashtra	SDBDU 2011.1448	KX966089	This study
	Koyna	WILD-15-AMP-530	KX641780	Dahanukar *et al*. 2016
	Chandoli	WILD-15-AMP-535	KX641781	Dahanukar *et al*. 2016
	Kitawade	WILD-15-AMP-612	KX641782	Dahanukar *et al*. 2016
Indirana duboisi	Agumbe, Karnataka	SDBDU 2014.2516	KX966090	This study
	Bygoor, Karnataka	SDBDU 2011.510	KX966091	This study
	Charmadi Ghat, Karnataka	SDBDU 2011.519	KX966092	This study
	Charmadi Ghats, Karnataka	SDBDU 2011.1398	KX966093	This study
	Charmadi Ghats, Karnataka	SDBDU 2011.1399	KX966094	This study
	Gundia-Kempholey, Karnataka	SDBDU 2003.1086	KX966095, KX966167	This study
	Kathlekan, Karnataka	SDBDU 2011.1366	KX966096	This study
	Kempholey, Karnataka	SDBDU 2011.51	KX966097	This study
	Kottigehara, Karnataka	SDBDU 2010.128	KX966098	This study
	Shirva, Karnataka	RBRL 060715-02	AB530593	Hasan *et al*. 2014
	–	AA200	JQ596663	Nair *et al*. 2012
	–	AA220	JQ596664	Nair *et al*. 2012
	–	AA227	JQ596665	Nair *et al*. 2012
	–	AA230	JQ596666	Nair *et al*. 2012
	–	AA231	JQ596667	Nair *et al*. 2012
	India	FB2000b	AF249064	Bossuyt & Milinkovitch 2000
	Mookambika	WILD-15-AMP-630	KX641815	Dahanukar *et al*. 2016
	Mookambika	WILD-15-AMP-631	KX641816	Dahanukar *et al*. 2016
	Muduba	BNHS 5980	KX641817	Dahanukar *et al*. 2016
Indirana gundia	Aralam WLS, Kerala	SDBDU 2011.1056	KX966099	This study
	Aralam WLS, Meenmuthy, Kerala	SDBDU 2008.432	KX966100	This study
	Gundia, Karnataka	SDBDU 2010.007	KX966101	This study
	Gundya, Karnataka	WILD-14-AMP-499	KM386532	Dahanukar *et al*. 2016
	Gundya, Karnataka	WILD-14-AMP-500	KM386533	Dahanukar *et al*. 2016
	Kempholey, Karnataka	SDBDU 2008.433	KX966102, KX966168	This study
	Monnangeri, Karnataka	SDBDU 2006.1436	KX966103	This study
	Monnangeri, Karnataka	SDBDU 2006.1438	KX966104	This study
	–	AA175	JQ596648	Nair *et al*. 2012
	–	AA178	JQ596649	Nair *et al*. 2012
	–	AA180	JQ596650	Nair *et al*. 2012
	–	AA189	JQ596651	Nair *et al*. 2012
	–	AA193	JQ596652	Nair *et al*. 2012
	Aralam	WILD-13-AMP-139	KX641783	Dahanukar *et al*. 2016
	Coorg	WILD-13-AMP-210	KX641784	Dahanukar *et al*. 2016
	Kutta	WILD-13-AMP-211	KX641785	Dahanukar *et al*. 2016
	Aralam	WILD-13-AMP-136	KX641786	Dahanukar *et al*. 2016
	Ranipuram	WILD-15-AMP-614	KX641787	Dahanukar *et al*. 2016
	Ranipuram	WILD-15-AMP-616	KX641788	Dahanukar *et al*. 2016
	Ranipuram	WILD-15-AMP-618	KX641789	Dahanukar *et al*. 2016
	Subramanya Sullya	WILD-16-AMP-649	KX641790	Dahanukar *et al*. 2016
Indirana leithii	Bhimashankar, Maharashtra	SDBDU 2011.1100	KX966105	This study
	Dhobi falls, Mahabaleshwar,	SDBDU 2007.6062	KX966106	This study

	Maharashtra			
	Matheran, Maharashtra	SDBDU 2005.1237	KX966107	This study
	Matheran, Maharashtra	SDBDU 2012.2094	KX966108, KX966169	This study
	Matheran, Maharashtra	SDBDU 2014.2513	KX966109	This study
	Matheran, Maharashtra	BNHS 5590	KF590637	Modak *et al*. 2014
	Matheran, Maharashtra	BNHS 5591	KF590638	Modak *et al*. 2014
	Ratangad, Maharashtra	AGCZRL-Amphibia-112	KF590645	Modak *et al*. 2014
	Ratangad, Maharashtra	AGCZRL-Amphibia-113	KF590646	Modak *et al*. 2014
	Harishchandragad, Maharashtra	WILD-013-AMP-173	KF590639	Modak *et al*. 2014
	Harishchandragad, Maharashtra	WILD-013-AMP-174	KF590640	Modak *et al*. 2014
	Tamhini, Maharashtra	WILD-013-AMP-175	KF590641	Modak *et al*. 2014
	Tamhini, Maharashtra	WILD-013-AMP-176	KF590642	Modak *et al*. 2014
	Koynanagar, Maharashtra	WILD-013-AMP-177	KF590643	Modak *et al*. 2014
	Visapur, Maharashtra	WILD-013-AMP-178	KF590644	Modak *et al*. 2014
	Karnala	WILD-15-AMP-525	KX641791	Dahanukar *et al*. 2016
	Javalya fort	AGCZRL-Amphibia-549	KX641792	Dahanukar *et al*. 2016
	Achala fort	AGCZRL-Amphibia-548	KX641793	Dahanukar *et al*. 2016
	Ahwa Dang	AGCZRL-Amphibia-552	KX641794	Dahanukar *et al*. 2016
	Ahwa Dang	AGCZRL-Amphibia-555	KX641795	Dahanukar *et al*. 2016
Indirana paramakri sp. nov.	Settukunnu, Wayanad, Kerala	ZSI/WGRC/V/A889	KX966110, KX966170	This study
	Suganthagiri, Wayanad, Kerala	ZSI/WGRC/V/A890	KX966111	This study
	Suganthagiri, Wayanad, Kerala	SDBDU 2005.3740	KX966112	This study
	–	AA91	JQ596653	Nair *et al*. 2012
	–	AA92	JQ596654	Nair *et al*. 2012
	–	AA93	JQ596655	Nair *et al*. 2012
	–	AA94	JQ596656	Nair *et al*. 2012
	–	AA98	JQ596657	Nair *et al*. 2012
Indirana salelkari	Dandeli, Karnataka	SDBDU 2011.1330	KX966113, KX966171	This study
	Jog falls, Karnataka	SDBDU 2003.40180	KX966114	This study
	Unchali falls, Karnataka	SDBDU 2012.1316	KX966115	This study
	Neturlim, Sanguem, Karnataka	BNHS 5931	KP826824	Modak *et al*. 2015
	Neturlim, Sanguem, Karnataka	WILD-15-AMP-551	KP826825	Modak *et al*. 2015
	Neturlim, Sanguem, Karnataka	AGCZRL Amphibia 210	KP826826	Modak *et al*. 2015
Indirana sarojamma	Chathankod–Bonnacaud, Kerala	SDBDU 2002.516	KX966116	This study
	Ponmudi, Kerala	SDBDU 2005.1245	KX966117	This study
	Ponmudi, Kerala	SDBDU 2013.2310	KX966118, KX966172	This study
	–	AA800	JQ596677	Nair *et al*. 2012
	Ponmudi	BNHS 5981	KX641796	Dahanukar *et al*. 2016
Indirana semipalmata	Chathankod–Bonnacaud, Kerala	SDBDU 2002.520	KX966119	This study
	Double cut, Kattapana, Kerala	SDBDU 2008.443	KX966120	This study
	Gavi, Kerala	SDBDU 2006.4819	KX966121, KX966173	This study

	Kaikatti, Nelliyampathy, Kerala	SDBDU 2005.26	KX966122	This study
	Kallar, Kerala	SDBDU 2006.4773A	KX966123	This study
	Kallar, Kerala	SDBDU 2006.4773B	KX966124	This study
	Kulamav, Kerala	SDBDU 2010.201	KX966125	This study
	Nenmara, Kerala	SDBDU 2011.1133	KX966126	This study
	Neriamangalam, Kerala	SDBDU 2011.1125	KX966127	This study
	Pakuthipaalam, Nelliyampathy, Kerala	SDBDU 2002.527	KX966128	This study
	Pampadumpara, Kerala	SDBDU 2008.337	KX966129	This study
	Parambikulam TR, Kerala	SDBDU 2015.3033	KX966130	This study
	Siruvani, Singappara, Kerala	SDBDU 2015.3014	KX966131	This study
	Thekkady, Periyar TR, Kerala	SDBDU 2012.1824	KX966132	This study
	Top Slip, Karian Shola, Tamil Nadu	SDBDU 2005.11	KX966133	This study
	–	AA255	JQ596670	Nair *et al*. 2012
	–	AA256	JQ596671	Nair *et al*. 2012
	–	AA257	JQ596672	Nair *et al*. 2012
	–	AA243	JQ596668	Nair *et al*. 2012
	–	AA245	JQ596669	Nair *et al*. 2012
	Sholayar	WILD-15-AMP-610	KX641797	Dahanukar *et al*. 2016
	Sholayar	WILD-15-AMP-611	KX641798	Dahanukar *et al*. 2016
	Kunthipuzha	WILD-14-AMP-419	KX641799	Dahanukar *et al*. 2016
	Kizhukanam	WILD-14-AMP-438	KX641800	Dahanukar *et al*. 2016
	Shendurney	WILD-13-AMP-269	KX641801	Dahanukar *et al*. 2016
	Shendurney	WILD-13-AMP-270	KX641802	Dahanukar *et al*. 2016
	Shendurney	WILD-13-AMP-271	KX641803	Dahanukar *et al*. 2016
	Shendurney	WILD-13-AMP-296	KX641804	Dahanukar *et al*. 2016
	Idukki	WILD-14-AMP-351	KX641805	Dahanukar *et al*. 2016
	Silent Valley	WILD-14-AMP-416	KX641806	Dahanukar *et al*. 2016
	Peechi-Vazhani	WILD-14-AMP-470	KX641807	Dahanukar *et al*. 2016
	Chimmony	WILD-14-AMP-471	KX641808	Dahanukar *et al*. 2016
	Peechi-Vazhani	WILD-14-AMP-472	KX641809	Dahanukar *et al*. 2016
	Chimmony	WILD-14-AMP-473	KX641810	Dahanukar *et al*. 2016
	Chimmony	WILD-14-AMP-474	KX641811	Dahanukar *et al*. 2016
	Parambikulam	WILD-14-AMP-503	KX641812	Dahanukar *et al*. 2016
	Painavu	WILD-14-AMP-354	KX641813	Dahanukar *et al*. 2016
	Idukki	WILD-14-AMP-440	KX641814	Dahanukar *et al*. 2016
Indirana tysoni	Bhagamandla, Karnataka	SDBDU 2012.102	KX966134	This study
	Charmadi Ghats, Karnataka	SDBDU 2011.1395	KX966135	This study
	Madikeri, Abby falls, Karnataka	SDBDU 2006.1443	KX966136	This study
	Thalakaveri, Karnataka	SDBDU 2007.5093	KX966137	This study
	Yavakapady, Coorg, Karnataka	SDBDU 2012.2228	KX966138	This study
	Yavakapady, Coorg, Karnataka	SDBDU 2012.74	KX966139, KX966174	This study
	Ranipuram	BNHS 5979	KX641818	Dahanukar *et al*. 2016
	Ranipuram	WILD-15-AMP-615	KX641819	Dahanukar *et al*. 2016
	Wattakole	WILD-16-AMP-650	KX641820	Dahanukar *et al*. 2016
Indirana yadera	Kozhikana, Periyar TR, Kerala	SDBDU 2015.2984	KX966140	This study
	Methooty, Kerala	SDBDU 2012.2744	KX966141, KX966175	This study
	Neriamangalam, Kerala	SDBDU 2011.1120	KX966142	This study
	Neriamangalam, Kerala	SDBDU 2011.1124	KX966143	This study
	Nilakkal, Kerala	SDBDU 2006.4852	KX966144	This study
	Vazhachal, Kerala	SDBDU 2015.3155	KX966145	This study
	–	AA244	JQ596674	Nair *et al*. 2012
	–	AA246	JQ596675	Nair *et al*. 2012
	–	AA724	JQ596676	Nair *et al*. 2012

	Neyyar	WILD-13-AMP-338	KX641821	Dahanukar *et al*. 2016
	Vagamalai	BNHS 5982	KX641822	Dahanukar *et al*. 2016
	Chimmony	WILD-14-AMP-479	KX641823	Dahanukar *et al*. 2016
Sallywalkerana diplosticta	Athirimala, Kerala	SDBDU 2002.512	KX966146	This study
	Athirimala, Kerala	SDBDU 2003.40103	KX966147	This study
	Pandimotta, Shendurney WLS, Kerala	SDBDU 2011.283	KX966148	This study
	Pandimotta, Shendurney WLS, Kerala	SDBDU 2011.284	KX966149	This study
	Pandipath, Agasthyamala Hills, Kerala	SDBDU 2015.2956	KX966150, KX966176	This study
	–	AA791	JQ596678	Nair *et al*. 2012
	–	AA793	JQ596679	Nair *et al*. 2012
	–	AA809	JQ596680	Nair *et al*. 2012
	Bonnacaud	WILD-15-AMP-640	KX641828	Dahanukar *et al*. 2016
Sallywalkerana leptodactyla	Anamudi, Eravikulam NP, Kerala	SDBDU 2002.1026	KX966151	This study
	Eravikulam NP, Kerala	SDBDU 2011.1056A	KX966152, KX966177	This study
	Mattupetti, Kerala	SDBDU 2013.911	KX966153	This study
	Munnar, Kerala	SDBDU 2005.31	KX966154	This study
	Ponkalapara, Kerala	SDBDU 2007.4330	KX966155	This study
	Vagvarai, Eravikulam NP, Kerala	SDBDU 2009.1971	KX966156	This study
	Andiparai shola, Valparai, Tamil Nadu	SDBDU 2002.1176	KX966157	This study
	Grass Hills, Tamil Nadu	SDBDU 2005.19	KX966158	This study
	Kodaikanal, Tamil Nadu	SDBDU 2002.916	KX966159	This study
	Kodaikanal, Tamil Nadu	SDBDU 2002.917	KX966160	This study
	Valparai, Tamil Nadu	SDBDU 2004.40336	KX966161	This study
	India	FB-2000a	AF249051	Bossuyt & Milinkovitch 2000
	India	VUB 0037	JN644772	Van Bocxlaer *et al.* 2011
	Eravikulam	WILD-13-AMP-192	KX641825	Dahanukar *et al*. 2016
	Eravikulam	WILD-13-AMP-186	KX641826	Dahanukar *et al*. 2016
	Eravikulam	WILD-13-AMP-184	KX641827	Dahanukar *et al*. 2016
Sallywalkerana phrynoderma	Grass Hills, Akkamalai shola, Tamil Nadu	SDBDU 2002.1181	KX966162, KX966178	This study
	Anamalai	WILD-14-AMP-509	KX641824	Dahanukar *et al*. 2016
**Outgroup**				
Nyctibatrachus sp.	Nelliyampathy, Kerala	SDBDU 2011.1138	KJ711394, KX966179	Biju *et al*. 2014a, This study
