# Supplementary material for: Molecular and Morphological Study of Leaping Frogs (Anura, Ranixalidae) with Description of Two New Species
Source: PLoS One. 2016 Nov 16;11(11):e0166326. doi: 10.1371/journal.pone.0166326 (PMC5112961; doi:10.1371/journal.pone.0166326)
Supplement: S3 Table — The table gives mean and standard deviation values over all pairwise comparisons among individuals or populations of a species. N is the number of individuals for each species. The original p-distances are shown in percentage. (PDF) [file pone.0166326.s013.pdf]

Supporting information: Tables

Molecular and morphological study of Leaping frogs (Anura, Ranixalidae) with description of two new species

Sonali Garg, SD Biju

S3 Table. Uncorrected intraspecific pairwise distances between 16S mitochondrial gene sequences. The table gives mean and standard deviation values over all pairwise comparisons among individuals or populations of a species. N is the number of individuals for each species. The original p-distances are shown in percentage.

Species	N	Mean	SD	Min	Max
Indirana beddomii	22	1.2	0.9	0.0	3.5
Indirana bhadrai	1	–	–	–	–
Indirana brachytarsus	64	0.4	0.3	0.0	1.5
Indirana chiravasi	17	0.1	0.1	0.0	0.4
Indirana duboisi	19	0.5	0.3	0.0	1.3
Indirana gundia	21	0.2	0.2	0.0	0.7
Indirana leithii	20	0.7	0.7	0.0	1.7
Indirana paramakri	8	0.6	0.4	0.0	1.3
Indirana salelkari	6	1.2	0.9	0.0	2.2
Indirana sarojamma	5	0.2	0.2	0.0	0.4
Indirana semipalmata	38	1.1	0.7	0.0	2.4
Indirana tysoni	9	0.0	0.0	0.0	0.0
Indirana yadera	12	0.4	0.3	0.0	1.1
Sallywalkerana diplosticta	9	1.1	0.6	0.0	1.7
Sallywalkerana leptodactyla	16	0.3	0.3	0.0	1.4
Sallywalkerana phrynoderma	2	0.0	–	0.0	0.0
