# Supplementary material for: Molecular and Morphological Study of Leaping Frogs (Anura, Ranixalidae) with Description of Two New Species
Source: PLoS One. 2016 Nov 16;11(11):e0166326. doi: 10.1371/journal.pone.0166326 (PMC5112961; doi:10.1371/journal.pone.0166326)
Supplement: S4 Table — The table gives mean and standard deviation values over all pairwise comparisons of individuals sequenced from the two taxa being compared. N is the number of pairwise comparisons. N1 and N2 represent number of individuals for Taxon 1 and Taxon 2, respectively. The original p-distances are shown in percentage. (PDF) [file pone.0166326.s014.pdf]

Supporting information: Tables

**Molecular and morphological study of Leaping frogs (Anura, Ranixalidae) with description of two new species**

Sonali Garg, SD Biju

**S4 Table. Uncorrected interspecific pairwise distances between 16S mitochondrial gene sequences.** The table gives mean and standard deviation values over all pairwise comparisons of individuals sequenced from the two species being compared. N is the number of pairwise comparisons. N1 and N2 represent number of individuals for Taxon 1 and Taxon 2, respectively. The original p-distances are shown in percentage.

| Taxon 1 | Taxon 2 | N1 | N2 | N | Mean ± SD | Min | Max |
|---|---|---|---|---|---|---|---|
| ***Indirana beddomii* GROUP** | | | | | | | |
| *Indirana beddomii* | *Indirana bhadrai* | 22 | 1 | 22 | 4.2 ± 0.3 | 3.7 | 5.0 |
| *Indirana beddomii* | *Indirana brachytarsus* | 22 | 64 | 1408 | 6.3 ± 0.4 | 5.5 | 7.7 |
| *Indirana beddomii* | *Indirana leithii* | 22 | 20 | 440 | 4.9 ± 0.7 | 3.4 | 6.8 |
| *Indirana beddomii* | *Indirana sarojamma* | 22 | 5 | 110 | 5.5 ± 0.4 | 5.0 | 7.0 |
| *Indirana beddomii* | *Indirana tysoni* | 22 | 9 | 198 | 4.7 ± 0.5 | 4.0 | 5.9 |
| *Indirana beddomii* | *Indirana yadera* | 22 | 12 | 264 | 6.2 ± 0.5 | 5.5 | 8.5 |
| *Indirana bhadrai* | *Indirana brachytarsus* | 1 | 64 | 64 | 5.8 ± 0.2 | 5.5 | 6.6 |
| *Indirana bhadrai* | *Indirana leithii* | 1 | 20 | 20 | 4.7 ± 0.7 | 3.9 | 5.8 |
| *Indirana bhadrai* | *Indirana sarojamma* | 1 | 5 | 5 | 5.1 ± 0.4 | 4.9 | 5.7 |
| *Indirana bhadrai* | *Indirana tysoni* | 1 | 9 | 9 | 3.1 ± 0.1 | 3.0 | 3.1 |
| *Indirana bhadrai* | *Indirana yadera* | 1 | 12 | 12 | 5.6 ± 0.2 | 5.3 | 6.1 |
| *Indirana brachytarsus* | *Indirana leithii* | 64 | 20 | 1280 | 6.7 ± 0.6 | 5.6 | 7.9 |
| *Indirana brachytarsus* | *Indirana sarojamma* | 64 | 5 | 320 | 8.0 ± 0.4 | 7.5 | 9.0 |
| *Indirana brachytarsus* | *Indirana tysoni* | 64 | 9 | 576 | 6.9 ± 0.2 | 6.4 | 7.7 |
| *Indirana brachytarsus* | *Indirana yadera* | 64 | 12 | 768 | 8.7 ± 0.3 | 8.1 | 9.6 |
| *Indirana leithii* | *Indirana sarojamma* | 20 | 5 | 100 | 6.2 ± 0.6 | 5.5 | 7.3 |
| *Indirana leithii* | *Indirana tysoni* | 20 | 9 | 180 | 6.1 ± 0.7 | 5.2 | 7.1 |
| *Indirana leithii* | *Indirana yadera* | 20 | 12 | 240 | 7.0 ± 0.7 | 6.1 | 8.2 |
| *Indirana sarojamma* | *Indirana tysoni* | 5 | 9 | 45 | 5.8 ± 0.2 | 5.6 | 6.1 |
| *Indirana sarojamma* | *Indirana yadera* | 5 | 12 | 60 | 3.5 ± 0.1 | 3.3 | 3.7 |
| *Indirana tysoni* | *Indirana yadera* | 9 | 12 | 108 | 5.9 ± 0.1 | 5.6 | 6.1 |
| ***Indirana semipalmata* GROUP** | | | | | | | |
| *Indirana chiravasi* | *Indirana duboisi* | 17 | 19 | 323 | 3.0 ± 0.2 | 2.7 | 3.7 |
| *Indirana chiravasi* | *Indirana gundia* | 17 | 21 | 357 | 3.9 ± 0.3 | 3.5 | 4.6 |
| *Indirana chiravasi* | *Indirana paramakri* | 17 | 8 | 136 | 6.7 ± 0.5 | 6.1 | 7.8 |
| *Indirana chiravasi* | *Indirana salelkari* | 17 | 6 | 102 | 3.2 ± 0.4 | 2.5 | 3.7 |
| *Indirana chiravasi* | *Indirana semipalmata* | 17 | 38 | 646 | 5.1 ± 0.4 | 4.3 | 6.4 |
| *Indirana duboisi* | *Indirana gundia* | 19 | 21 | 399 | 3.2 ± 0.3 | 2.4 | 3.9 |
| *Indirana duboisi* | *Indirana paramakri* | 19 | 8 | 152 | 5.4 ± 0.6 | 4.5 | 7.0 |
| *Indirana duboisi* | *Indirana salelkari* | 19 | 6 | 114 | 2.6 ± 0.3 | 2.1 | 3.3 |
| *Indirana duboisi* | *Indirana semipalmata* | 19 | 38 | 722 | 4.7 ± 0.3 | 4.2 | 5.6 |
| *Indirana gundia* | *Indirana paramakri* | 21 | 8 | 168 | 5.2 ± 0.5 | 4.2 | 6.3 |
| *Indirana gundia* | *Indirana salelkari* | 21 | 6 | 126 | 4.0 ± 0.3 | 3.5 | 4.4 |
| *Indirana gundia* | *Indirana semipalmata* | 21 | 38 | 798 | 3.3 ± 0.2 | 2.6 | 3.9 |
| *Indirana paramakri* | *Indirana salelkari* | 8 | 6 | 48 | 6.1 ± 0.5 | 5.5 | 7.0 |
| *Indirana paramakri* | *Indirana semipalmata* | 8 | 38 | 304 | 4.5 ± 0.5 | 3.7 | 5.9 |
| *Indirana salelkari* | *Indirana semipalmata* | 6 | 38 | 228 | 5.4 ± 0.5 | 4.3 | 6.3 |
| ***Sallywalkerana*** | | | | | | | |
| *Sallywalkerana diplosticta* | *Sallywalkerana leptodactyla* | 9 | 16 | 144 | 11.2 ± 0.4 | 10.0 | 12.7 |
| *Sallywalkerana diplosticta* | *Sallywalkerana phrynoderma* | 9 | 2 | 18 | 12.2 ± 0.5 | 11.5 | 12.7 |
| *Sallywalkerana leptodactyla* | *Sallywalkerana phrynoderma* | 16 | 2 | 32 | 6.6 ± 0.3 | 5.9 | 7.6 |
